# RNASEH2C enhances TRAF3IP1 to degrade RAI14 in lysosomes thus hindering macrophage antigen presentation and advancing liver cancer

**DOI:** 10.1038/s41419-025-08305-5

**Published:** 2025-12-08

**Authors:** Banglun Pan, Huahui Yu, Zikun Lin, Mengxin Liu, Jiayu Liu, Yiqing Xu, Linqing Wu, Qiuyu Zhang, Zengbin Wang

**Affiliations:** 1 The School of Basic Medical Sciences, Fujian Medical University, Fuzhou, 350122 China; 2 Laboratory Medicine, Mengchao Hepatobiliary Hospital of Fujian Medical University, Fuzhou, 350028 China; 3 Department of Hepatobiliary Surgery and Fujian Institute of Hepatobiliary Surgery, Fujian Medical University Union Hospital, Fuzhou, 350001 China; 4 Institute of Immunotherapy, Fujian Medical University, Fuzhou, 350122 China

**Keywords:** MHC, Gastrointestinal cancer

## Abstract

Macrophage antigen presentation is crucial for adaptive immunity and maintaining immune balance, including anti-infection, anti-tumor, and inflammation regulation. However, its role in tumor immunomodulation is less understood compared to macrophage polarization. This study explored how *Rnaseh2c*^+^ macrophages influence hepatocellular carcinoma (HCC) progression using in vitro cell models and mouse tumor models. Single-cell RNA sequencing, immunoblotting, immunofluorescence, immunoprecipitation, and flow cytometry analysis were employed to examine RNASEH2C’s impact on macrophage antigen presentation. Our results indicated that *Rnaseh2c*^+^ macrophages, which were non-polarized, promoted HCC growth by inhibiting antigen presentation. RNASEH2C facilitated lysosomal degradation of RAI14 by enhancing TRAF3IP1 expression and suppressing the mTOR pathway, with HSC70 and CMTM6 playing opposing roles in RAI14 degradation. RAI14, a skeleton protein, facilitated the macropinocytosis of MHC II molecules and tumor-associated antigen, thus activating Th1 cells in HCC. In conclusion, our study revealed how RNASEH2C mediated RAI14’s lysosomal degradation, offering potential targets and strategies for HCC immunotherapy.

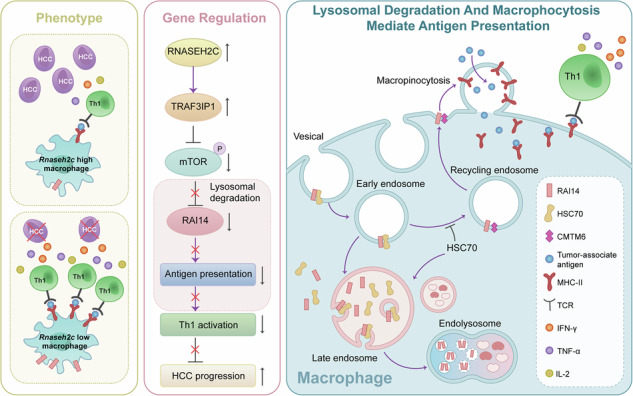

## Introduction

Macrophages are crucial in the tumor microenvironment by presenting antigens via MHC II molecules, which activates Th1 cells, promoting an anti-tumor immune response [1]. This antigen presentation is dynamic and influenced by macrophages’ polarization, metabolism, microenvironmental signals, and pathogen traits [2]. Disruptions in these processes cause immune deficiencies, autoimmune diseases, or tumor immune escape [2]. Targeting macrophage antigen presentation, using IFN-γ or small molecule inhibitors, is key to the development of new vaccines and immunotherapies [3]. However, tumor cells inhibit this function by secreting IL-10 and TGF-β and expressing PD-L1/L2 [1]. Therefore, enhancing macrophage antigen presentation is a key strategy to boost therapy. This study aimed to identify molecules that regulate this process and explored ways to modulate their function to enhance anti-tumor immunity.

Macropinocytosis is intricately associated with antigen presentation [4]. Dendritic cells, renowned for their exceptional antigen-presenting capabilities, utilize endocytosis to internalize extracellular antigens or MHC I molecules, owing to their robust internalization capacity [4]. The non-specific uptake of soluble antigens via macropinocytosis is essential for the detection of pathogenic microorganisms and tumor-associated antigens [4]. Nevertheless, the involvement of macrophage-mediated macropinocytosis in the uptake of antigens and MHC II molecules remains unclear.

Tumor microenvironment hinders macrophage antigen presentation by altering lysosomal activity, aiding immune escape [5–7]. The key molecules in this process include HSC70 and CMTM6 [6, 7]. HSC70, found on the lysosomal membrane, functions as a molecular chaperone aiding in protein folding, transport, and quality control by binding ATP to move substrate proteins into the lysosomal cavity [7]. CMTM6 is a transmembrane protein that influences lysosomal membrane permeability and pH stability, thereby inhibiting lysosomal degradation [6]. However, it is uncertain if HSC70 and CMTM6 impact macrophage tumor immunity through lysosomal degradation regulation.

This study revealed that *Rnaseh2c*^+^ macrophages, distinct from M1 or M2 types, boost lysosomal degradation of RAI14 by up-regulating TRAF3IP1 expression and inhibiting the mTOR pathway. Subsequently, HSC70 aided in RAI14 degradation via the endosome-lysosome pathway, where CMTM6 competed with HSC70 to bind RAI14, inhibiting its degradation. Furthermore, RAI14, a skeleton protein, promoted macropinocytosis, aiding in MHC II molecules uptake and enhancing the adaptive immune response. Therefore, this finding provided a pathway for the development of future cancer immunotherapies that enhance macrophage antigen presentation.

## Methods and materials

### Study design and group

Details regarding the number of biological replicates, the statistical methods employed, and the *P* values were provided in the figure legends. Sample sizes were not predetermined using statistical methods; instead, they were estimated based on preliminary experiments. All in vitro experiments were conducted a minimum of three times, with no exclusion of outliers or other data points from our analyses. It should be noted that since we have conditionally knocked in (cKI) or knocked out (cKO) mice with *Rnaseh2c*, *Traf3ip1*, or *Rai14*, when conducting cell experiments to manipulate these genes, we directly sorted macrophages in transgenic mice for experiments. Because we did not have transgenic mice for *Hsc70*, *Cmtm6*, or *Lamp2a*, we performed in vitro experiments with mouse macrophage line RAW264.7 cells. Animal experiments were conducted at least five times, with mice being randomly assigned to treatment groups by cage. Investigators were blinded to group assignments during the experiments and outcome assessments. Given the post hoc nature of the study, the analyses presented should be considered exploratory. There was no attrition in this study. Because this was a pilot study, a formal power calculation was not required.

### Mouse HCC-infiltrating macrophage treatment strategies

Various mouse hepatocellular carcinoma (HCC) models were constructed (**Mouse model**) in Supplementary Information. Following the sacrifice of mice, primary HCC tissues were dissociated into single cell suspensions (**Isolation of tumor tissue**) in Supplementary Information. Subsequently, live macrophages were isolated through flow cytometry sorting (**Flow cytometry sorting**) in Supplementary Information. Cell transfection, immunoblotting, immunoprecipitation, and qPCR were then employed.

### Statistical analysis

We used SPSS 19.0 (IBM, NY, USA) and R 4.4 software for statistical analysis, and GraphPad Prism 9.0 (GraphPad Software, CA, USA) and R 4.4 software to generate visual images. All data were expressed as mean ± SD and *P* value < 0.05 were considered statistically significant. Normality analyses were conducted using Shapiro-Wilk test. Homogeneity of variance analyses were conducted using Levene test. When comparing two samples, the independent sample *t* test was used if the data were normally distributed, and the variances were homogeneous. Otherwise, Wilcoxon rank sum test was used. Kaplan-Meier curves with 95% confidence intervals were plotted and the log-rank test was used to compare survival curves.

Additional methods were listed in Supplemental Information.

## Results

### RNASEH2C promoted tumor growth by inhibiting the mTOR pathway

**Fig. 1:**
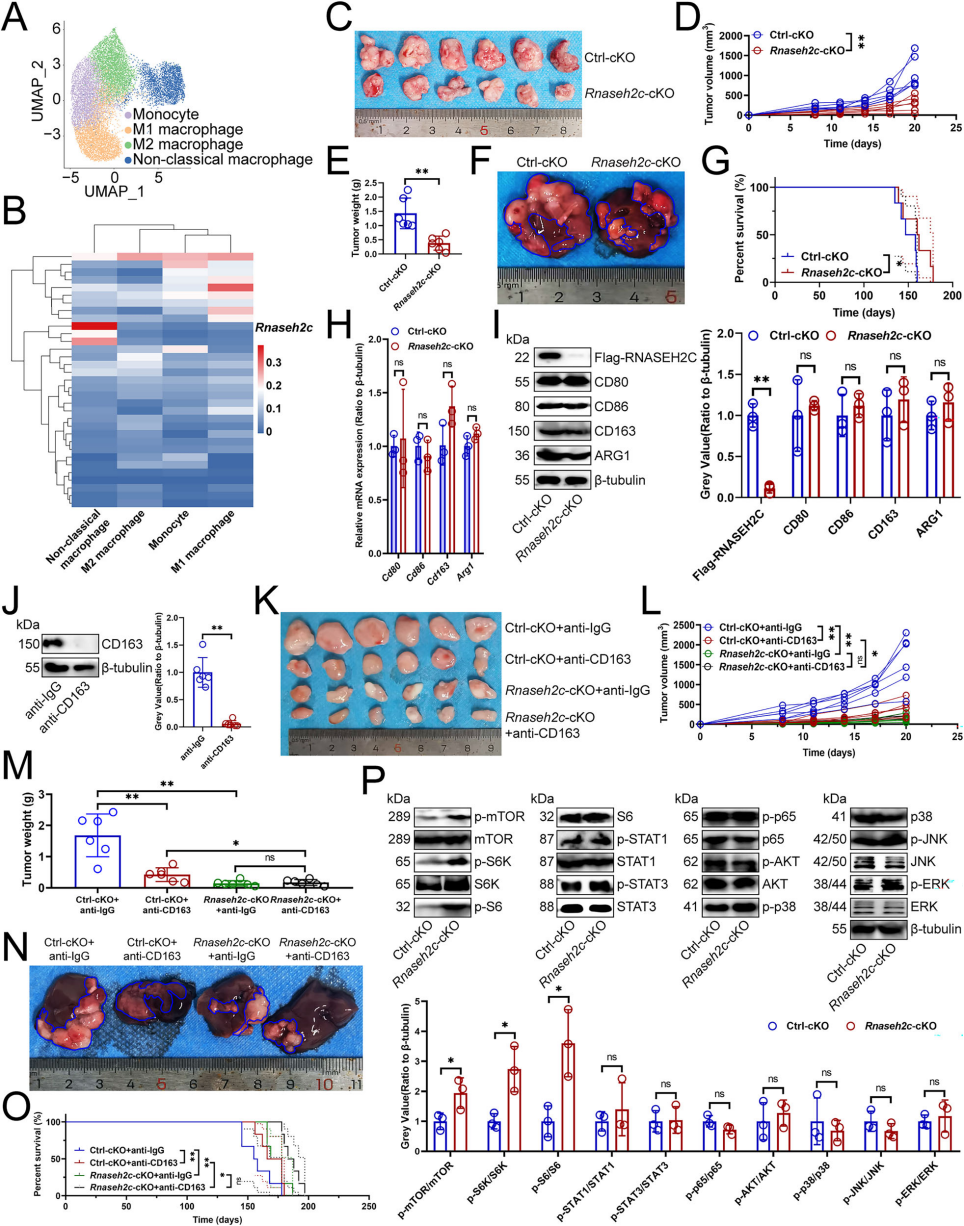
RNASEH2C promoted tumor growth by inhibiting the mTOR pathway. **A** Single-macrophage embeddings with color-coded clusters (*n* = 4). Mouse primary HCC was constructed and tumor tissues were collected for single-cell RNA sequencing. **B** SCENIC analysis for mouse HCC-infiltrating macrophages (*n* = 4). **C–E** Effects of *Rnaseh2c*-cKO on subcutaneous tumor growth (*n* = 6). Wild-type Hep-53.4 cells were implanted into the left axillary region of mice. Tumor volume was measured regularly, and mice were euthanized when the diameter of any tumor exceeded 2 cm; the tumors were then excised and weighed. **C** Representative. **D** Tumor growth curve. **E** Weight of the tumor. **F**, **G** Effects of *Rnaseh2c*-cKO on primary carcinoma growth (*n* = 6). DEN and CCl_4_ were infused into mice to construct primary cancers, with observations made on the mice’s condition and survival time recorded. Upon the death of the mice, HCC tissues were collected for further analysis. **F** Representative. **G** Survival curve. qPCR (**H**) and immunoblotting (**I**) analyzing the effect of *Rnaseh2c*-cKO on the expression of macrophage polarization markers in mouse HCC-infiltrating macrophages (*n* = 3). **J** Immunoblotting analysis of the protein expression of CD163 in tumor tissues (*n* = 6). Mice were injected with anti-CD163 antibody through tail vein to neutralize M2 macrophages. **K–M** Effects of *Rnaseh2c*-cKO and anti-CD163 antibody on subcutaneous tumor growth (*n* = 6). **K** Representative. **L** Tumor growth curve. **M** Weight of the tumor. **N**, **O** Effects of *Rnaseh2c*-cKO and anti-CD163 antibody on primary carcinoma growth (*n* = 6). **N** Representative. **O** Survival curve. **P** Immunoblotting exploring the effect of *Rnaseh2c*-cKO on macrophage related signaling pathway protein expression in mouse HCC-infiltrating macrophages (*n* = 3). **D**, **E**, **H**, **L**, **M** represented mean ± SD analyzed by Wilcoxon test, **I**, **J**, **P** represented mean ± SD analyzed by unpaired *t* test, **G**, **O** were analyzed by log-rank test. *P < 0.05, **P < 0.01. cKO conditional knockout, HCC hepatocellular carcinoma, SCENIC single-cell regulatory network inference and clustering.

Our single-cell RNA sequencing analysis [8] of the monocyte-macrophage subpopulation in mouse primary HCC identified monocytes, M1, M2, and non-classical macrophages, with non-classical macrophages characterized by low *Cd80* expression and an absence of *Cd163* expression (Fig. 1A, S1A, S1B). In our study, M2 macrophages were characterized by the presence of anti-inflammatory markers *Gas6* [9], *Ch25h* [10], and *Olfml3* [11], as well as the pro-cancer marker *Hpgds* [12] (Fig. S1A). Monocytes were identified using *Cd200r1* and *Csf1r* [13] (Fig. S1A). Additionally, the co-activation marker *Cd86*, alongside pro-inflammatory markers *Cd14* [14] and *Plac8* [15], were employed to define M1 macrophages (Fig. S1A). Importantly, these non-classical macrophages did not highly express the other markers of M1 macrophages (*Cd86*, *Il6*) or M2 macrophages (*Cd206*, *Tgfb1*) (Fig. S1A). Using Single-Cell Regulatory Network Inference and Clustering (SCENIC) [16], we identified RNASEH2C as the specific transcription factor for this non-classical macrophage subpopulation (Fig. 1B), and thus we named them *Rnaseh2c*^+^ macrophages. To identify the tumor-modulating role of *Rnaseh2c*^+^ macrophages, we conditionally knocked *Rnaseh2c* out on macrophages and found *Rnaseh2c*-cKO significantly inhibited tumor growth and extended survival compared to controls (Fig. 1C-G). Despite M2 macrophages’ similar tumor-promoting role, *Rnaseh2c*-cKO did not affect M1 (CD80, CD86) or M2 (CD163, ARG1) marker expression in mouse HCC-infiltrating macrophages (Fig. 1H, I). Further, we cleared M2 macrophages with anti-CD163 antibody (Fig. 1J) to analyze whether RNASEH2C was dependent on M2 macrophages for its cancer-promoting effects and found both *Rnaseh2c*-cKO and this antibody inhibited tumor growth and extended survival, with *Rnaseh2c*-cKO still effective in mice lacking M2 macrophages (Fig. 1K-O). This suggested RNASEH2C’s pro-tumor effects were independent of M2 macrophages. To investigate the mechanism, we analyzed changes in the signaling pathway closely associated with the immunomodulatory function of macrophages [2] and found that *Rnaseh2c*-cKO specifically upregulated the mTOR pathway without affecting other pathways in mouse HCC-infiltrating macrophages (Fig. 1P). Therefore, we further examined how RNASEH2C inhibits the mTOR pathway and whether this is key to its tumor-promoting role.

### RNASEH2C promoted tumor growth by up-regulating TRAF3IP1 and inhibiting the mTOR pathway

**Fig. 2:**
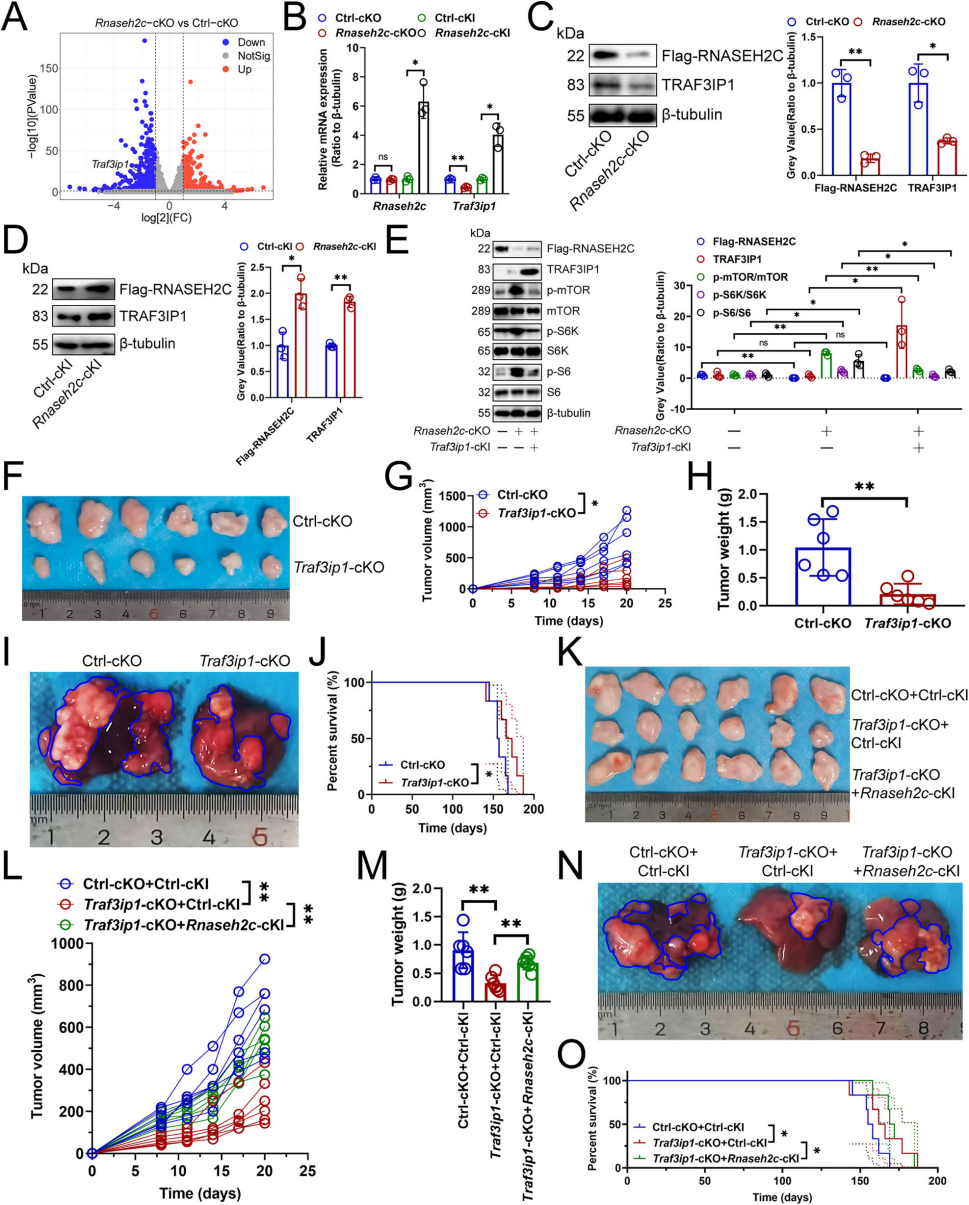
RNASEH2C promoted tumor growth by up-regulating TRAF3IP1 expression and inhibiting the mTOR pathway. **A** RNA-seq analysis of the effect of *Rnaseh2c*-cKO on transcriptome in mouse HCC-infiltrating macrophages (*n* = 3). **B** qPCR analyzing the effect of *Rnaseh2c*-cKO or -cKI on *Traf3ip1* transcript expression in mouse HCC-infiltrating macrophages (*n* = 3). Immunoblotting demonstrating the effect of *Rnaseh2c*-cKO (**C**) or -cKI (**D**) on TRAF3IP1 protein expression in mouse HCC-infiltrating macrophages (*n* = 3). **E** Immunoblotting illustrating the effect of *Rnaseh2c*-cKO and *Traf3ip1*-cKI on the expression of proteins related to the mTOR pathway in mouse HCC-infiltrating macrophages (*n* = 3). **F–H** Effects of *Traf3ip1*-cKO on subcutaneous tumor growth (*n* = 6). Wild-type Hep-53.4 cells were implanted into the left axillary region of mice. Tumor volume was measured regularly, and mice were euthanized when the diameter of any tumor exceeded 2 cm; the tumors were then excised and weighed. **F** Representative. **G** Tumor growth curve. **H** Weight of the tumor. **I**, **J** Effects of *Traf3ip1*-cKO on primary carcinoma growth (*n* = 6). DEN and CCl_4_ were infused into mice to construct primary cancers, with observations made on the mice’s condition and survival time recorded. Upon the death of the mice, HCC tissues were collected for further analysis. **I** Representative. **J** Survival curve. **K–M** Effects of *Traf3ip1*-cKO and *Rnaseh2c*-cKI on subcutaneous tumor growth (*n* = 6). **K** Representative. **L** Tumor growth curve. **M** Weight of the tumor. **N**, **O** Effects of *Traf3ip1*-cKO and *Rnaseh2c*-cKI on primary carcinoma growth (*n* = 6). **N** Representative. **O** Survival curve. **A–C**, **E**, **G**, **H** represented mean ± SD analyzed by Wilcoxon test, **D**, **L**, **M** represented mean ± SD analyzed by unpaired *t* test, **J**, **O** were analyzed by log-rank test. *P < 0.05, **P < 0.01. cKO conditional knockout, HCC hepatocellular carcinoma.

We conducted transcriptome sequencing to identify mTOR pathway-related genes regulated by *Rnaseh2c-*cKO and found that only *Traf3ip1* [17] was inhibited in mouse HCC-infiltrating macrophages (Fig. 2A). Enrichment analysis suggested *Rnaseh2c*-cKO was linked to Kyoto Encyclopedia of Genes and Genomes (KEGG) [18] terms “Antigen processing and presentation”, “Endocytosis”, and “Lysosome” (Fig. S1C). Given that the mTOR pathway suppresses lysosomal activity [19], we hypothesized that RNASEH2C promotes the lysosomal degradation of a specific protein via up-regulating the mTOR pathway-associated protein TRAF3IP1 expression, thereby affecting antigen presentation. What’s more, MHC II molecules are recognized for their role in activating the adaptive immune response during macrophage antigen presentation by displaying processed exogenous antigen peptides to Th1 cells [20], so we selected the internalization of MHC II molecules as the marker for evaluating macrophage antigen presentation. We conditionally knocked out or knocked in *Rnaseh2c* or *Traf3ip1* in macrophages and subsequently assessed their impact on the internalization of MHC II molecules by mouse HCC-infiltrating macrophages. Our findings indicated that *Rnaseh2c* or *Traf3ip1*-cKO enhanced the internalization of MHC II molecules in macrophages, whereas *Rnaseh2c* or *Traf3ip1*-cKI had the opposite effect (Fig. S1D, S1E). Furthermore, the mTOR pathway inhibitor Rapamycin attenuated the pro-internalization of *Rnaseh2c*-cKO (Fig. S1F). Consequently, we proposed that RNASEH2C in macrophages might inhibit the internalization of MHC II molecules by enhancing the expression of TRAF3IP1. Clarifying the role of RNASEH2C in tumor cells was also of critical importance. Our investigation into the effects of RNASEH2C on HCC cells revealed that neither knockout nor overexpression of *Rnaseh2c* in Hep-53.4 cells influenced tumor growth (Fig. S1G-S1I). Therefore, we focused on analyzing the tumor-promoting effect of RNASEH2C in macrophages.

We then explored whether the RNASEH2C-TRAF3IP1 axis suppressed macrophage antigen presentation to promote HCC growth. Our findings showed that *Rnaseh2c*-cKO reduced TRAF3IP1 transcription and protein expression in mouse HCC-infiltrating macrophages (Fig. 2B, C), while *Rnaseh2c*-cKI increased them (Fig. 2B, D). Subsequently, we investigated the mechanisms by which RNASEH2C enhances the transcription of *Traf3ip1*. The protein encoded by the *Rnaseh2c* gene is a subunit of the RNASEH2 complex, whose primary function is to identify and excise RNA from RNA/DNA hybrids [21]. This RNA is typically generated during DNA replication and forms part of the R-loop structure [22]. R-loops can either inhibit transcription initiation by obstructing the binding of transcription factors or facilitate transcription by enhancing the binding of transcription factors or preventing the binding of transcriptional repressors [23]. Analysis of DNA-RNA immunoprecipitation (DRIP)-seq signal peak data along with Cleavage Under Targets and Tagmentation (CUT&Tag) signal peak data obtained using an anti-S9.6 antibody from the UCSC database [24], revealed the presence of the R-loop structure on the human TRAF3IP1 promoter (Fig. S2A). Therefore, we speculated that there is also the R-loop structure on the promoter of mouse *Traf3ip1*. The S9.6 antibody is known to recognize RNA/DNA hybrids in a sequence-independent manner [25], making it a valuable tool for assessing R-loop formation in DRIP experiments [26]. In this study, we employed the DRIP-qPCR to assess whether RNASEH2C could mitigate R-loop levels on the promoter of mouse *Traf3ip1* (Fig. S2B). Specifically, we utilized the pFC53 plasmid containing a 1.2 kb DNA sequence from the CpG island of mouse gene *Airn* to construct the R-loops for in vitro transcription assays [27]. Following the overexpression of *Rnaseh2c*, a reduction in the R-loop levels generated by pFC53 was observed (Fig. S2C). This finding suggested that RNASEH2C could diminish R-loop formation in vitro. It has been documented that R-loops can impede RNA pol II elongation and gene transcription [28]. For example, TDRD3 enhances human c-Myc gene expression by reducing R-loop formation on its promoter [29]. Consequently, we hypothesized that RNASEH2C might similarly promote RNA pol II elongation and gene expression by disrupting R-loops on the promoter of mouse c-*Myc*. To test this hypothesis, we introduced *Rnaseh2c* into an in vitro transcription reaction and observed a reduction in R-loop levels on the mouse *c-Myc* promoter, akin to the effect of *E. coli Topo1* (Fig. S2D). Analogously, we proposed that RNASEH2C might also suppress transcription on the mouse *Traf3ip1* promoter region by inhibiting R-loop formation. Our investigation revealed that the promoter of *Traf3ip1* was capable of effectively forming the R-loops during in vitro transcription, but not during reverse transcription (Fig. S2E). The addition of *Rnaseh2c* to the in vitro transcription reaction was observed to decrease the R-loop levels on the *Traf3ip1* promoter, exhibiting a similar effect to that of *E. coli Topo1* (Fig. S2F). Furthermore, RNase H was found to attenuate the enhancement effect of *Rnaseh2c*-cKO on DRIP signaling on the *Traf3ip1* promoter and transcript expression of *Traf3ip1* (Fig. S2G, S2H). Collectively, these findings suggested that RNASEH2C augmented the transcriptional activity of mouse *Traf3ip1* by inhibiting R-loop formation on its promoter.

Next, we analyzed the effect of TRAF3IP1 deficiency on macrophage polarization and found that, like RNASEH2C, it did not participate in regulating macrophage polarization (Fig. S2I). Therefore, we then investigated whether TRAF3IP1 is the key for RNASEH2C to inhibit the mTOR pathway and thereby promote macrophage-dependent HCC growth. Rescue experiments indicated *Rnaseh2c*-KO enhanced phosphorylation of mTOR, S6, and S6K in mouse HCC-infiltrating macrophages, whereas *Traf3ip1*-cKI diminished this positive effect (Fig. 2E), suggesting RNASEH2C inhibited the mTOR pathway by promoting TRAF3IP1 expression. Furthermore, animal studies indicated that *Traf3ip1*-cKO suppressed tumor growth and extended survival (Fig. 2F–J). However, *Rnaseh2c*-cKI reduced *Traf3ip1*-cKO’s antitumor effect (Fig. 2K-O). Thus, we suggested that RNASEH2C enhanced tumor progression by increasing TRAF3IP1 expression and inhibiting the mTOR pathway.

### RNASEH2C promoted tumor growth by inhibiting RAI14 expression

**Fig. 3:**
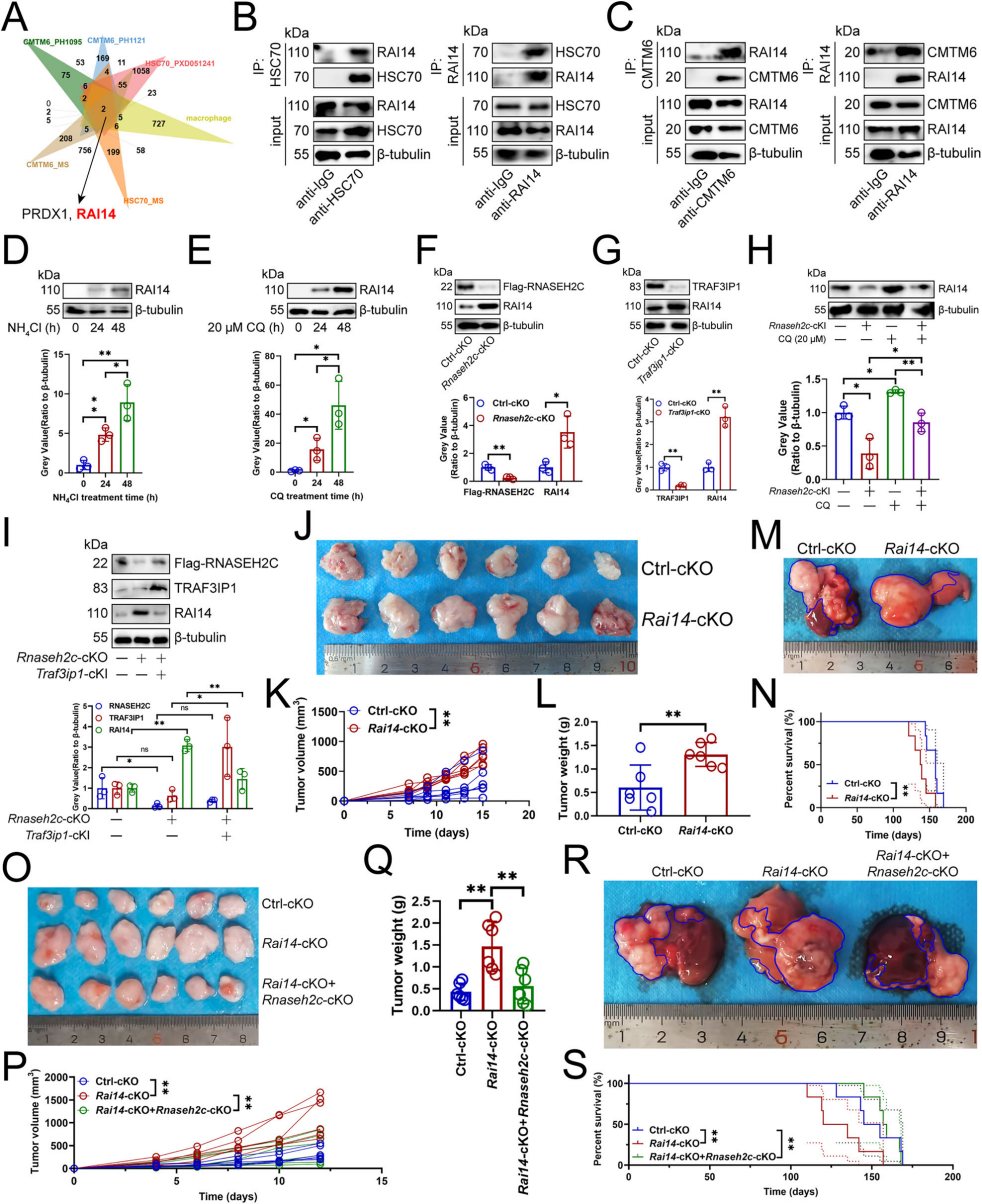
RNASEH2C promoted tumor growth by inhibiting RAI14 expression. **A** Immunocoprecipitation-mass spectrometry detecting the binding of macrophage-associated proteins to HSC70 or CMTM6. The cell lysate derived from mouse HCC-infiltrating macrophages was co-incubated with either an anti-HSC70 antibody or an anti-CMTM6 antibody. The proteins precipitated by these antibodies were subsequently analyzed via mass spectrometry. **B** Mouse HCC-infiltrating macrophage lysate was treated with an anti-IgG control antibody and an anti-HSC70 antibody (left) or an anti-RAI14 antibody (right), and 5% lysate was used as input control (*n* = 3). **C** Mouse HCC-infiltrating macrophage lysate was treated with an anti-IgG control antibody and an anti-CMTM6 antibody (left) or an anti-RAI14 antibody (right) (*n* = 3). Effects of NH_4_Cl (**D**) and CQ (**E**) on the expression of RAI14 protein in mouse HCC-infiltrating macrophages (*n* = 3). Macrophages were exposed to 0.3 M NH_4_Cl and 20 μM CQ for a predetermined duration, and the protein expression levels of RAI14 were assessed through immunoblotting. **F**, **G** Immunoblotting analyzing the effect of *Rnaseh2c-cKO* or *Traf3ip1*-cKO on the expression of RAI14 protein in mouse HCC-infiltrating macrophages (*n* = 3). **H** Immunoblotting analyzing the effect of *Rnaseh2c*-cKI and CQ treatment on the expression of RAI14 protein in mouse HCC-infiltrating macrophages (*n* = 3). Both wild-type and *Rai14*-cKO macrophages underwent treatment with 20 μM CQ for 48 h, followed by the detection of RAI14 expression via immunoblotting. **I** Effect of *Rnaseh2c*-cKO and *Traf3ip1*-cKI on the expression of RAI14 protein in mouse HCC-infiltrating macrophages (*n* = 3). **J–L** Effects of *Rai14*-cKO on subcutaneous tumor growth (*n* = 6). Wild-type Hep-53.4 cells were implanted into the left axillary region of mice. Tumor volume was measured regularly, and mice were euthanized when the diameter of any tumor exceeded 2 cm; the tumors were then excised and weighed. **J** Representative. **K** Tumor growth curve. **L** Weight of the tumor. **M**, **N** Effects of *Rai14*-cKO on primary carcinoma growth (*n* = 6). DEN and CCl_4_ were infused into mice to construct primary cancers, with observations made on the mice’s condition and survival time recorded. Upon the death of the mice, HCC tissues were collected for further analysis. **M** Representative. **N** Survival curve. **O–Q** Effects of *Rai14-cKO* and *Rnaseh2c*-cKO on subcutaneous tumor growth (*n* = 6). **O** Representative. **P** Tumor growth curve. **Q** Weight of the tumor. **R**, **S** Effects of *Rai14-cKO* and *Rnaseh2c*-cKO on primary carcinoma growth (*n* = 6). **R** Representative. **S** Survival curve. **D**, **F**, **G**, **I**, **K**, **L** represented mean ± SD analyzed by unpaired *t* test, **E**, **H**, **P**, **Q** represented mean ± SD analyzed by Wilcoxon test, **N**, **S** were analyzed by log-rank test. *P < 0.05, **P < 0.01. cKI conditional knock in, cKO conditional knockout, CQ chloroquine phosphate, HCC hepatocellular carcinoma.

Given that mTOR pathway inhibits lysosomal degradation [19] and *Rnaseh2c*-cKO was linked to KEGG term “Lysosome” (Fig. S1C), while *Rnaseh2c*-cKO inhibited *Traf3ip1* transcriptionally (Fig. 2A–D, S2), we proposed that the RNASEH2C-TRAF3IP1 axis enhances lysosomal degradation of a specific protein to inhibit macrophage antigen presentation in HCC. We investigated macrophage-associated genes [30] interacting with HSC70 and CMTM6, which regulate lysosomal circulation [6, 31]. Our data and public datasets [31, 32] showed that the macrophage antigen-presenting gene RAI14 [33] bound to HSC70 and CMTM6 (Fig. 3A). Immunoprecipitation confirmed RAI14’s binding to both proteins in mouse HCC-infiltrating macrophages (Fig. 3B, C). Furthermore, lysosome inhibitors NH_4_Cl and CQ increased RAI14 protein expression in macrophages (Fig. 3D, E), indicating its lysosomal degradation. To elucidate the role of the RNASEH2C-TRAF3IP1 axis in the regulation of RAI14, we measured the levels of RAI14 after *Rnaseh2c* or *Traf3ip1*-cKO or -cKI. Notably, *Rnaseh2c*-cKO and *Traf3ip1*-cKO boosted RAI14 protein levels without altering its transcripts in macrophages (Figs. 3F, G, S3A). However, *Rnaseh2c* or *Traf3ip1*-cKI inhibited RAI14 protein levels without altering its transcripts in macrophages (Fig. S3A–S3C). To assess RNASEH2C’s role in RAI14 lysosomal degradation, we treated *Rnaseh2c*-cKI macrophages with CQ. It was observed that *Rnaseh2c*-cKI resulted in a reduction of RAI14 protein expression, while concurrently, CQ enhanced its stability (Fig. 3H). Rescue experiment indicated that *Rnaseh2c*-cKO enhanced RAI14 protein expression in macrophages, while *Traf3ip1*-cKI reduced this positive effect (Fig. 3I), suggesting that the RNASEH2C-TRAF3IP1 axis facilitated RAI14’s lysosomal degradation. Animal studies revealed that *Rai14*-cKO accelerated tumor growth and reduced survival (Fig. 3J–N), but this effect was mitigated by *Rnaseh2c*-cKO (Fig. 3O–S). Therefore, we suggested that RNASEH2C induced lysosomal degradation of RAI14 by promoting TRAF3IP1 expression.

Subsequently, we investigated the role of RAI14 in the polarization of macrophages and assessed whether its tumor-regulating effects are exclusively dependent on immune mechanisms. Our findings indicated that *Rai14*-cKO did not influence the expression of the four polarization markers: CD80, CD86, CD163, and ARG1 (Fig. S3D). The deletion of *Rai14* in Hep-53.4 cells resulted in the suppression of subcutaneous tumor growth, whereas its overexpression produced the opposite effect (Fig. S3E–S3J). Furthermore, in situ transplantation of Hep-53.4 cells with *Rai14* knockout into the liver led to inhibited tumor growth and extended survival, with the inverse observed upon overexpression (Fig. S3K–S3P). The findings suggested that the tumor-regulatory function of RAI14 was contingent upon the specific cell type. In tumor cells, RAI14 demonstrated a pro-tumorigenic effect, whereas in macrophages, it exhibited an anti-tumorigenic effect. Consequently, the utilization of *Rai14*-cKO mice was imperative to assess its immunomodulatory properties.

### HSC70 promoted the lysosomal degradation of RAI14

**Fig. 4:**
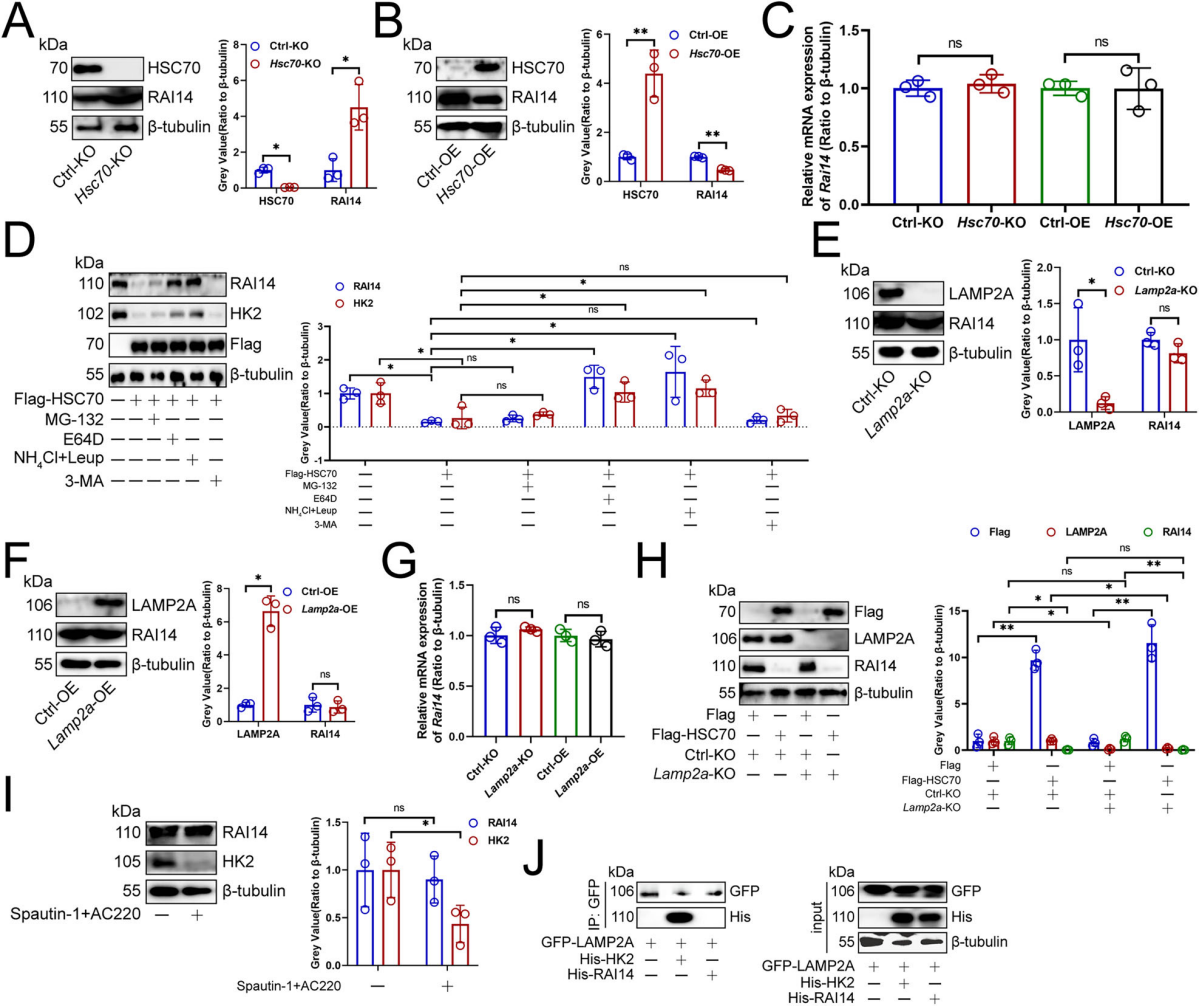
HSC70 promoted the lysosomal degradation of RAI14. Immunoblotting demonstrating the effect of knockout (**A**) and overexpression (**B**) of *Hsc70* on RAI14 protein expression in RAW264.7 cells (*n* = 3). **C** qPCR showing the effect of knockout and overexpression of *Hsc70* on the expression of *Rai14* transcripts in RAW264.7 cells (*n* = 3). **D** Immunoblotting analyzing the expression of RAI14, HK2, and Flag proteins in RAW264.7 cells (*n* = 3). RAW264.7 cells were treated with 10 mM MG-132, 10 μM E64D, 0.3 M NH_4_Cl, 100 nM Leupeptin, and 5 mM 3-MA for 48 h. Subsequently, the protein expressions of RAI14, HK2, and Flag-labeled HSC70 were analyzed via immunoblotting. Effect of knockout (**E**) or overexpression (**F**) of *Lamp2a* on RAI14 protein expression in RAW264.7 cells (*n* = 3). **G** qPCR exploring the effect of knockout and overexpression of *Lamp2a* on the expression of *Rai14* transcripts in RAW264.7 cells (*n* = 3). **H** Immunoblotting analysis of the expression of Flag, LAMP2A, and RAI14 proteins in RAW264.7 cells (*n* = 3). *Lamp2a* was knocked out and Flag-labeled HSC70 was transfected. **I** Immunoblotting demonstrating the effects of Spautin-1 and AC220 on the expression of RAI14 and HK2 proteins in mouse HCC-infiltrating macrophages (*n* = 3). **J** Immunoprecipitation assessing the interaction between RAI14 or HK2 and LAMP2A in mouse HCC-infiltrating macrophages (*n* = 3). **A**, **C**, **D**, **F**, **H** represented mean ± SD analyzed by Wilcoxon test, **B**, **E**, **G**, **I** represented mean ± SD analyzed by unpaired *t* test. *P < 0.05, **P < 0.01. HCC hepatocellular carcinoma.

Given the significant role of HSC70 and CMTM6 in regulating lysosomal degradation [6, 7], we next explored whether they affect the protein expression of RAI14 in macrophages. We manipulated HSC70 levels and analyzed the expression of RAI14 transcript and protein. We found that knocking out *Hsc70* increased RAI14 protein levels in RAW264.7 cells (Fig. 4A), while overexpression decreased them (Fig. 4B), without affecting its transcription levels (Fig. 4C), indicating HSC70’s influence at its protein levels. To determine whether RNASEH2C must rely on HSC70 or CMTM6 to inhibit RAI14 expression, we overexpressed *Rnaseh2c* and knocked out *Hsc70* or knocked out *Rnaseh2c* and *Cmtm6* and found that *Rnaseh2c*-OE inhibited RAI14 protein levels in RAW264.7 cells, while *Hsc70*-KO counteracted RNASEH2C’s inhibition (Fig. S3Q). Moreover, *Rnaseh2c*-KO increased RAI14 protein levels in RAW264.7 cells, but *Cmtm6*-KO weakened *Rnaseh2c*-cKO’s positive effect (Fig. S3R). However, our findings indicated that neither RNASEH2C nor TRAF3IP1 exerted any significant influence on the protein expression levels of HSC70 or CMTM6 in mouse HCC-infiltrating macrophages (Fig. S3S–S3U). Thus, RNASEH2C advanced tumor progression by promoting RAI14 lysosomal degradation, with HSC70 and CMTM6 playing key roles.

To further verify that HSC70-induced RAI14 downregulation relies on lysosomal protein degradation, we treated *Hsc70*-OE cells with proteasome (MG-132), lysosome (E64D or Leupeptin+NH_4_Cl), or autophagy (3-MA) inhibitors. Our findings indicated that, similar to HK2, which is subject to lysosomal degradation [34], the expression of RAI14 protein in RAW264.7 cells was restored exclusively through lysosomal inhibition (Fig. 4D). Chaperone-mediated autophagy (CMA) is a process that transports proteins into lysosomes for degradation [35]. Then, our research focus was on the role of CMA in the degradation of RAI14. The CMA process involves several key steps: (1) HSC70 recognizes and binds substrates containing KFERQ motifs; (2) the substrate-chaperone complex associates with LAMP2A; (3) the substrate is unfolded, forming a CMA translocation complex; (4) HSC70 facilitates substrate translocation; (5) lysosomal proteases degrade the substrate; and (6) LAMP2A dissociates from the translocation complex, which is regarded as the rate-limiting component of CMA [35]. However, neither overexpression nor knockout of *Lamp2a* influenced the protein or transcript levels of RAI14 in RAW264.7 cells (Fig. 4E–G). Unsurprisingly, the protein expression of RAI14 was inhibited by *Hsc70*-OE in RAW264.7 cells, while the inhibitory effect of HSC70 was not attenuated by *Lamp2a*-KO (Fig. 4H). Furthermore, treatments with Spautin-1 and AC220, which are established inducers of CMA through the degradation of the key negative regulator HK2 [36], did not affect RAI14 protein expression (Fig. 4I). Furthermore, our findings revealed that although RAI14 interacted with HSC70 (Fig. 3A, B), it lacked the KFERQ motif and was unable to bind to LAMP2A, unlike the positive control HK2 [34] (Fig. 4J). These findings indicated that the degradation of RAI14 induced by HSC70 was lysosome-dependent yet independent of CMA.

### HSC70 promoted the degradation of RAI14 through the endosome and lysosomal pathways

**Fig. 5:**
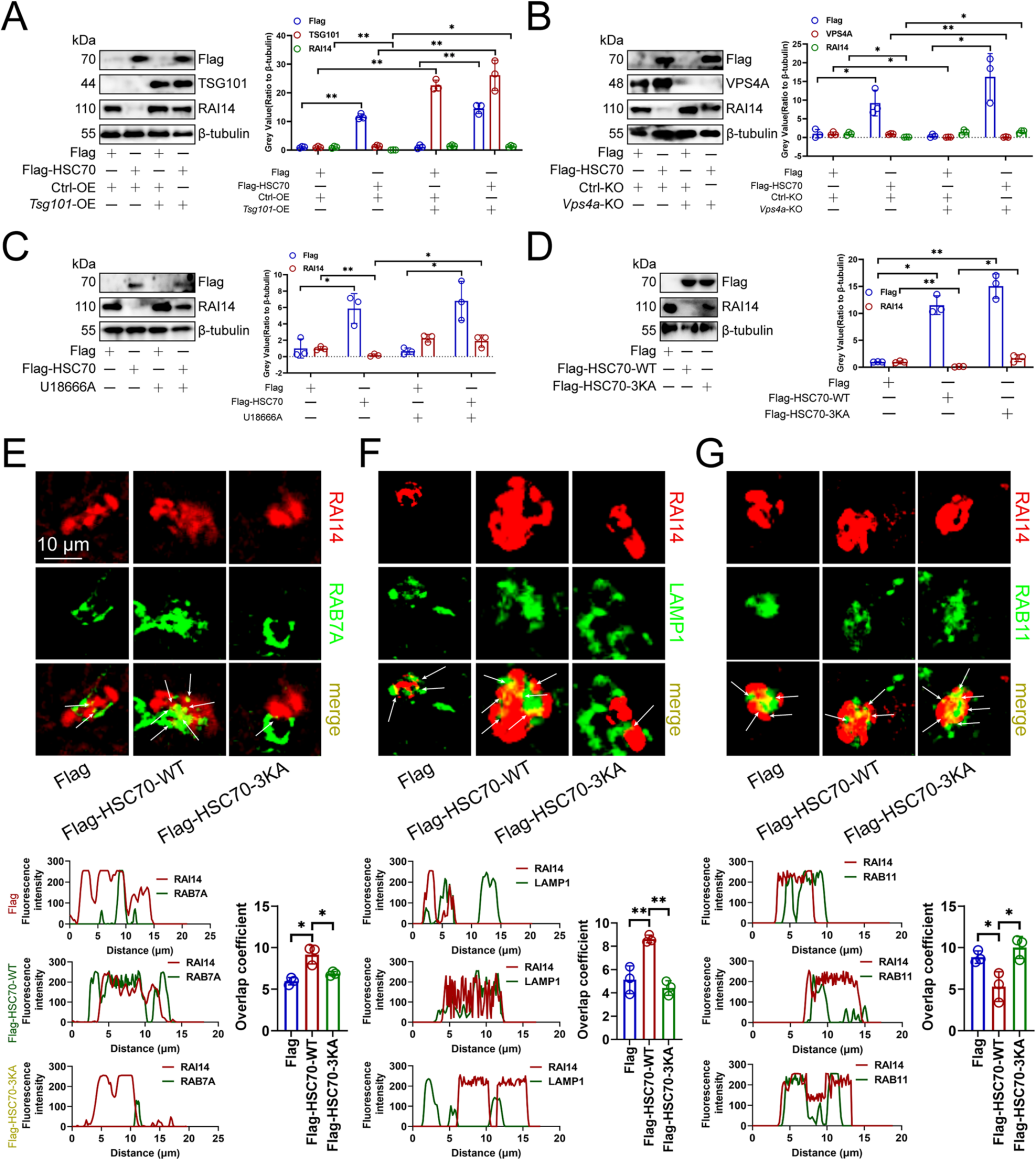
HSC70 promoted the degradation of RAI14 through the endosome and lysosomal pathways. **A** Immunoblotting showing the expression of Flag, TSG101. and RAI14 proteins in RAW264.7 cells (*n* = 3). *Tsg101* was overexpressed and Flag-labeled HSC70 was transfected. **B** Immunoblotting analyzing the expression of Flag, VPS4A, and RAI14 proteins in RAW264.7 cells (*n* = 3). *Vps4a* was knocked out and Flag-labeled HSC70 was transfected. **C** Immunoblotting demonstrating the expression of Flag and RAI14 proteins in RAW264.7 cells (*n* = 3). Cells were treated with 5 μg/mL U18666A and transfected with Flag-labeled HSC70. **D** Immunoblotting exploring the expression of Flag and RAI14 proteins in RAW264.7 cells (*n* = 3). Cells were transfected with Flag-labeled HSC70-WT or HSC70-3KA. Immunofluorescence analysis of the co-localization of RAB7A (**E**), LAMP1 (**F**) or RAB11 (**G**) with RAI14 in RAW264.7 cells (*n* = 3). Cells were transfected with Flag-labeled HSC70-WT or HSC70-3KA. **A–E** Represented mean ± SD analyzed by Wilcoxon test, **F**, **G** represented mean ± SD analyzed by unpaired *t* test. *P < 0.05, **P < 0.01.

HSC70 has been identified as a pivotal chaperone protein within CMA and endosomal microautophagy (eMI) pathways, which is a key activator and executor of eMI (especially a form that intersects with CMA) [37]. Consequently, we investigated whether RAI14 undergoes degradation via the eMI pathway. The ESCRT-I complex component TSG101 is crucial for the formation of multivesicular bodies (MVBs) and facilitates the transport of cytoplasmic proteins to endosomes, which is a key inhibitory factor of eMI [38]. VPS4, another critical element of the eMI pathway, functions as the regulatory ATPase of ESCRT-IIII, which is a key activator necessary for eMI execution [39]. To investigate the role of essential eMI regulators in the HSC70-mediated degradation of RAI14, we conducted experiments involving the overexpression of *Tsg101* and the knockout of *Vps4*. Our findings demonstrated that the overexpression of *Tsg101* or the knockout of *Vps4* impeded the HSC70-induced degradation of the RAI14 protein in RAW264.7 cells (Fig. 5A, B). Furthermore, it has been documented that the cholesterol transport inhibitor U18666A impedes MVB dynamics, thereby inhibiting eMI [38]. Consistently, U18666A also obstructed the degradation of RAI14 protein in RAW264.7 cells (Fig. 5C). These observations implied that the HSC70-mediated degradation of RAI14 was reliant on the eMI pathway. It has been reported that the interaction between the carboxy-terminal lid domain of HSC70 and phosphatidylserine on the late endosomal membrane is critical for eMI [40]. The HSC70-3KA mutant, characterized by alterations in the C-terminal lysine residues, disrupts this binding to phosphatidylserine [38]. We found that HSC70-3KA did not lead to a reduction in RAI14 compared to wild-type protein (HSC70-WT) in RAW264.7 cells (Fig. 5D). Consistently, HSC70-WT increased co-localization of RAI14 with late endosome RAB7A and lysosome LAMP1 in RAW264.7 cells, while decreasing co-localization of RAI14 with circulating endosome RAB11 (Fig. 5E–G). However, these effects were not observed when HSC70-3KA was transfected (Fig. 5E–G). These findings supported that HSC70 promoted RAI14 degradation through the endosome and lysosomal pathways.

### HSC70 competed with CMTM6 to bind and regulate RAI14 expression

**Fig. 6:**
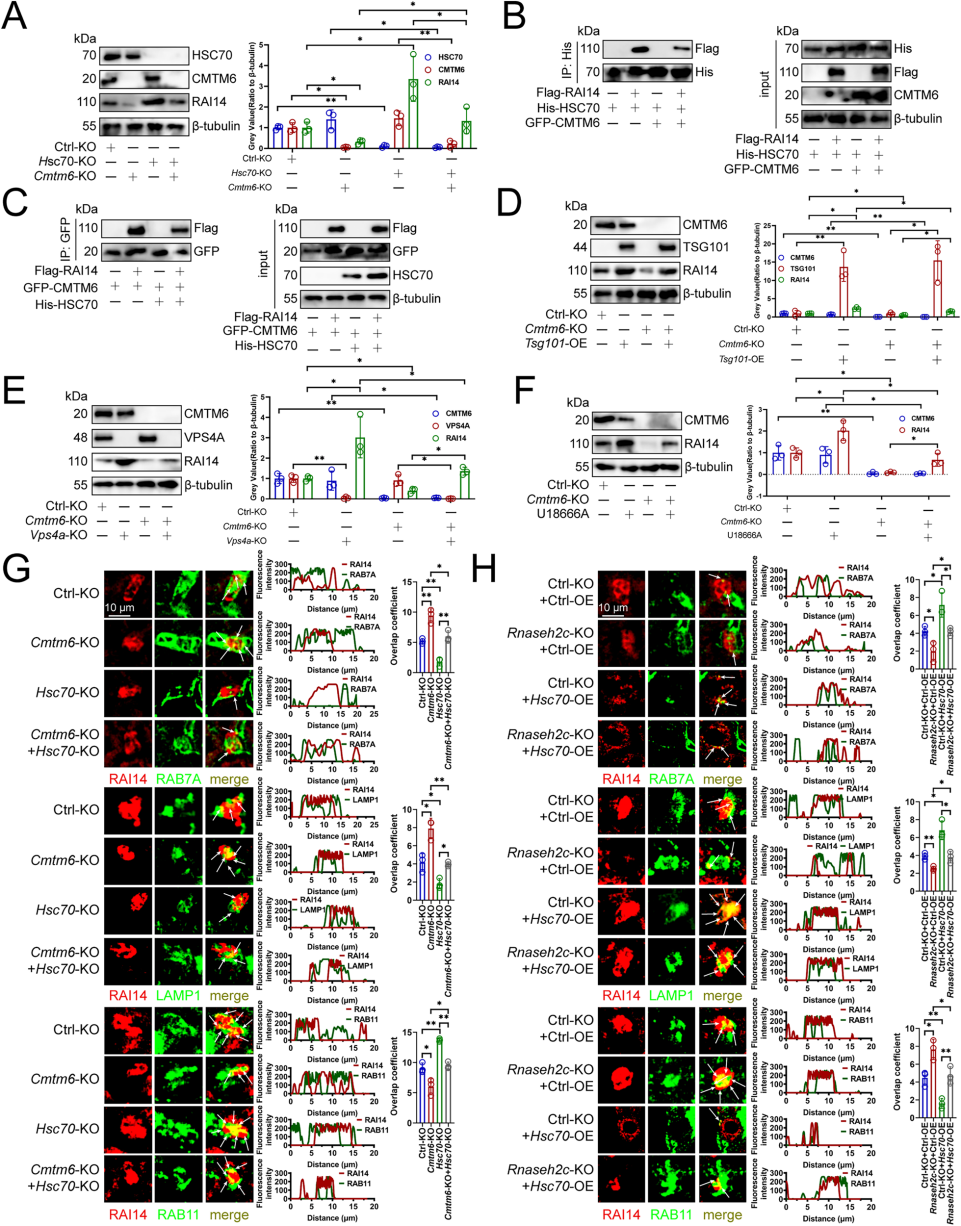
HSC70 competed with CMTM6 to bind and regulate RAI14 expression. **A** Immunoblotting showing the expression of HSC70, CMTM6 and RAI14 proteins in RAW264.7 cells (*n* = 3). *Hsc70* or *Cmtm6* was knockout. **B** Immunoprecipitation detecting the effect of overexpression of *Cmtm6* on the binding of HSC70 and RAI14 in RAW264.7 cells (*n* = 3). **C** Immunoprecipitation analyzing the effect of overexpression of *Hsc70* on the binding of CMTM6 and RAI14 in RAW264.7 cells (*n* = 3). **D** Immunoblotting analyzing the protein expression of CMTM6, TSG101, and RAI14 in RAW264.7 cells (*n* = 3). *Cmtm6* was knockout or *Tsg101* was overexpressed. **E** Immunoblotting exploring the protein expression of CMTM6, VPS4A, and RAI14 in RAW264.7 cells (*n* = 3). *Cmtm6* or *Vps4a* was knockout. **F** Immunoblotting detecting the expression of CMTM6 and RAI14 proteins in RAW264.7 cells (*n* = 3). Cells were treated with 5 μg/mL U18666A and *Cmtm6* was knocked out. **G**, **H** Immunofluorescence observing the co-localization of RAB7A, LAMP1 or RAB11 with RAI14 in RAW264.7 cells (*n* = 3). **G** *Hsc70* or *Cmtm6* was knockout. **H** *Rnaseh2c* was knockout and *Hsc70* was overexpressed. **A**, **D–H** Represented mean ± SD analyzed by Wilcoxon test. *P < 0.05, **P < 0.01.

CMTM6 has been reported to inhibit PD-L1 degradation via lysosomes [31], prompting us to explore its interaction with HSC70 on RAI14 protein expression. Overexpressing or knocking out *Hsc70* did not affect CMTM6 protein or transcript levels in RAW264.7 cells (Fig. S4A–S4C). Moreover, *Cmtm6*-KO resulted in the suppression of RAI14 protein expression, while it did not influence the expression of HSC70 protein in RAW264.7 cells (Fig. S4D). Conversely, *Cmtm6*-OE promoted the expression of RAI14 protein without altering HSC70 protein expression in RAW264.7 cells (Fig. S4E). Furthermore, alterations in CMTM6 expression, whether through knockout or overexpression, did not impact the transcriptional levels of *Rai14* or *Hsc70* in RAW264.7 cells (Fig. S4F). Rescue experiment indicated knocking out *Cmtm6* reduced RAI14 protein expression, but knocking out *Hsc70* restored it in RAW264.7 cells (Fig. 6A), indicating CMTM6-mediated RAI14 degradation depended on HSC70.

Immunoprecipitation assays demonstrated a diminished interaction between RAI14 and HSC70 in the presence of *Cmtm6* overexpression, as well as a reduced interaction between RAI14 and CMTM6 when *Hsc70* was overexpressed, indicating the possibility of competitive binding (Fig. 6B, C). To further investigate this interaction, we engineered a series of truncated RAI14 proteins (Fig. S4G). Molecular docking predicted the binding of HSC70 or CMTM6 with RAI14 (Fig. S4H, S4I). Immunoprecipitation indicated that the ANK and Coiled coil domains of RAI14 bound to HSC70, and the ANK, Nuclear localization signal, and Coiled coil domains of RAI14 bound to CMTM6 (Fig. S4J, S4K). In turn, we constructed a series of truncated sequences of HSC70 or CMTM6 (Fig. S5A, S5B). The HSC70 domain of HSC70 or the NTR and MARVEL-1 domains of CMTM6 were combined with RAI14 (Fig. S5C, S5D). These results indicated that HSC70 or CMTM6 competitively combined with RAI14.

To evaluate the impact of competitive binding between HSC70 and CMTM6, we examined if *Cmtm6*-KO-induced degradation of RAI14 relies on eMI [38, 39]. We found that overexpressing *Tsg101* or knocking out *Vps4* [38, 39], restored RAI14 expression in *Cmtm6*-KO cells (Fig. 6D, E). U18666A [38] also prevented RAI14 degradation in the absence of CMTM6 (Fig. 6F). What’s more, immunofluorescence showed that *Cmtm6*-KO increased RAI14’s interaction with RAB7A in RAW264.7 cells, but this was disrupted by simultaneous knockout of *Cmtm6* and *Hsc70* (Fig. 6G). Similar patterns were seen with RAI14 and LAMP1 in RAW264.7 cells (Fig. 6G). *Cmtm6*-KO reduced RAI14’s co-localization with RAB11 in RAW264.7 cells, whereas *Hsc70*-KO weakened this negative effect (Fig. 6G). Additionally, *Rnaseh2c*-KO weakened RAI14’s interaction with RAB7A in RAW264.7 cells, but this was enhanced by concurrent *Rnaseh2c*-KO and *Hsc70*-OE, with similar results for RAI14 and LAMP1 co-localization (Fig. 6H). Furthermore, knocking out *Rnaseh2c* enhanced RAI14 and RAB11 co-localization in RAW264.7 cells, whereas *Hsc70*-OE reduced it (Fig. 6H). Overall, these results indicated that CMTM6 and HSC70 competed to bind RAI14, with *Cmtm6*-KO boosting HSC70-RAI14 interaction and facilitating RAI14 degradation via eMI.

### RNASEH2C-TRAF3IP1-RAI14 axis facilitated macrophage-mediated antigen presentation

**Fig. 7:**
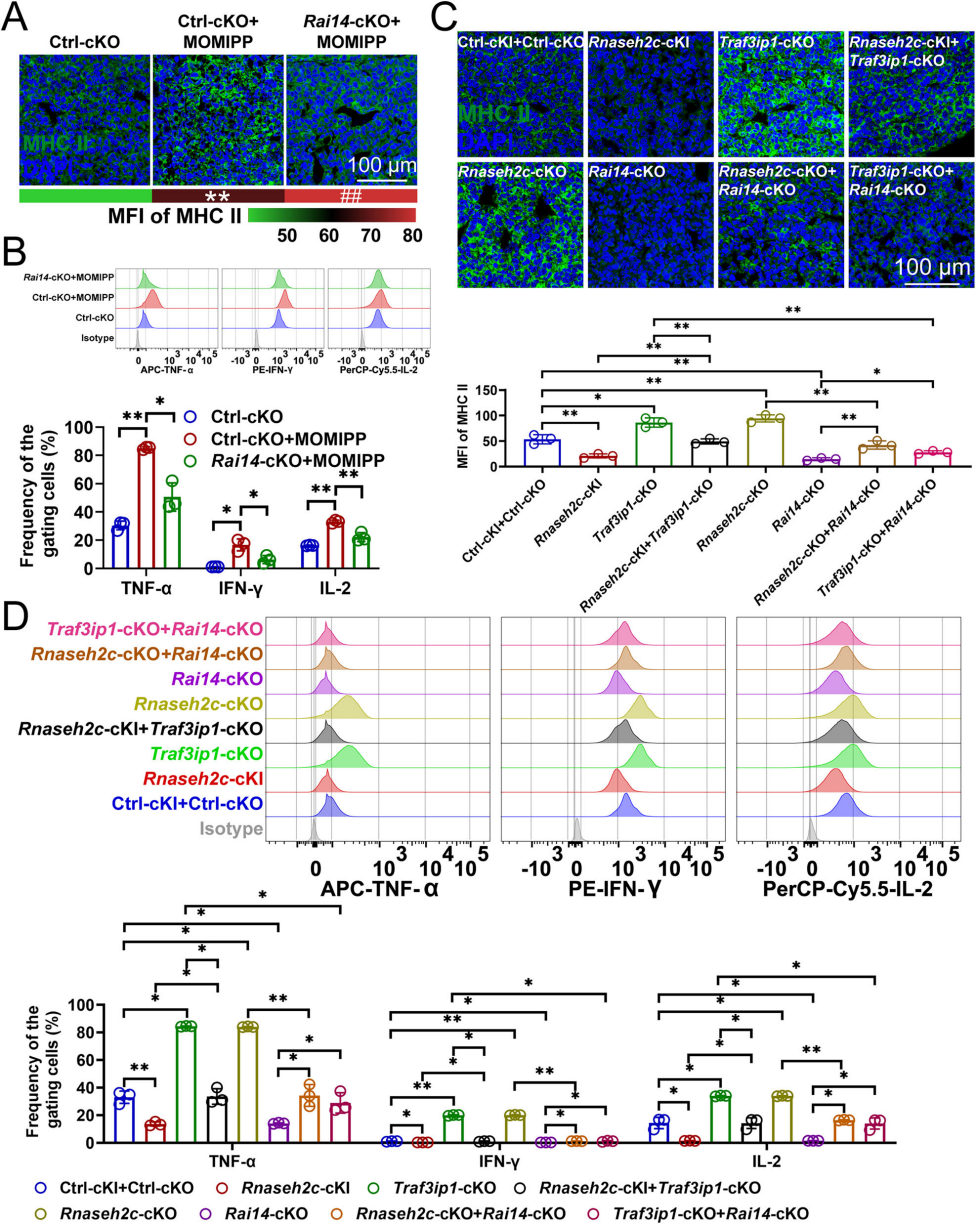
RNASEH2C-TRAF3IP1-RAI14 axis facilitated macrophage-mediated antigen presentation. **A** Immunofluorescence assessing the impact of MOMIPP or *Rai14*-cKO on the internalization of MHC II molecules by mouse HCC-infiltrating macrophages (*n* = 3). Mice exhibiting primary HCC were stimulated with MOMIPP. Subsequently, macrophages were isolated from tumor tissues and immunized with the OVA_323-339_. The immunized macrophages were then co-cultured with OT-II T cells derived from OT-II mice for 3 d. Following this co-culture period, MHC II molecules in the macrophages were analyzed using immunofluorescence. **, Ctrl-cKO+MOMIPP vs Ctrl-cKO; ##, *Rai14*-cKO+MOMIPP vs Ctrl-cKO+MOMIPP. **B** Flow cytometry evaluating the influence of MOMIPP or *Rai14*-cKO on the protein expression of TNF-α, IFN-γ, or IL-2 in CD4^+^ T cells (*n* = 3). Mice exhibiting primary HCC were stimulated with MOMIPP. Subsequently, macrophages were isolated from tumor tissues and immunized with the OVA_323-339_. The immunized macrophages were then co-cultured with OT-II T cells derived from OT-II mice for 3 d. Following this co-culture period, the cytokine expression in CD4^+^ T cells was assessed through flow cytometry. **C** Immunofluorescence investigating the effects of various genetic manipulations, including *Rnaseh2c*-cKI, *Traf3ip1*-cKO, *Rnaseh2c*-cKI+*Traf3ip1*-cKO, *Rnaseh2c*-cKO, *Rai14*-cKO, *Rnaseh2c*-cKO+*Rai14*-cKO, as well as *Traf3ip1*-cKO+*Rai14*-cKO, on the internalization of MHC II molecules by mouse HCC-infiltrating macrophages (*n* = 3). **D** Flow cytometry analyzing the effects of various genetic manipulations, including *Rnaseh2c*-cKI, *Traf3ip1*-cKO, *Rnaseh2c*-cKI+*Traf3ip1*-cKO, *Rnaseh2c*-cKO, *Rai14*-cKO, *Rnaseh2c*-cKO+*Rai14*-cKO, as well as *Traf3ip1*-cKO+*Rai14*-cKO, on the protein expression of TNF-α, IFN-γ, or IL-2 in CD4^+^ T cells (*n* = 3). **A**, **C** represented mean ± SD analyzed by unpaired *t* test, **B**, **D** represented mean ± SD analyzed by Wilcoxon test. *P < 0.05, **P < 0.01, ^##^*P* < 0.01. cKI conditional knock in, cKO conditional knockout, HCC hepatocellular carcinoma.

We next examined the impact of macropinocytosis, sustained by RAI14, on macrophage-mediated antigen presentation. This process is characterized by several stages: uptake of MHC II molecules, intracellular trafficking of MHC II molecules, uptake and degradation of tumor-associated antigens, peptide loading, and the activation of CD4^+^ T cells [41]. The ovalbumin (OVA)_323-339_ serves as a model antigen in the investigation of antigen presentation facilitated by MHC II molecules [41]. Upon the presentation of OVA_323-339_ by macrophages, OT-II T cells (CD4^+^ T cells) underwent activation following immunization. Consequently, the OVA_323-339_ is extensively utilized as a model antigen to study adaptive immune responses, particularly focusing on the activation, proliferation, differentiation, and function of CD4^+^ T cells mediated by MHC II molecules on macrophages [41]. Our findings indicated that the macropinocytosis inducer, MOMIPP, enhanced the internalization of MHC II molecules in mouse HCC-infiltrating macrophages and promoted their localization within the endoplasmic reticulum (Calnexin), Golgi apparatus (GM130), endosomes (EEA1), and lysosomes (LAMP1) (Fig. 7A, S6A–S6D). Additionally, MOMIPP increased the efficiency of the OVA_323-339_ uptake (Fig. S6E, S6F), degradation (Fig. S6E, S6G), and peptide loading onto MHC II molecules (Fig. S6E, S6H), thereby activating Th1 cells (Fig. 7B, S6I), whereas *Rai14*-cKO appeared to inhibit these processes (Figs. 7A, B, S6A–S6I). We then analyzed whether the RNASEH2C-TRAF3IP1 axis inhibits MHC II molecules internalization in macrophages and Th1 cell activation through suppressing RAI14 expression. Rescue assays revealed that *Rnaseh2c*-cKI suppressed the internalization of MHC II molecules in mouse HCC-infiltrating macrophages and inhibited Th1 cell activation (Fig. 7C, D). In contrast, *Traf3ip1*-cKO enhanced macrophage antigen presentation and mitigated the inhibitory effects of *Rnaseh2c*-cKI (Fig. 7C, D). Additionally, *Rnaseh2c*-cKO also facilitated macrophage antigen presentation and Th1 cell activation (Fig. 7C, D). Moreover, *Rai14*-cKO not only inhibited the internalization of MHC II molecules by macrophages and the expression of TNF-α, IFN-γ, and IL-2 in CD4^+^ T cells, but it also attenuated the augmentative effects observed with *Rnaseh2c* or *Traf3ip1*-cKO (Fig. 7C, D). Therefore, RAI14 alleviated the inhibitory effects of RNASEH2C and TRAF3IP1 on macrophage antigen presentation.

### Inhibition of RNASEH2C in macrophages enhanced the effectiveness of immune checkpoint inhibitors

**Fig. 8:**
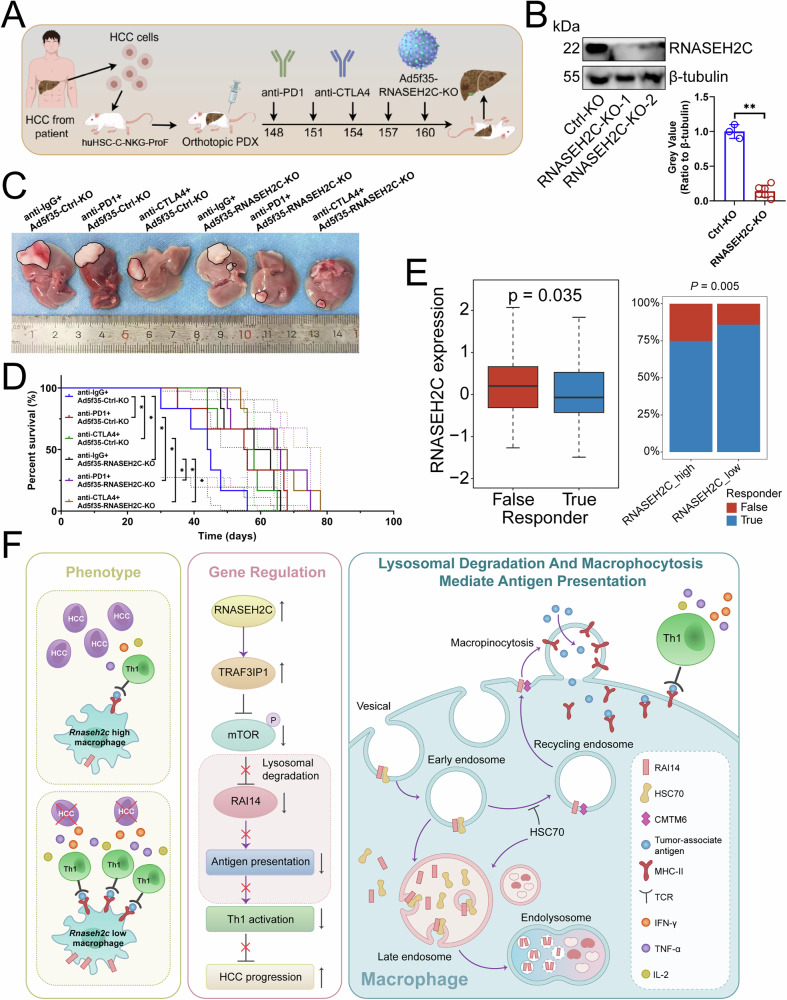
Inhibition of RNASEH2C in macrophages enhanced the effectiveness of immune checkpoint inhibitors. **A** Operation flow chart. **B** Immunoblotting showing the expression of RNASEH2C protein in human macrophages (*n* = 3, *n* = 6). To knock out RNASEH2C in human macrophages, Ad5f35 adeno-associated virus was employed, and these modified cells were then introduced into huHSC-C-NKG-ProF mice with humanized immune systems. **C**, **D** Effect of knockout of RNASEH2C on human macrophages and anti-PD1 or anti-CTLA4 antibodies on the progression of orthotopic carcinoma (*n* = 6). HCC tissues from patients were processed into single-cell suspensions and implanted into the livers of huHSC-C-NKG-ProF mice. Subsequently, either human macrophages or neutralizing antibodies were administered to the tumor-bearing mice. The health status of the mice was monitored, and their survival times were documented. Upon the death of the mice, HCC tissues were collected for further analysis. **C** Representative. **D** Survival curve. **E** Left: TIDE examining the mRNA expression of RNASEH2C in patients who demonstrated either responsiveness or non-responsiveness to immunotherapy (*n* = 371). Right: TIDE evaluating the sensitivity to immunotherapy in HCC patients exhibiting varying levels of RNASEH2C expression (*n* = 371). **F** Mechanism diagram. **B** Represented mean ± SD analyzed by unpaired *t* test, **D** was analyzed by log-rank test, **E** represented mean ± SD analyzed by Wilcoxon test. *P < 0.05, **P < 0.01. HCC hepatocellular carcinoma, TIDE tumor immune dysfunction and exclusion.

Finally, we explored the clinical impact of inhibiting RNASEH2C in human macrophages and its synergy with immune checkpoint inhibitors (Fig. 8A). In the patient-derived orthotopic xenograft (PDOX) model, the Ad5f35 adenovirus vector [42] carrying RNASEH2C-KO was employed to achieve knockout of RNASEH2C in human macrophages, ensuring an infection efficiency of no less than 50% (Fig. 8B, S7A). Subsequently, these macrophages were transfused into PDOX model, and anti-PD1 or anti-CTLA4 antibodies were administered via tail vein injection. Our findings indicated that either the knockout of RNASEH2C or the administration of neutralizing antibodies alone reduced tumor growth and improved survival rates (Fig. 8C, D). Notably, the combined application of both strategies resulted in a more pronounced effect (Fig. 8C, D). What’s more, Tumor Immune Dysfunction and Exclusion (TIDE) analysis [43] revealed that patients responding to immunotherapy had lower RNASEH2C expression, and those with low RNASEH2C levels were more sensitive to immunotherapy than those with high levels (Fig. 8E). Finally, we investigated the correlation between RNASEH2C expression in infiltrating macrophages within the HCC tissues of patients and Th1 cell activity. Our results demonstrated that as RNASEH2C expression levels in macrophages increased, the expression of TNF-α and IFN-γ in CD4^+^ T cells decreased (Fig. S7B). Therefore, we believed that RNASEH2C in macrophages was an immunosuppressive target, and inhibiting its expression would improve the immune efficacy of T cells.

## Discussion

RNASEH2C, an RNA-DNA hybrid-specific nuclease, is crucial for RNA metabolism and genome stability [44]. It plays roles in immune and inflammatory responses, and mutations in RNASEH2C disrupt nucleotide metabolism, leading to the accumulation of nucleic acids [44]. This triggers the cGAS-STING pathway, causing excessive type I IFN production and autoimmune responses [44]. Although RNASEH2C is linked to autoimmune diseases [44], its role in tumor immunity is less understood. Our study found that in HCC, RNASEH2C promoted tumor progression by inhibiting tumor-associated antigen presentation, weakening the adaptive immune response and worsening HCC progression. We discovered that RNASEH2C acted as both a nuclease with transcriptional regulatory functions and a lysosomal inhibitor affecting the post-translational modification of the cytoskeletal protein RAI14, broadening our understanding of RNA nucleases’ roles.

Macrophages, crucial to innate immunity, have a dual role in the tumor microenvironment, particularly in HCC [45]. They influence tumor progression and immune therapy by presenting antigens. In HCC, tumor-associated macrophages (TAMs) promote immune escape by inducing T cell tolerance via MHC II molecules [45]. However, their antigen-presenting ability is often impaired in this immunosuppressive environment, leading to inadequate T cell activation [45]. Recent studies suggested that targeting TAMs’ phenotypic reprogramming, using agents like CSF-1R inhibitors [46] or CD40 agonists [47], can boost their antigen-presenting efficiency and activate anti-tumor T-cell responses, offering a novel strategy for HCC immunotherapy. Our study showed that RNASEH2C hindered tumor-associated antigen uptake and MHC-II complex formation by inhibiting RAI14, which weakened Th1 cell activation and promoted HCC escape. Understanding macrophage antigen presentation in HCC’s immune regulation is crucial for developing combined immunotherapy strategies.

RAI14, an evolutionarily conserved transmembrane protein, has recently been identified as a significant regulator of macropinocytosis [33]. Macropinocytosis, a non-selective endocytic pathway, facilitates the efficient uptake of extracellular substances through the formation of large membrane invaginations and is intricately linked to immune responses [48]. Most research has focused on RAI14’s role in inflammation, leaving its function in antigen presentation largely unexplored. The regulation of RAI14 in macrophages is also not well understood. This study showed that RNASEH2C, a macrophage-specific transcription factor, aided in the lysosomal degradation of RAI14 at the post-translational level, with HSC70 and CMTM6 playing key roles. RAI14 enhanced T cell-mediated anti-tumor responses by processing and presenting tumor-associated antigens to Th1 cells. This finding could be important for boosting macrophage-mediated tumor immunity and warrants further study.

Lysosomal degradation is orchestrated by various genes [49–51]. TFEB and TFE3 facilitate lysosomal synthesis, maturation, and functional maintenance by activating the expression of lysosome-related genes [49]. Furthermore, the activation of the cGAS-STING pathway induces the nuclear translocation of TFEB, thereby promoting the expression of lysosome-related genes and enhancing lysosomal degradation activity [50]. Additionally, SMURF1 contributes to the autophagic degradation of damaged lysosomes and maintains cellular homeostasis through its interaction with the PPP3/calcineurin complex [51]. Our study found that, unlike previous research, HSC70 aided in degrading RAI14 via endosomal and lysosomal pathways. HSC70 and CMTM6 both bound to RAI14, with HSC70’s degradation of RAI14 relying on CMTM6. Notably, removing CMTM6 increased the interaction between RAI14 and HSC70, suggesting a collaborative role in RAI14’s lysosomal degradation. Thus, targeting HSC70 inhibition or boosting CMTM6 could enhance lysosomal circulation of RAI14.

This study, however, presented certain limitations. Firstly, our analysis was confined to examining the influence of RNASEH2C on tumor immunity in macrophages via the lysosomal degradation pathway and antigen presentation function, without extending the evaluation to other potential pro-tumor effects. Pathway enrichment analysis suggested that *Rnaseh2c*^+^ macrophages were also associated with ferroptosis and cholesterol metabolism, among other processes. Consequently, investigating the immunomodulatory effects of RNASEH2C with a focus on ferroptosis, particularly centered on lipid peroxidation, warrants further exploration. Secondly, we identified two proteins, PRDX1 and RAI14, that bound to HSC70 or CMTM6. Our research has thus far concentrated solely on the role of RAI14, leaving the involvement of PRDX1 in RNASEH2C-mediated tumor regulation unclear. Given the pivotal role of PRDX1 in maintaining lipid flux in macrophages [52], future studies could profitably investigate the ferroptosis-PRDX1 pathway to further elucidate the function of RNASEH2C. Finally, this study exclusively employed gene manipulation to assess the therapeutic potential of RNASEH2C, highlighting a notable gap in research concerning its pharmacological inhibitors or small molecule modulators. Future research endeavors will focus on utilizing drug virtual screening and compound libraries to identify effective inhibitors of RNASEH2C, thereby enhancing the assessment of its viability as a translational therapeutic target.

In conclusion, our study showed that the HSC70/CMTM6-RAI14 interaction was crucial for RNASEH2C and TRAF3IP1 to suppress macrophage antigen presentation (Fig. 8F). Additionally, *Rnaseh2c*^+^ macrophages, identified as non-classically polarized, mainly promote tumor growth by inhibiting antigen presentation.

## Supplementary information

Supplementary Information

Raw blot

## Data Availability

The raw protein mass spectrometry data or RNA sequencing data that support the findings of this study are deposited in PRJNA880758 (single-cell RNA sequencing, https://www.ncbi.nlm.nih.gov/bioproject/?term=PRJNA880758), PRJCA036752 (mass spectrum, https://ngdc.cncb.ac.cn/bioproject/browse/PRJCA036752), and CRA023407 (bulk RNA sequencing, https://ngdc.cncb.ac.cn/gsa/browse/CRA023407).
