# Supplementary material for: RNASEH2C enhances TRAF3IP1 to degrade RAI14 in lysosomes thus hindering macrophage antigen presentation and advancing liver cancer
Source: Cell Death Dis. 2025 Dec 8;17(1):92. doi: 10.1038/s41419-025-08305-5 (PMC12830818; doi:10.1038/s41419-025-08305-5)
Supplement: Supplementary file 1 — Supplementary Information [file 41419_2025_8305_MOESM1_ESM.docx]

***Supplementary Information for***

**RNASEH2C enhances TRAF3IP1 to degrade RAI14 in lysosomes thus hindering macrophage antigen presentation and advancing liver cancer**

**Table of contents**

**Supplementary Methods**2

**Supplementary Figures**22

**Supplementary Table**36

**Supplementary References**37

**Supplementary Methods**

**Patient samples**

This study was approved by the Ethics Committee of Fujian Medical University Union Hospital (approval number [2025]270). Patients/participants have provided written informed consent to participate in this study. Hepatocellular carcinoma (HCC) samples were derived from HCC patients undergoing hepatectomy in Fujian Medical University Union Hospital. The inclusion criteria required participants to satisfy the following conditions at the time of enrollment: (1) be at least 20 years of age, (2) have a diagnosis of primary HCC, and (3) have not undergone radiation, chemotherapy, or any other adjuvant therapy. Conversely, the exclusion criteria stipulated that participants were excluded if they (1) had a history of other cancers, (2) had participated in other clinical trials or used any investigational drugs within the preceding 30 days, and (3) were pregnant. The patient information was presented in Table S1.

**Bioinformatics and Computational Analysis**

The promoter region of human or mouse TRAF3IP1 was characterized as the 2000 bp upstream of its 5’ transcriptional start site. To ensure comparability, the tag counts at each genomic position were adjusted to normalize the overall tag count. The normalized data were visualized using the UCSC Genome Browser (http://genome.ucsc.edu/)(1).

**Plasmids**

Plasmids with a propensity for forming R-loops, such as pFC53, the mouse *c-Myc* promoter, and the mouse *Traf3ip1* promoter, were engineered by inserting the genomic sequences of the mouse 1.2 kb DNA segment from the murine *Airn* CpG island (CGI)(2), the *c-Myc* promoter, and the *Traf3ip1* promoter, respectively, into the pBluescript II SK (+) vector (212205, Stratagene, CA, USA). Transcripts of truncated protein sequences for CMTM6, HSC70, and RAI14 in mice, along with coding sequences for the mouse genes *Cmtm6*, *Hk2*, *Hsc70*, *Lamp2a*, *Rai14*, *Rnaseh2c*, and *Tsg101*, were cloned into the pLVX-M-puro vector (125839, Addgene, MA, USA). Furthermore, transcripts of both wild-type (WT) and 3KA mutant sequences of the mouse protein HSC70, as well as physiological and reverse transcripts of the mouse *Traf3ip1* promoter, were also cloned into the pLVX-M-puro vector.

**Mouse model**

All animal experiments followed protocols approved by Fujian Medical University’s Ethics Committee (approval number IACUC FJMU 2024-0082). Mice were kept in ventilated cages in a pathogen-free environment at room temperature (RT) and 50%-60% humidity. Six mice were randomly assigned per group.

1. Construction of subcutaneous tumor model: Hep-53.4 cells (5 × 10^6^, Procell, China) were suspended in 100 μL DPBS (14190144, Gibco, NY, USA) and injected subcutaneously into 10-week-old female C57BL/6J mice (~23g) (GemPharmatech, China). Tumor formation was observed when the control group’s average tumor diameter reached 2 mm. When a mouse’s tumor reached 2 cm in diameter, it was euthanized with CO_2_, and the tumor was removed, photographed, and weighed. Tumor volume was calculated using the formula: (long axis × wide axis²) × 0.5.
2. Construction of carcinoma in situ: 1 × 10^6^ Hep-53.4 cells per mouse, and an injection volume of 50 μL. 10-week-old female C57BL/6J mice, with an average weight of approximately 23 g, were anesthetized using 0.8% Pentobarbital Sodium (60 mg/kg, P3761, Merck, Germany) administered intraperitoneally. Following the induction of anesthesia, the mice were secured on an operating board, and the surgical site was thoroughly disinfected. A longitudinal incision was made approximately 1 cm inferior to the xiphoid process to access the abdominal cavity, and the left lobe of the liver was gently exposed using a sterile cotton swab. The liver lobe was stabilized with the swab, and a 1 mL micro-syringe was inserted at an angle of 15-30° along the liver surface, penetrating approximately 0.5 cm into the liver. The cell suspension was injected slowly, after which the needle was withdrawn gradually. A sterile cotton swab was then applied to the injection site to control bleeding. Subsequently, the liver was carefully repositioned within the abdominal cavity, and the abdominal incision was sutured in layers and sterilized. The mice were placed on an electric blanket to maintain body temperature until they regained consciousness, after which they were returned to their cages for monitoring of vital signs and body weight. Euthanasia was performed on mice exhibiting behavioral abnormalities, such as collapse or hyperactivity, and significant weight loss. As with the euthanasia method for subcutaneous tumor model, CO_2_ was used to sacrifice mice.

**3)** Construction of primary HCC model: Briefly, a single dose of DEN (25 mg/kg, HY-N7434, MedChemExpress, NJ, USA) was injected into 15-day-old female C57BL/6J mice intraperitoneally (*i.p.*) to initiate tumor formation. At 4 weeks of age, CCl_4_ (0.5 mL/kg, HY-Y0298, MedChemExpress) was injected *i. p*. Twice a week with CCl_4_ for an additional 12 weeks. When HCC was formed in the liver, and confirmation was obtained by bimanual palpation and dissection. Anesthesia and euthanasia during the experiment: If an animal becomes listless and loses its appetite, it should be euthanized with CO_2_.

**4)** Construction of patient-derived orthotopic xenograft (PDOX) model: The study involving patients was approved by the Ethics Committee of Fujian Medical University Union Hospital (approval number [2025]270) and performed in accordance with the Helsinki Declaration and government policies. Briefly, mechanically minced pieces of fresh patient HCC tissue were plated on bottles at 37°C in Human Liver Organoid Culture Medium (abs9529, Absin, China) for up to 2 weeks. Organoids (passage 1) with a diameter of 300 µm were made with sterile DPBS at a concentration of 2×10^7^/mL, 1 × 10^6^ cells per mouse, and an injection volume of 50 μL. 10-week-old female huHSC-C-NKG-ProF mice (~23 g) (Caygen, China) were anesthetized with 0.8% Pentobarbital Sodium (60 mg/kg) by *i. p*. After anesthesia, the mice were fixed on the operating board, and the surgical site was disinfected. A longitudinal incision was made about 1 cm below the xiphoid process of mice to open the abdominal cavity, and the left lobe of the liver was gently exposed with a sterile cotton swab. Fix the liver lobe with a cotton swab, insert a 1 mL micro-syringe along the liver surface at 15-30°, penetrate the liver of about 0.5 cm, inject the cell suspension slowly, withdraw the needle slowly, and use a sterile cotton swab to lightly press the injection site to stop bleeding. The liver was then carefully placed back into the abdominal cavity, the abdomen was sutured layer by layer, and the incision was sterilized. Place the mice on an electric blanket until the mice wake up, then return them to their cages and observe the changes in the mice’s vital signs and body weight. Mice (passage 1) were maintained and sacrificed using CO_2_ when behavioral abnormalities (collapse, hyperactivity) and weight loss occurred. Organoids (passage 2) were further prepared from minced xenograft livers in the same manner as patient tissues and implanted for several generations. The PDOX model was established at passage 3, when tumor phenotype tended to stabilize.

**5)** Construction of macrophage conditionally knocked out (cKO) mice: To create the *Rnaseh2c*-cKO mice (T038962, GemPharmatech), Exons 1-4 of the *Rnaseh2c*-201 transcript were targeted as the flox region. For the *Traf3ip1*-cKO mice, Exons 1-17 of the *Traf3ip1*-201 transcript were selected. To create the *Rai14*-cKO mice (T020760, GemPharmatech), Exon 3 of the *Rai14*-201 transcript was targeted as the flox region. Knocking out these regions would induce the frameshift mutation, leading to premature translation termination and protein mutations. Fertilized eggs were implanted into female C57BL/6J mice to produce positive F0 mice. To achieve macrophage-specific cKO mice, floxed *Rnaseh2c, Traf3ip1*, or *Rai14* mice were bred with *Lyz2-iCre* transgenic mice (T003822, GemPharmatech). For animal studies with *Rnaseh2c*^flox/flox^*Lyz2-iCre* (*Rnaseh2c*-cKO), *Traf3ip1*^flox/flox^*Lyz2-iCre* (*Traf3ip1*-cKO), or *Rai14*^flox/flox^*Lyz2-iCre* (*Rai14*-cKO) mice, littermates with *Rnaseh2c*^flox/flox^, *Traf3ip1*^flox/flox^, or *Rai14*^flox/flox^ as control were used. Deletion of the floxed *Rnaseh2c* allele and WT *Rnaseh2c* exons was detected by PCR using the following primers: *Rnaseh2c*-5’arm (F-CCAAGAGGGGACATGTTAGGGAG, R-CCTCCTTCACTCCTGTCATGTACC), *Rnaseh2c*-3’arm (F-AACACCACGGGGATCATGGCT, R-TGCAAAGTTCTGGCCCCTCTTG). Deletion of the floxed *Traf3ip1* allele and WT *Traf3ip1* exons was detected by PCR using the following primers: Upstream primers for WT allele identification (F-gcagcatcctagcctaaggg, R-gaatcctactcagacgcgca), downstream primers for WT allele identification (F-tgtaaggatttcaagcctgtccc, R-ccttacaaggacacgggcta). Primers for KO identification (F-gcagcatcctagcctaaggg, R-ccttacaaggacacgggcta). Deletion of the floxed *Rai14* allele and WT *Rai14* exon was detected by PCR using the following primers: *Rai14*-5’arm (F-GTCTGAACAGCTCAGAGAATTGAGGC, R-GTAGGACTATGGGGTCAGCTTACTCAT), *Rai14*-3’arm (F-TCTGAGGCGGAAAGAACCAG, R-GGGCCATCCATACTCTCTTGAACC). Primers that produce macrophage-specific cKO: 5’-arm (F-GAACACACCTGGAAGATGCTCC, R-CATCCTTGGCACCATAGATCAGG), WT (F-GCTAAAGGCAGAAGGGAGAGACTC, R-ACTGATGCTTTCTGTCAAATGACCTC).

**6)** Construction of *Rnaseh2c* or *Traf3ip1*-conditional knock in (cKI) mice. Using CRISPR/Cas9 technology, a CAG-tdTomato-polyA expression frame was inserted into the *Rosa26* gene locus by homologous recombination. A homologous recombination vector containing a 5’ homology arm, a tdTomato expression frame, and a 3’ homology arm was constructed by In-Fusion cloning. Cas9 mRNA, gRNA, and donor vector were microinjected into fertilized eggs of female C57BL/6J mice to obtain mice with loxp sites and mate with *Lyz2-iCre* transgenic mice. Primers for *Rnaseh2c* or *Traf3ip1*-cKI identification: WT primers-used to identify the presence of WT alleles and Cas9 activity (F-ggagtgttgcaatacctttctgggagttc, R-tgtccctccaattttacacctgttcaattc). Transgene primers-used to identify whether the Donor integration into the genome (F-ctgtagggcgcagtagtccag, R-gctagaactagtggatctcgagcc), the amplified fragments of *Rnaseh2c* and *Traf3ip1* were 5683 and 7060 bp, respectively. 5’ junction primers-used to identify whether the Donor inserted into the *Rosa26* locus (Out-F-ctgcccgagcggaaacgccactgac, SA-R-cctggactactgcgccctacaga). 3’ junction primers-used to identify whether the Donor inserted into the *Rosa26* locus (Insert-F-ggctcgagatccactagttctagc, Out-R-ccattctcagtggctcaacaacac). *iCre* primers-used to determine whether the floxed cassette was removed by the *iCre* (pCAG-F-gcaacgtgctggttattgtg, SK-R-gccgctctagaactagtggatcc), the amplified fragments of *Rnaseh2c* and *Traf3ip1* were 3154 and 4531 bp, respectively.

**7)** Drug treatment: The drug injection protocol involved administering anti-CD163 antibody (15 mg/kg, A2565, Selleck, TX, USA), anti-human IgG1 antibody (15 mg/kg, A2051, Selleck), anti-mouse IgG1 antibody (15 mg/kg, A2106, Selleck), MOMIPP (10 μM, HY-119624, MedChemExpress), Nivolumab-anti-human-PD-1 (10 mg/kg, A2002, Selleck), Nurulimab-anti-human-CTLA-4/CD152 (10 mg/kg, A2859, Selleck), or Rapamycin (2 mg/kg, HY-10219, MedChemExpress) into the tail vein 20 days before the mice were sacrificed, and then every three days for a total of five doses.

**8)** Ad5f35 transfection macrophages: Macrophages were manufactured from autologous monocytes that were isolated from peripheral blood mononuclear cell concentrates of healthy human by counter-flow elutriation with Ficoll (E4035, Selleck). The monocytes were isolated with EasySep™ Human Monocyte Isolation Kit (100-0697, Stemcell, Canada). The monocytes were cultured with M-CSF (50 ng/mL, HY-P70488, MedChemExpress) and IL-4 (20 ng/mL, HY-P70445, MedChemExpress) to produce macrophages. The macrophages were transduced with a chimeric adenoviral vector, Ad5f35(3) (VB010000-9301bcw , VectorBuilder, China). This chimeric adenovirus was effective at transducing human macrophages(3). GFP was added as an indicator marker.

**Cell culture**

Hep-53.4 and RAW264.7 (C7505, Beyotime, China) cells were cultured in DMEM medium (11965092, Gibco). The medium was supplemented with 10% FBS (A5670701, Gibco) and 1% Penicillin/Streptomycin (15140148, Gibco). In vitro mouse HCC-infiltrating macrophage culture: Mouse macrophages were purified using flow cytometry sorting. Subsequently, macrophages were cultured in RPMI 1640 medium (11875093, Gibco), which was supplemented with 10% FBS, 1% Penicillin/Streptomycin, and 55 μM β-mercaptoethanol (21985023, Gibco). All cells used in this study were validated by short tandem repeat analysis (Beyotime). Mycoplasma PCR Detection Kit (C0301S, Beyotime) was used to detect whether there was mycoplasma contamination in cells and ensure no contamination. Cells were treated with AC220 (4 nM, HY-13001, MedChemExpress), CQ (20 μM, HY-17589, MedChemExpress), DMSO (HY-Y0320C, MedChemExpress), E64D (10 μM, HY-100229, MedChemExpress), Leupeptin (100 nM, HY-18234, MedChemExpress), MG-132 (10 mΜ, HY-13259, MedChemExpress) , NH_4_Cl (0.3 M, HY-Y1269, MedChemExpress), Spautin-1 (10 μM, HY-12990, MedChemExpress), U18666A (5 μg/mL, HY-107433, MedChemExpress), and 3-MA (5 mM, HY-19312, MedChemExpress) for 48 h, respectively.

**Calculation of immunotherapy response**

Tumor Immune Dysfunction and Exclusion (TIDE)(4) and SubMAP(5) were employed to explore whether human RNASEH2C could predicted the efficiency of a patient’s response to immunotherapy. TIDE(4) was used to measure the TIDE score of each HCC specimen in TCGA database, and then SubMAP algorithm(5) was performed to compare the difference in immunotherapy efficiency between high and low human RNASEH2C expression groups.

**Cell transfection**

For electrotransfection: Hep-53.4 or RAW264.7 cells were suspended in DMEM at a concentration of 10^7^ cells/mL. Plasmid DNAs were added into the suspension to achieve a final concentration of 10 mg/mL. The pulses were generated by using a NxT Electroporation System (NEON18SK, Thermo Scientific, CA, USA). After electrotransfection, samples were incubated at 37℃ for 10 min to promote endocytosis. For gene KO, CRISPR target sequence (designed by Ubigene, China): human-RNASEH2C (TTCCGGGACTGACGACCAAG), mouse-*Cmtm6* (GTGCACGACAGAGTGGATAC), mouse-*Hsc70* (GCATCATTCACCACCATGAA), mouse-*Lamp2a* (TTGCGCCTTTAACCGGAGAG), mouse-*Rai14* (GCGGAGAACATTGACAACTC), mouse-*Rnaseh2c* (GCGTGAAGAAGCGATCTACC), and mouse-*Vps4a* (TGGAGAAGCCGAACATACGG).

**DNA/RNA Immunoprecipitation (DRIP)**

In vitro transcription products were incubated with an anti-S9.6 antibody (ab234957, Abcam, UK) in a binding buffer composed of 10 mM NaPO_4_ (pH 7.0, 342483, Merck), 140 mM NaCl (S5886, Merck), and 0.05% Triton X-100 (T9284, Merck) at 4ºC overnight. Subsequently, the binding mixture was incubated with Protein A/G Magnetic Beads (88802, Thermo Scientific) for 1 h. The DNA bound to the complex was eluted using a buffer containing 50 mM Tris (pH 8.0, RDD008, Merck), 10 mM EDTA (17892, Thermo Scientific), 0.5% SDS (L3771, Merck), and 300 µg of Proteinase K (1.24568, Merck) at 50ºC for 30 min. The samples underwent Phenol/Chloroform/Isopentanol (77617, Merck) extraction followed by Ethanol (493511, Merck) precipitation. The immunoprecipitated DNA was then subjected to qPCR analysis. The upstream and downstream primers used to detect DNA fragments: mouse-*Airn* for R-loop formation (pFC53) (F-GGAGAAGGCGAAAGCAGAGA, R-TTGCCAAACACCTTCCACTC), mouse *c-Myc* promoter (F-GCGATCAGCTCTCCTGAAAA, R-ACACAGGGAAAGACCACCAG), and mouse *Traf3ip1* promoter (F-AGGCTCCAGGTCCTCAGTAG, R-CGGGCACAAAACTGTGAAGG).

**Flow cytometry analysis**

The cells were resuspended in Flow Cytometry Staining Buffer (00-4222-57, eBioscience, CA, USA), stained with fluorescent dye-conjugated antibodies for 30 min, and washed twice. Samples were collected using BD FACSAria™ III Cell Sorter (BD Biosciences, NJ, USA) and analyzed with FlowJo (BD Biosciences). Primary antibodies used in this study: anti-human-CD3e-BV786 (563918, BD Biosciences), anti-human-CD4-FITC (555346, BD Biosciences), anti-human-CD11b-BV786 (742642, BD Biosciences), anti-human-CD45-BV510 (563204, BD Biosciences), anti-human-CD68-BV421 (564943, BD Biosciences), anti-human-IFN-γ-APC (554702, BD Biosciences), anti-human-RNASEH2C (PA5-26311, Invitrogen, CA, USA), anti-human-TNF-α-BV421 (566275, BD Biosciences), anti-mouse-CD3e-BV786 (417-0031-82, Invitrogen), anti-mouse-CD4-FITC (11-0041-82, Invitrogen), anti-mouse-CD8a-eFluor450 (48-0081-82, Invitrogen), anti-mouse-CD11b-BV785 (101243, Biolegend, CA, USA), anti-mouse-CD45-BV510 (567800, BD Biosciences), anti-mouse-Ea52-68 (13-5741-82, Invitrogen), anti-mouse-F4/80-eFluor450 (48-4801-82, Invitrogen), anti-mouse-IFN-γ-PE (12-7311-82, Invitrogen), anti-mouse-IL-2-PerCP-Cy5.5 (45-7021-82, Invitrogen), anti-mouse-TNF-α-APC (17-7321-82, Invitrogen), anti-OVA (aa 323-339) (DPAB27549, Creative Diagnostics, NY, USA) antibodies. Isotype used in this study: Rat IgG1 kappa Isotype Control-APC (17-4301-82, Invitrogen), Rat IgG1 kappa Isotype Control-PE (12-4301-82, Invitrogen), and Rat IgG2b kappa Isotype Control-PerCP-Cyanine5.5 (45-4031-80, Invitrogen). Secondary antibodies used in this study: Goat anti-Rabbit IgG (H+L) Cross-Adsorbed Secondary Antibody-APC (A-10931, Invitrogen). Viability Dye used in this study: FVS780 (65-0865-14, Invitrogen). Fluorescent substrate used in this study: DQ-OVA (D12053, Invitrogen).

**Flow cytometry sorting**

The purification of OT-II T cells was conducted through flow cytometry sorting. Spleens from OT-II mice (004194, The Jackson Laboratory, ME, USA) were enzymatically digested for 45 min at 37℃ using a solution containing 1 mg/mL Collagenase Type IV (17104019, Gibco) and 200 U/mL DNase I (HY-108882, MedChemExpress). Naive OT-II T cells were then sorted based on specific markers, characterized as CD45^+^ CD3e^+^ CD4^+^ CD62L^high^ CD44^low^ CD25^-^. Mouse HCC-infiltrating macrophages were then sorted based on specific markers, characterized as CD45^+^ CD11b^+^ F4/80^high^. The cells were resuspended in Flow Cytometry Staining Buffer, stained with fluorescent dye-conjugated antibodies for 30 min, and washed twice. Samples were collected using the BD FACSAria™ III Cell Sorter and analyzed with FlowJo. During cell sorting, the concentration was maintained at 1 × 10^7^ cells/mL, with a pressure of 60 psi, a 70 μm nozzle, and a maximum rate of 20,000 events/s. Antibodies used in this study: anti-mouse-CD3e-PE (12-0031-82, Invitrogen), anti-mouse-CD4-FITC (11-0041-82, Invitrogen), anti-mouse-CD11b-BV785, anti-mouse-CD25-PE-Cy7 (25-0251-82, Invitrogen), anti-mouse-CD44-BV650 (416-0441-82, Invitrogen), anti-mouse-CD45-BV510, anti-mouse-CD45-APC (17-0451-82, Invitrogen), anti-mouse-CD62L-BV421 (404-0621-82, Invitrogen), and anti-mouse-F4/80-eFluor450 antibodies.

**Immunoblotting and immunoprecipitation**

The cells were washed with DPBS, lysed using Novex Tricine SDS Sample Buffer (LC1676, Invitrogen) with 1% PMSF (36978, Thermo Scientific), boiled for 20 min, and analyzed by sodium dodecyl sulfate-polyacrylamide gel electrophoresis. For immunoprecipitation, lysate was incubated with primary antibodies for 1 h at 4°C, then with Protein A/G Magnetic Beads for 1 h at 4°C. The complexes were washed and subjected to electrophoresis. Antibodies used in this study: anti-AKT (44-609G, Invitrogen), anti-ARG1 (PA5-29645, Invitrogen), anti-CD80 (PA5-85913, Invitrogen), anti-CD86 (MA5-15697, Invitrogen), anti-CD163 (ab182422, Abcam), anti-CMTM6 (ab264067, Abcam), anti-ERK1/2 (13-6200, Invitrogen), anti-Flag (MA1-91878, Invitrogen), anti-GFP (MA5-15256, Invitrogen), anti-HK2 (PA5-29326, Invitrogen), anti-HSC70 (MA1-26078, Invitrogen), anti-JNK (AHO1362, Invitrogen), anti-LAMP2A (51-2200, Invitrogen), anti-mTOR (AHO1232, Invitrogen), anti-p38 (33-1300, Invitrogen), anti-p65 (51-0500, Invitrogen), anti-p-AKT (MA5-38243, Invitrogen), anti-p-ERK1/2 (14-9109-82, Invitrogen), anti-p-JNK (700031, Invitrogen), anti-p-mTOR (MA5-57588, Invitrogen), anti-p-p38 (MA5-56575), anti-p-p65 (MA5-15160, Invitrogen), anti-p-STAT1 (33-3400, Invitrogen), anti-p-STAT3 (MA5-15193, Invitrogen), anti-p-S6 (14-9007-82, Invitrogen, Invitrogen), anti-p-S6K (MA5-15202, Invitrogen), anti-RAI14 (PA5-57887, Invitrogen), anti-RNASEH2C (PA5-100321, Invitrogen), anti-STAT1 (AHO0832, Invitrogen), anti-STAT3 (MA1-13042, Invitrogen), anti-S6 (MA5-15123, Invitrogen), anti-S6K (MA5-15141, Invitrogen), anti-TRAF3IP1 (PA5-30507, Invitrogen), anti-TSG101 (MA1-23296, Invitrogen), anti-VPS4A (PA5-110573, Invitrogen), anti-β-tubulin (MA5-16308, Invitrogen), and anti-6×His (MA1-21315, Invitrogen) antibodies.

**Immunofluorescence**

The cells were cultured on slides and fixed using Image-iT fixative (I28800, Thermo Scientific) for 20 min. Subsequently, they were incubated with DPBS containing 0.1% Triton X-100 (HFH10, Invitrogen) at RT for 1 h. Following this, the cells underwent three washes with DPBS and were blocked with 5% BSA (37520, Thermo Scientific) at RT for 1 h. The cells were then incubated overnight at 4°C with primary antibodies, including anti-Calnexin (MA5-31501, Invitrogen), anti-EEA1 (MA5-14794, Invitrogen), anti-GM130 (MA5-35107, Invitrogen), anti-LAMP1 (MA1-164, Invitrogen), anti-MHC II molecules (MA5-46188, Invitrogen), anti-RAB7A (PA5-52369, Invitrogen), anti-RAB11 (71-5300, Invitrogen), and anti-RAI14 (PA5-57888, Invitrogen) antibodies. After three washes with DPBS, the cells were incubated for 1 h with secondary antibodies: Goat anti-Rabbit IgG (H+L) Highly Cross-Adsorbed Secondary Antibody-Alexa Fluor™ 546 (A-11035, Invitrogen), and Goat anti-Rabbit IgG (H+L) Cross-Adsorbed Secondary Antibody-Alexa Fluor™ 488 (A-11008, Invitrogen). Nuclei were labeled with DAPI (HY-D0814, MedChemExpress). Images were captured using FV3000 OLYMPUS microscope (Olympus, Japan).

**In vitro transcription for R-loop formation**

Plasmids pFC53, mouse *c-Myc* promoter, or mouse *Traf3ip1* promoter were subjected to in vitro transcription using T3 RNA polymerase (RPOLT3-RO, Merck) at 37ºC for 1 h, after which the reaction was terminated by incubating at 65ºC for 10 min. The resulting samples were divided equally and treated either with RNase A (RNASEA-RO, Merck) in conjunction with RNase H (10786357001, Merck) or with RNase A alone at 37ºC for 30 min, followed by treatment with Proteinase K at 37ºC for 30 min. Subsequently, the samples were purified using Phenol/Chloroform/Isopentanol extraction, and the precipitated DNA was reconstituted in water.

**In vitro transcription for R-loop inhibition**

To inhibit the formation of R-loop structures within macrophages, *E. coli* *Topo1* was applied. Acquire the coding sequence of the *E. coli* *topA* gene, which encodes TOPO1. Subsequently, clone this sequence into the Tetracycline-regulated expression system, pcDNA™4/TO Vector (V102020, Invitrogen). The employment of Tetracycline-inducible expression system was essential, as the continuous high-level expression of *E. coli* *Topo1* could interfere with the normal physiological functions of the cells(6). The pcDNA™4/TO vector inherently lacks a robust promoter for driving the expression of the target gene. Fundamentally, the pcDNA™4/TO vector incorporates the Tetracycline operator 2 (TetO2) sequence. In the absence of Tetracycline, the expression of the Tetracycline repressor (TetR) protein was facilitated by the pcDNA™6/TR Vector (V102520, Invitrogen). The TetR protein forms a dimer with a high affinity for the TetO2 sequence, thereby obstructing the assembly of the transcriptional machinery and consequently inhibiting gene expression. Upon the introduction of Tetracycline, this compound binds with high affinity to the TetR protein, inducing a conformational change that results in its dissociation from the TetO2 sequence. The removal of this inhibitory factor permits the downstream CMV promoter to initiate the transcription of the target gene. For experimental purposes, macrophages were co-transfected with the pcDNA™4/TO and pcDNA™6/TR plasmids. 48 h post-transfection, screening was initiated using a medium containing two antibiotics: Blasticidin S HCl (2 μg/mL, A1113903, Gibco) was employed to select for cells expressing TetR, and Zeocin (50 μg/mL, R25001, Gibco) was used to select for cells containing the pcDNA™4/TO plasmid. The antibiotic selection process continued for 2 wk until distinct resistant clones were established. Collect the clones and amplify them. Inoculate the stable cell lines into 6-well plates. Once the cell density reached 70% confluence, introduce Doxycycline (HY-N0565, MedChemExpress) into the culture medium at a concentration of 1 μg/mL. Elevated levels of protein expression can be observed within 24 h.

**Isolation of tumor tissue**

The primary HCC tissues were cut into small pieces with a diameter of 1-3 mm and digested in tissue digestion solution (0.4 mg/mL Collagenase Type IV, 10 mg/mL DNase I, 10% FBS, DPBS) at 37°C for 30 min. The reaction was terminated by adding EDTA to a final concentration of 5 mM. To make the sample uniform, pipette repeatedly with a 23 G needle until a homogenate was formed. The homogenate was passed through a 70 mm mesh and centrifuged at 400 × g for 8 min at 4°C. Centrifuge at 900 × g for 30 min in 30% Ficoll to collect the middle white cell layer. The cells were washed twice with DPBS for subsequent analysis.

**Library construction of single-cell RNA Sequencing (scRNA-Seq)**

scRNA-Seq libraries were created with Chromium Single Cell 3’ Reagent Kit (PN-1000121, 10× Genomics, CA, USA). Initially, the cells were encapsulated in droplets with gel beads containing unique primers with 10× Genomics barcodes, unique molecular identifiers (UMI), and poly(dT) sequences. Reverse transcription created barcoded full-length cDNA, which was then cleaned after emulsion disruption. Bulk cDNA was amplified and purified. Sequencing libraries were prepared using Chromium Next GEM Single Cell 3’ Reagent Kits (CG000206, 10× Genomics), and 10K Genomics (Shanghai, China) analyzed the data.

**Basic processing of scRNA-Seq data**

**1)** Gene quantification quality control and preprocessing involved using Cell Ranger for initial checks and Seurat(7) for further quality control. High-quality cells were filtered based on specific criteria: gene values >200, UMI values >1000, log_10_GenesPerUMI >0.7, mitochondrial UMI ratio <5%, and red blood cell gene ratio <5%. DoubletFinder(8) was used to eliminate doublets. Data normalization was performed with NormalizeData(7). **2)** Dimensionality reduction and clustering involved using FindVariableGenes(7) to select the top 2000 variable genes, applying Principal Component Analysis, and visualizing results with Uniform Manifold Approximation and Projection (UMAP)(9). **3)** Marker genes were identified with FindAllMarkers and visualized using FeaturePlot(7). **4)** SingleR(10) matched the unknown cell’s expression profile with a reference dataset to identify cell type based on the highest correlation. **5)** Differentially expressed genes were identified using FindMarkers(7), selecting those with *P* value < 0.05 and FoldChange > 2.

**Advanced analysis of scRNA-Seq data**

Single-Cell Regulatory Network Inference and Clustering (SCENIC) analysis: To characterize each macrophage subpopulation’s transcriptional state, we used SCENIC(11), a method for building gene regulatory networks and identifying cell states. SCENIC(11) involved three steps: GRNboost2(12) identified genes co-expressed with transcription factors, RcisTarget performed motif analysis to form regulatory modules, and AUCell quantified each module’s activity in each macrophage subpopulation, producing an AUC score to evaluate their characteristics.

**Liquid Chromatography-Tandem Mass Spectrometry (LC-MS/MS)**

Cells were washed with DPBS and subsequently lysed using 1 mL IP lysis buffer (87787, Thermo Scientific) for 30 min. The lysates were treated with an anti-HSC70 antibody or an anti-CMTM6 antibody. The samples were incubated on a rotating wheel at 4°C for 6 h to facilitate binding. The protein-antibody complexes were subsequently isolated using Protein A/G Magnetic Beads. The complexes were washed three times with IP lysis buffer, with each washing lasting 5 min. Following washing, the protein-antibody-bead complexes were digested with Trypsin (27250018, Gibco), and the proteins were identified and quantified using LC-MS/MS.

**Molecular docking analysis of protein with protein**

HDOCK(13) was used as a molecular docking program to analyze the interaction among proteins. PyMol was used to separate the original ligand and protein structure, dehydrate to remove organic matter, and then the Prepare module of Discovery Studio was used to prepare the protein, such as hydrogenation and protonation. LigPlus(14) was performed to analyze the forces between two proteins in two dimensions. The protein interaction interface was analyzed using the Analysis Interface module of Discovery Studio, and PyMol was applied to draw the interacting amino acid residues between two proteins.

**qPCR**

Total RNA was extracted and purified from the cells using TRIzol Plus RNA Purification Kit (2183555, Invitrogen). cDNA was synthesized from RNA using RevertAid Reverse Transcriptase kit (K1691, Thermo Scientific). qPCR was conducted using SYBR Green Master Mix (A46012, Applied Biosystems, CA, USA). Data analysis was performed using the comparative Ct method, with β-tubulin cDNA serving as internal control. The sequences of the primers utilized were as follows: mouse-*Arg1* (F-CTCCAAGCCAAAGTCCTTAGAG, R-AGGAGCTGTCATTAGGGACATC), mouse-*Cd80* (F-TCAGTTGATGCAGGATACACCA, R-AAAGACGAATCAGCAGCACAA), mouse-*Cd86* (F-TCAATGGGACTGCATATCTGCC, R-GCCAAAATACTACCAGCTCACT), mouse-*Cd163* (F-ATGGGTGGACACAGAATGGTT, R-CAGGAGCGTTAGTGACAGCAG), mouse-*Cmtm6* (F-TGTCTCCACCCATTCTGGGA, R-AGCATGACAACGAAGTCCAGT), mouse-*Hsc70* (F-TCTCGGCACCACCTACTCC, R-CCCGATCAGACGTTTGGCA), mouse-*Rai14* (F-AAGCAAAGTTTCGGAAGAGCG, R-CATGACCTTGAGGCATTCCAC), mouse-*Rnaseh2c* (F-GCAGACGGGAAACAGCGTATT, R-CGTGAAGAAGCGATCTACCG), mouse-*Traf3ip1* (F-GAGGGTGAAGGACCGAGAC, R-GTCGAGGACCTAGATGCTCC), and mouse-*β-tubulin* (F-GTGGTTTTGATTCTCCTGTGTGC, R-GCCTTGTACCCATCAGGGA).

**Transcriptome sequencing**

Total RNA was extracted and purified with TRIzol Plus RNA Purification Kit. RNA-seq libraries were constructed with VAHTS® mRNA-seq V3 Library Prep Kit for Illumina (NR611, Vazyme). Sequencing was performed using Illumina NovaSeq 6000 platform (provided by 10K Genomics, China), and the sequencing depth of each sample was 6G bases. Kyoto Encyclopedia of Genes and Genomes (KEGG)(15) enrichment analysis was performed to analyze the changes in biological functions after *Rnaseh2c*-cKO or the characteristics of *Rnaseh2c*^+^ macrophages. KEGG enrichment analysis process comprises four distinct steps: Step 1: Data Processing: This involves quality control, comparison, and quantification of the initial sequencing data to ascertain the count number of each gene. Step 2: Differential Analysis: Employing DESeq2(16), a comparative analysis was conducted to determine the expression differences (Log_2_FoldChange) of each gene, alongside calculating the false discovery rate (FDR) for the statistical test. Thresholds were typically established, with significant differentially expressed genes identified based on criteria such as Log_2_FoldChange > 1 and FDR < 0.05. The gene IDs of these differentially expressed genes constitute the primary input for the subsequent enrichment analysis. Step 3: Gene ID Conversion: This step involves the uniform conversion of Gene IDs to KEGG Gene IDs. Step 4: Enrichment Analysis: The enrichment analysis was performed, followed by the application of the Benjamini-Hochberg method for multiple hypothesis correction.

**Tumor-associated antigen peptide loading assay**

Mouse HCC-infiltrating macrophages were isolated from murine tumor tissues using flow cytometry sorting. Cells were exposed to 20 mM sodium azide (S2002, Merck) and 50 mM 2-deoxy-D-glucose (HY-13966, MedChemExpress) for 30 min to inhibit the recycling of MHC II molecules and facilitate the binding of these molecules to peptides. Subsequently, the cells were pulsed with Ea52-68 (AS-61621, Anaspec, CA, USA), an autopeptide derived from the H2E molecule, at a concentration of 25 μM for 1 h. Following this incubation period, the cells were washed with DPBS and incubated with an anti-EA52-68 antibody that recognizes the EA52-68/I-Ab complex. Then, the cells were subjected to flow cytometry analysis.

**Tumor-associated antigen uptake and degradation assay**

Mouse HCC-infiltrating macrophages were exposed to 3 μg/mL of OVA_323-339_ (HY-P0286, MedChemExpress) for 2 h, followed by two washes with DPBS. Subsequently, they were co-cultured with OT-II T cells, which had been sorted via flow cytometry, for 3 d. The expression of OVA_323-339_ in macrophages was subsequently assessed through immunofluorescence, while cytokine levels in OT-II T cells were measured using flow cytometry. For the determination of tumor-specific antigen degradation, the isolated macrophages were incubated with 50 μg/mL of DQ-OVA for 2 h. DQ-OVA was a self-quenching OVA conjugate that emits green fluorescence upon proteolytic cleavage. Next, cells were washed with DPBS, and the release of DQ-OVA_323-339_ was analyzed using flow cytometry.

**Supplementary Figures**


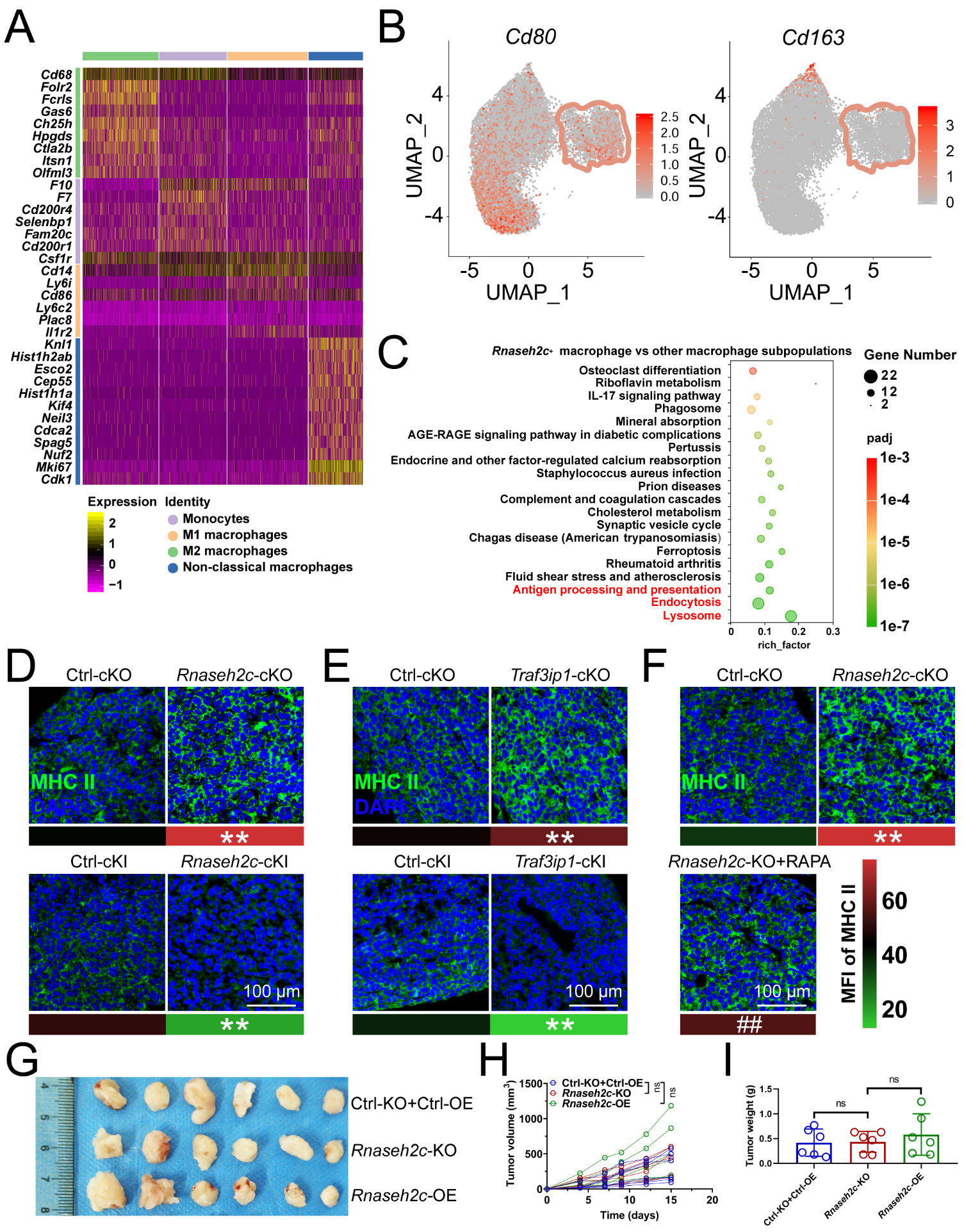


**Fig. S1** RNASEH2C promoted tumor growth by inhibiting the mTOR pathway. **A** Gene expression patterns (Y-axis) across macrophage clusters (X-axis) were depicted, with color representing average expression levels (*n* = 4). Mouse primary HCC was constructed and tumor tissues were collected for single-cell RNA sequencing. **B** UMAP plot, color coding for the expression of the marker genes (gray to red) for the indicated macrophage subtype (*n* = 4). **C** KEGG pathway analysis of differentially expressed genes in *Rnaseh2c*^+^ macrophages in comparison to monocytes, as well as M1 and M2 macrophages (*n* = 4). **D, E** Impact of *Rnaseh2c* (D) or *Traf3ip1* (E) -cKO or -cKI on the internalization of MHC II molecules by mouse HCC-infiltrating macrophages (*n* = 3). DEN and CCl_4_ were infused into mice to construct primary cancers, with observations made on the mice’s condition and survival time recorded. Upon the death of the mice, HCC tissues were collected for further analysis. **F** Role of *Rnaseh2c*-cKO and Rapamycin in the internalization of MHC II molecules by mouse HCC-infiltrating macrophages (*n* = 3). DEN and CCl_4_ were administered to mice to induce primary cancer formation. Rapamycin (2 mg/kg) was subsequently injected through the tail vein. Observations regarding the condition of the mice and their survival times were systematically recorded. Upon the demise of the mice, HCC tissues were collected for further analysis. **, *Rnaseh2c*-cKO vs Ctrl-cKO; ##, *Rnaseh2c*-cKO+Rapamycin vs *Rnaseh2c*-cKO. **G-I** Effects of knockout and overexpression of *Rnaseh2c* in Hep-53.4 cells on subcutaneous tumor growth (*n* = 6). Wild-type, *Rnaseh2c* knockout, and *Rnaseh2c* overexpression Hep-53.4 cells were implanted into the left axillary region of mice. Tumor volumes were monitored regularly, and mice were euthanized once the diameter of any tumor exceeded 2 cm, at which point the tumors were excised and weighed. (G) Representative. (H) Tumor growth curve. (I) Weight of the tumor. **D-F, H, I** represented mean ± SD analyzed by unpaired *t* test. ***P* < 0.01, ^##^ *P* < 0.01. cKI, conditional knock in; cKO, conditional knockout; HCC, hepatocellular carcinoma; KEGG, Kyoto Encyclopedia of Genes and Genomes; UMAP, Uniform Manifold Approximation and Projection.


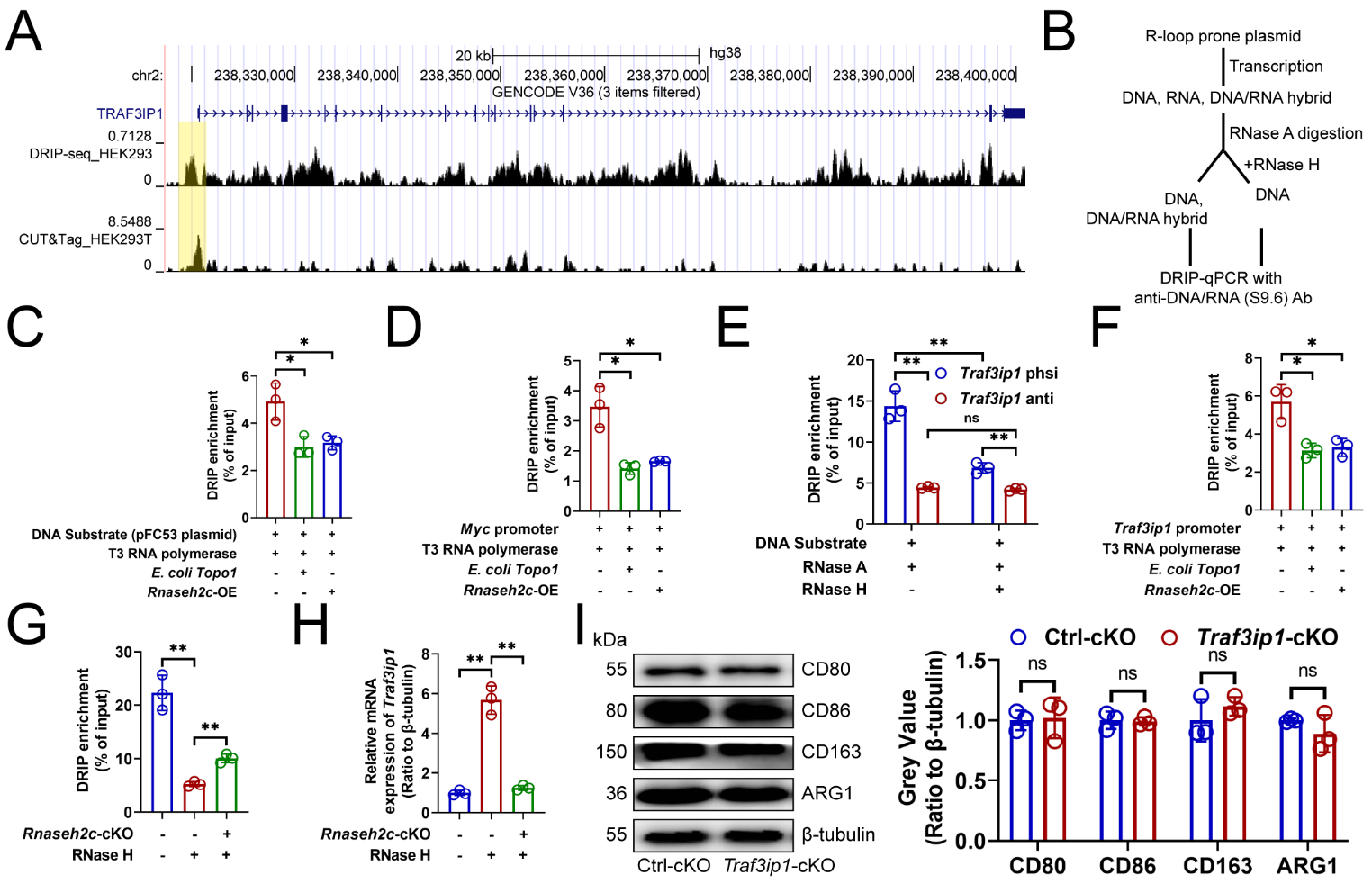


**Fig. S2** RNASEH2C enhanced the transcriptional activity of TRAF3IP1 by disrupting the R-loops on its promoter region. **A** The DRIP-seq data from HEK293 cells and the CUT&Tag data from an anti-S9.6 antibody pull-down of HEK293 cells available in the UCSC database were utilized to illustrate the presence of R-loops on the promoter and throughout the genome of the human TRAF3IP1 gene. **B** The workflow for in vitro R-loop detection using DRIP-qPCR involved cloning R-loop-prone regions into pBluescript II SK (+) vectors. These plasmids were transcribed in vitro using T3 RNA polymerase under standard conditions. The transcription products, comprising the DNA template, free RNA product, and DNA/RNA hybrid, were subjected to RNase A digestion to remove RNA. The remaining samples were then equally divided and either treated with or without RNase H. The final products, along with un-transcribed controls, were subjected to DRIP-qPCR using an anti-S9.6 antibody for quantification. **C** The role of RNASEH2C in mitigating co-transcriptional R-loops formed on a pFC53 DNA template (*n* = 3). The pFC53 plasmid underwent in vitro transcription in the presence of RNASEH2C, T3 RNA Polymerase, and *E. coli* *Topo1*, as specified. To quantify the levels of co-transcriptional R-loops, DRIP-qPCR was employed. **D** Role of RNASEH2C in R-loop formation on the mouse *c-Myc* promoter during in vitro transcription (*n* = 3). The R-loop forming region of the *c-Myc* promoter was transcribed in its physiological orientation, with or without *E. coli* *Topo1* and RNASEH2C. The resulting DRIP DNA samples were subjected to qPCR analysis. **E** R-loops were generated in vitro on the mouse *Traf3ip1* promoter (*n* = 3). The R-loop forming region of the *Traf3ip1* promoter was cloned and transcribed in both the physiological and anti-physiological orientations. Each transcribed sample was divided, with one half treated with RNase H. **F** The in vitro transcribed samples were immunoprecipitated using an anti-S9.6 antibody, and the DRIP DNA was analyzed via qPCR (*n* = 3). The R-loop forming region of the mouse *Traf3ip1* promoter was transcribed in the physiological direction, regardless of the presence or absence of *E. coli* *Topo1* and RNASEH2C. **G, H** In *Rnaseh2c*-cKO cells, treatment with RNase H was performed (*n* = 3). (G) Genomic DNA from these cells was subjected to DRIP analysis utilizing an anti-S9.6 antibody. (H) The expression level of *Traf3ip1* mRNA in the aforementioned cells was quantified using qPCR. **I** Immunoblotting analyzed the effect of *Traf3ip1*-cKO on the expression of macrophage polarization markers in mouse HCC-infiltrating macrophages (*n* = 3). **C, G, H** represented mean ± SD analyzed by unpaired *t* test, **D-F, I** represented mean ± SD analyzed by Wilcoxon test. **P* < 0.05, ***P* < 0.01. cKO, conditional knockout; CUT&Tag, Cleavage Under Targets and Tagmentation; DRIP, DNA-RNA immunoprecipitation.


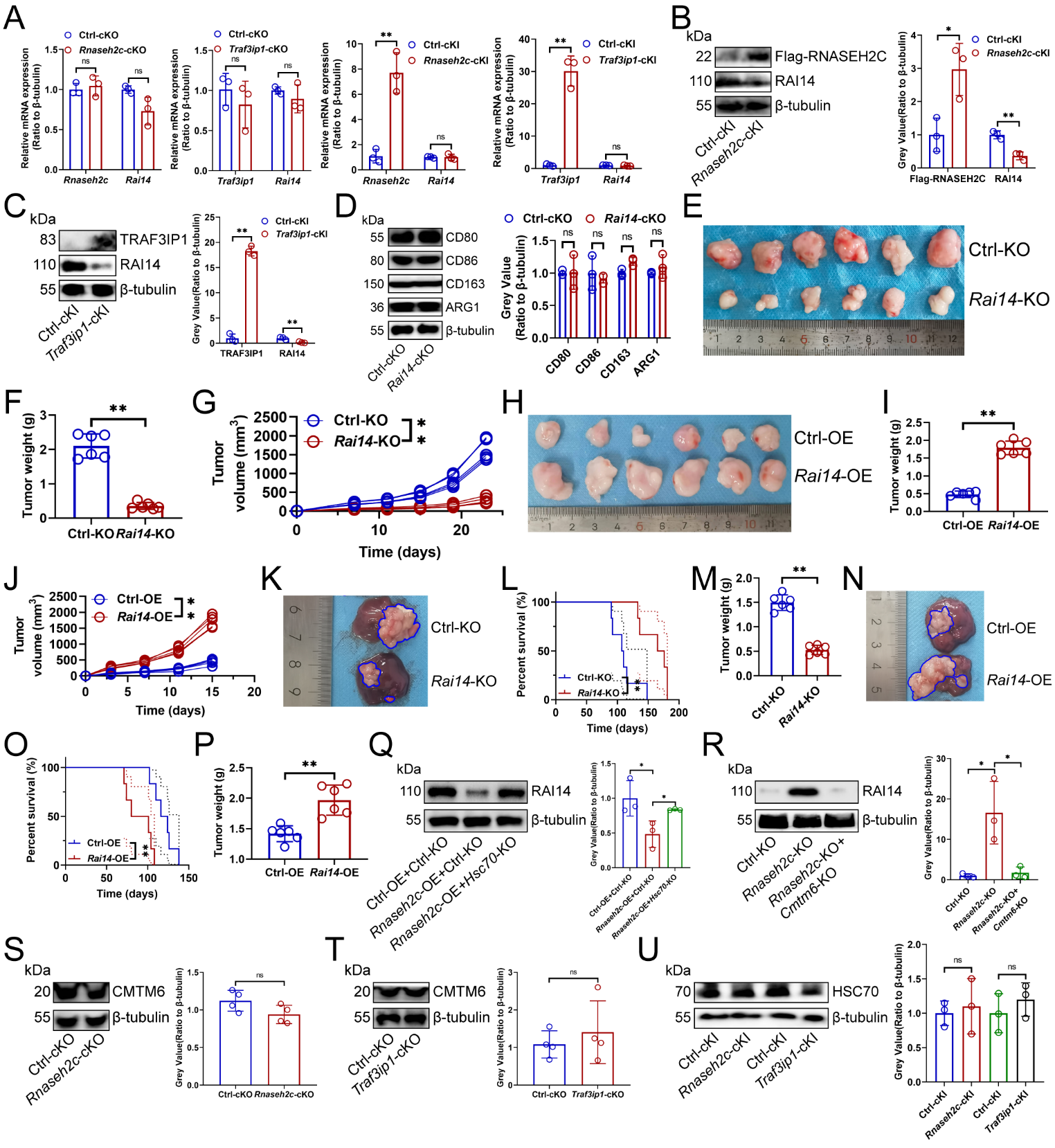


**Fig. S3** RNASEH2C facilitated RAI14 lysosomal degradation with HSC70 and CMTM6 being crucial. **A** qPCR illustrating the impact of *Rnaseh2c* or *Traf3ip1*-cKO or -cKI on the transcript levels of *Rai14* in mouse HCC-infiltrating macrophages (*n* = 3). **B, C** Immunoblotting analyzing the effect of *Rnaseh2c* (B) or *Traf3ip1* (C) -cKI on the expression of RAI14 protein in mouse HCC-infiltrating macrophages (*n* = 3). **D** The impact of *Rai14*-cKO on mouse HCC-infiltrating macrophage polarization (*n* = 3). **E-G** The consequences of *Rai14*-KO in HCC cells on the growth of subcutaneous tumors (*n* = 6). Wild-type and *Rai14* knockout Hep-53.4 cells were implanted into the left axillary region of mice. Tumor volumes were monitored regularly, and mice were euthanized once the diameter of any tumor exceeded 2 cm, at which point the tumors were excised and weighed. (E) Representative. (F) Tumor weight. (G) Tumor growth curves. **H-J** The effects of *Rai14* overexpression in HCC cells on the growth of subcutaneous tumors (*n* = 6). (H) Representative. (I) Tumor weight. (J) Tumor growth curves. **K-M** The impact of *Rai14*-KO in HCC cells on carcinoma in situ growth (*n* = 6). Wild-type and *Rai14* knockout Hep-53.4 cells were implanted into the liver of mice, with observations made on the mice’s condition and survival time recorded. Upon the death of the mice, HCC tissues were collected for further analysis. (K) Representative. (L) Survival curves. (M) Tumor weight. **N-P** The effects of *Rai14* overexpression in HCC cells on carcinoma in situ progression (*n* = 6). (N) Representative. (O) Survival curves. (P) Tumor weight. **Q, R** Effect of overexpression of *Rnaseh2c* and knock out *Hsc70* (Q) or knockout of *Rnaseh2c* and *Cmtm6* (R) on the expression of RAI14 protein in RAW264.7 cells (*n* = 3). **S, T** Impact of *Rnaseh2c* (S) or *Traf3ip1* (T) -cKO on the expression of CMTM6 protein in mouse HCC-infiltrating macrophages (*n* = 4). **U** Immunoblotting demonstrating the impact of *Rnaseh2c* or *Traf3ip1*-cKI on the expression of HSC70 protein in mouse HCC-infiltrating macrophages (*n* = 3). **A, D, F, G, I, J, P, Q** represented mean ± SD analyzed by Wilcoxon test, **B, C, M, R-U** represented mean ± SD analyzed by unpaired *t* test. **P* < 0.05, ***P* < 0.01. cKI, conditional knock in; cKO, conditional knockout; HCC, hepatocellular carcinoma.


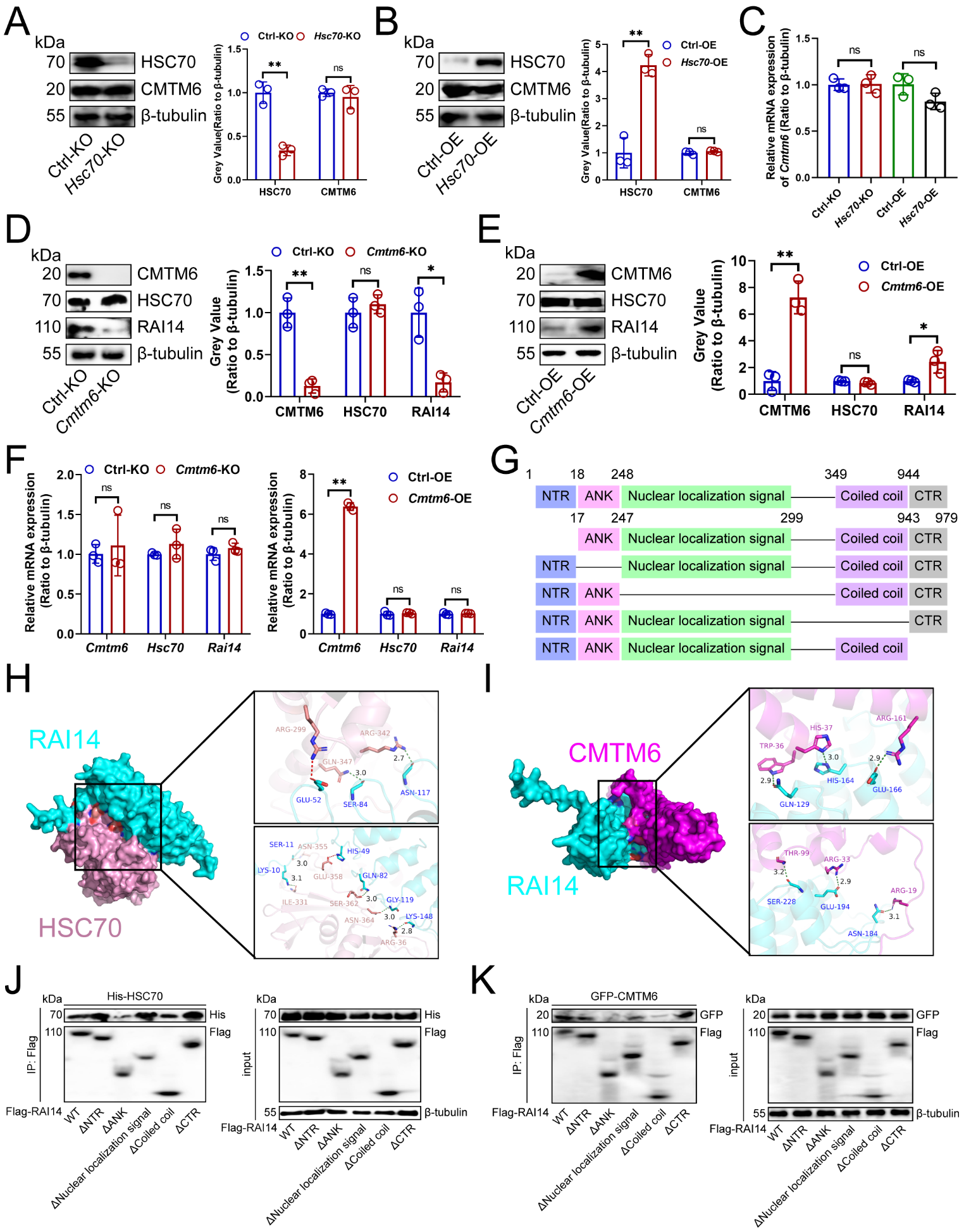


**Fig. S4** RAI14 degradation induced by CMTM6 depletion depended on HSC70. **A, B** Impact of knockout (A) or overexpression (B) of *Hsc70* on the expression of CMTM6 protein in RAW264.7 cells (*n* = 3). **C** Effect of knockout or overexpression of *Hsc70* on the expression of *Cmtm6* transcript in RAW264.7 cells (*n* = 3). **D, E** Impact of knockout (D) or overexpression (E) of *Cmtm6* on the expression of HSC70 and RAI14 proteins in RAW264.7 cells (*n* = 3). **F** Effect of knockout or overexpression of *Cmtm6* on the expression of *Hsc70* and *Rai14* transcripts in RAW264.7 cells (*n* = 3). **G** Schematic diagram of different truncation in mouse RAI14 protein. **H** Surface diagram showing the docking model and interfacing residues between mouse HSC70 and RAI14 proteins, highlighting hydrogen bonds with dotted lines. **I** Surface diagram demonstrating the docking model and interfacing residues between mouse CMTM6 and RAI14 proteins, highlighting hydrogen bonds with dotted lines. **J** RAW264.7 cells were transfected with Flag-labeled RAI14 and His-labeled HSC70, the interaction between RAI14 and HSC70 was analyzed by immunoprecipitation (*n* = 3). **K** RAW264.7 cells were transfected with Flag-labeled RAI14 and GFP-labeled CMTM6, the interaction between RAI14 and CMTM6 was analyzed by immunoprecipitation (*n* = 3). **A, B, D-F** represented mean ± SD analyzed by unpaired *t* test; **C** represented mean ± SD analyzed by Wilcoxon test. **P* < 0.05, ***P* < 0.01.


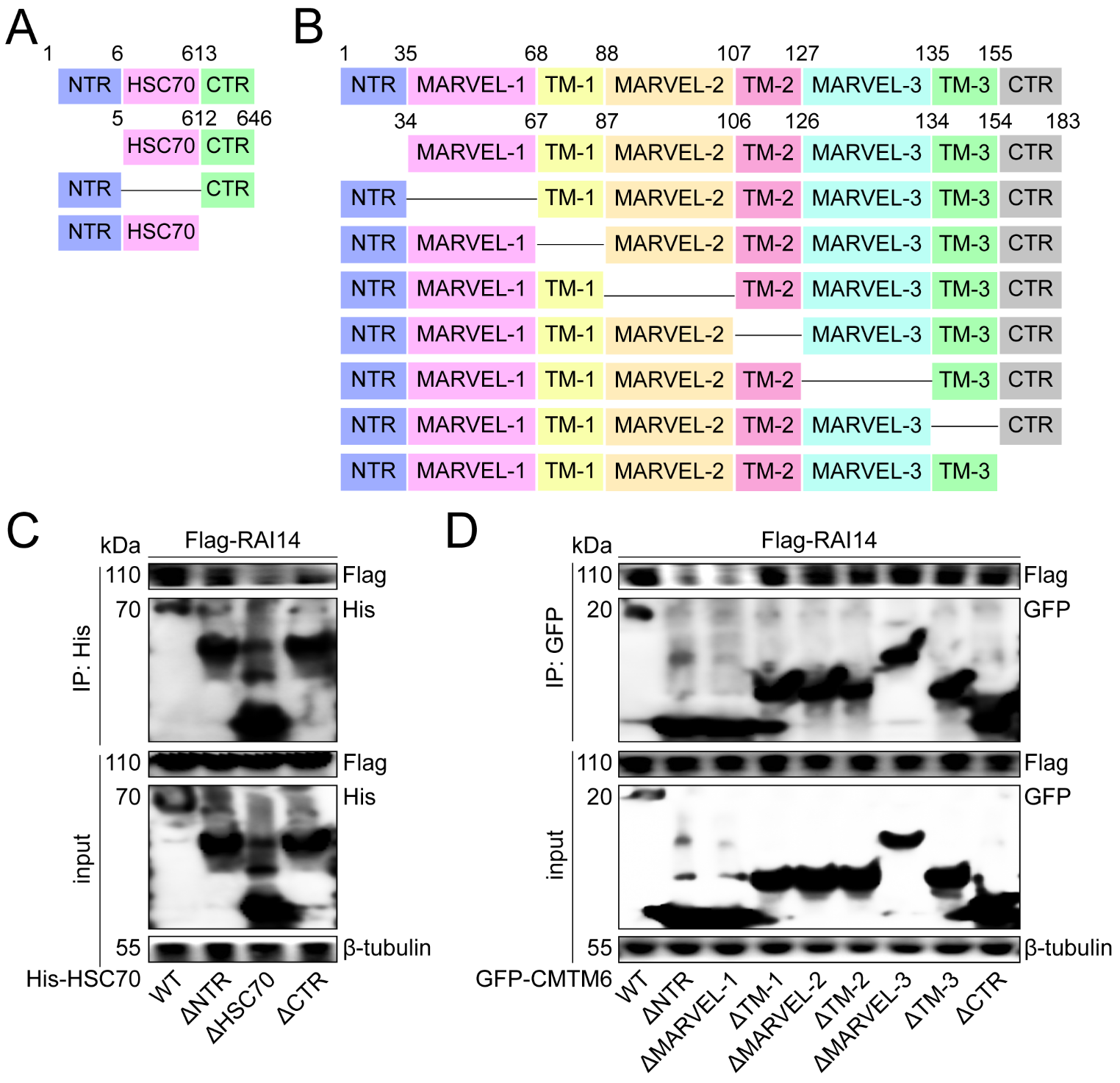


**Fig. S5 Identification of domain of HSC70 or CMTM6 binding to RAI14. A** Schematic diagram of different truncation in mouse HSC70 protein. **B** Schematic diagram of different truncation in mouse CMTM6 protein. **C** RAW264.7 cells were transfected with Flag-labeled RAI14 and His-labeled HSC70, the interaction between RAI14 and HSC70 was analyzed by immunoprecipitation (*n* = 3). **D** RAW264.7 cells were transfected with Flag-labeled RAI14 and GFP-labeled CMTM6, the interaction between RAI14 and CMTM6 was analyzed by immunoprecipitati on (*n* = 3).


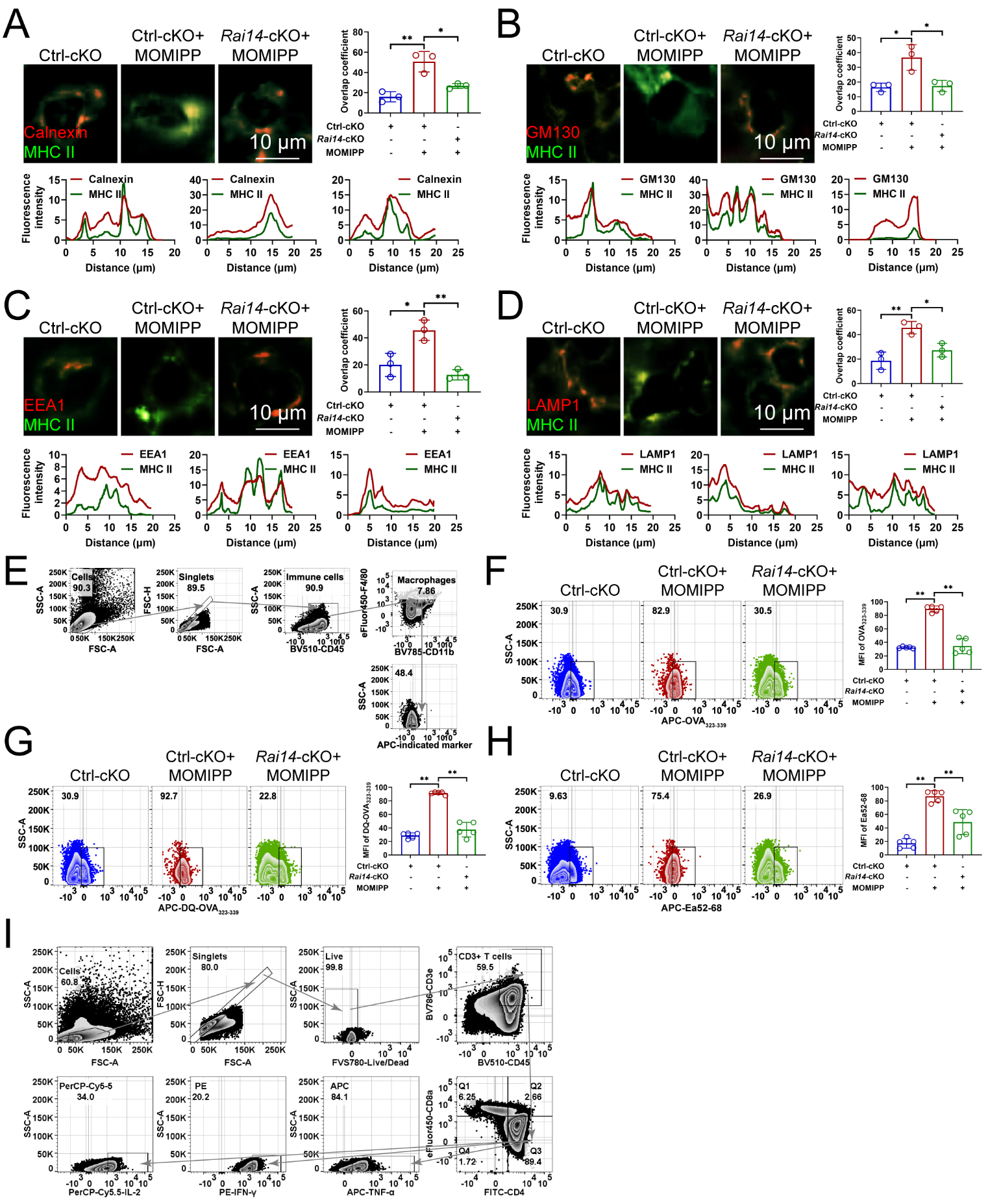


**Fig. S6** The influence of RAI14 on the antigen-presenting capacity of mouse macrophages infiltrating HCC. **A-D** The subcellular localization of MHC II molecules within RAW264.7 cells (*n* = 3). Specific markers, including Calnexin (A), GM130 (B), EEA1 (C), and LAMP1 (D), were utilized to identify the endoplasmic reticulum, Golgi apparatus, endosome, and lysosome, respectively. Wild-type and *Rai14*-cKO mice exhibiting primary HCC were stimulated with MOMIPP. Subsequently, macrophages were isolated from tumor tissues and immunized with the OVA_323-339_. The immunized macrophages were then co-cultured with OT-II T cells derived from OT-II mice for 3 d. Following this co-culture period, the macrophages were analyzed for the subcellular localization of specific markers using immunofluorescence. **E** The gating logic for identifying the expression of OVA_323-339_ or Ea52-68 peptides in macrophages by flow cytometry. Antigen internalization, degradation, and peptide loading on MHC II molecules were respectively labeled using APC-labeled OVA_323-339_, DQ-OVA_323-339_, and Ea52-68. **F** The expression of OVA_323-339_ in macrophages (*n* = 5). **G** The level of degraded OVA_323-339_ in macrophages through DQ-OVA (*n* = 5). **H** The expression of Ea52-68 peptide loaded onto MHC II molecules in macrophages (*n* = 5). **I** Flow cytometry gating logic relative to Fig. 7B. **A, B, F-H** represented mean ± SD analyzed by Wilcoxon test. **C, D** represented mean ± SD analyzed by unpaired *t* test. **P* < 0.05, ***P* < 0.01. HCC, hepatocellular carcinoma.


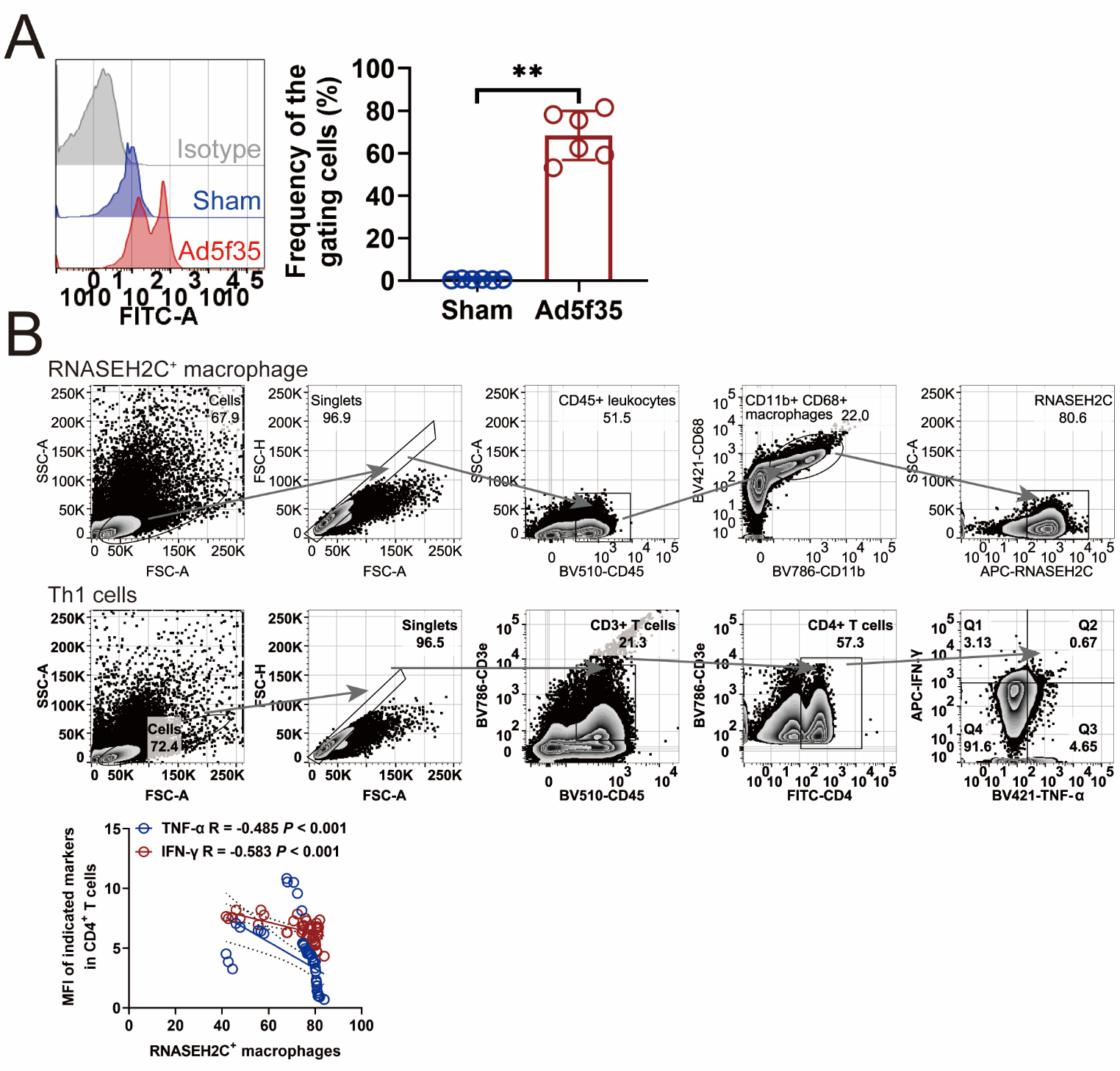


**Fig. S7** The clinical significance of RNASEH2C in HCC. **A** The positive rate of human macrophages infected with Ad5f35 (*n* = 6). Human macrophages were infected with the Ad5f35 adeno-associated virus, and the efficiency of infection was assessed using flow cytometry. **B** Flow cytometry demonstrating the correlation between the expression of RNASEH2C in the HCC-infiltrating macrophages of the patient and the content of Th1 cells in HCC (*n* = 49). **A** represented mean ± SD analyzed by Wilcoxon test. **B** was analyzed by Pearson correlation analysis. ***P* < 0.01. HCC, hepatocellular carcinoma.

**Supplementary Table**

**Table S1. Clinical data of hepatocellular carcinoma patients.**

| **Sample** | **Gender** | **Age** | **Pathological grading** | **Smoking** | **Alcoholism** | **Diabetes** |
| --- | --- | --- | --- | --- | --- | --- |
| Sample 1 | Male | 51 | IV | Yes | Yes | Yes |
| Sample 2 | Male | 46 | III | No | Yes | No |
| Sample 3 | Male | 63 | IV | Yes | No | Yes |
| Sample 4 | Male | 65 | II | Yes | No | Yes |
| Sample 5 | Female | 71 | II | Yes | No | Yes |
| Sample 6 | Male | 52 | I | Yes | No | Yes |
| Sample 7 | Female | 75 | III | No | Yes | Yes |
| Sample 8 | Female | 71 | IV | No | No | Yes |
| Sample 9 | Female | 56 | I | No | Yes | Yes |
| Sample 10 | Male | 72 | I | No | No | Yes |
| Sample 11 | Male | 69 | III | Yes | Yes | No |
| Sample 12 | Female | 46 | II | Yes | Yes | Yes |
| Sample 13 | Female | 61 | IV | Yes | No | Yes |
| Sample 14 | Male | 56 | III | Yes | No | Yes |
| Sample 15 | Female | 69 | I | Yes | No | Yes |
| Sample 16 | Male | 72 | III | No | Yes | No |
| Sample 17 | Female | 68 | IV | No | Yes | No |
| Sample 18 | Female | 54 | IV | Yes | No | No |
| Sample 19 | Female | 64 | IV | Yes | Yes | Yes |
| Sample 20 | Male | 71 | IV | No | Yes | No |
| Sample 21 | Male | 56 | II | No | Yes | No |
| Sample 22 | Male | 68 | II | Yes | Yes | Yes |
| Sample 23 | Male | 54 | III | No | Yes | Yes |
| Sample 24 | Male | 51 | IV | No | No | Yes |
| Sample 25 | Female | 62 | III | Yes | No | Yes |
| Sample 26 | Male | 54 | III | No | Yes | No |
| Sample 27 | Female | 63 | III | No | Yes | No |
| Sample 28 | Female | 54 | IV | No | Yes | No |
| Sample 29 | Male | 71 | I | No | Yes | Yes |
| Sample 30 | Female | 45 | II | No | No | No |
| Sample 31 | Female | 70 | III | No | Yes | No |
| Sample 32 | Male | 68 | I | Yes | No | Yes |
| Sample 33 | Female | 46 | II | No | No | No |
| Sample 34 | Female | 72 | III | Yes | No | Yes |
| Sample 35 | Female | 71 | III | No | No | No |
| Sample 36 | Female | 67 | IV | Yes | Yes | Yes |
| Sample 37 | Male | 48 | IV | No | No | No |
| Sample 38 | Female | 64 | I | No | Yes | Yes |
| Sample 39 | Female | 59 | II | Yes | No | No |
| Sample 40 | Female | 52 | IV | No | No | No |
| Sample 41 | Female | 67 | II | Yes | Yes | No |
| Sample 42 | Male | 51 | IV | No | Yes | Yes |
| Sample 43 | Male | 54 | II | No | Yes | No |
| Sample 44 | Female | 51 | I | No | Yes | No |
| Sample 45 | Female | 73 | I | No | Yes | No |
| Sample 46 | Male | 73 | I | No | No | Yes |
| Sample 47 | Female | 51 | I | No | Yes | No |
| Sample 48 | Male | 69 | I | No | Yes | No |
| Sample 49 | Male | 54 | II | Yes | No | Yes |

**Supplementary References**

1. Perez G, Barber GP, Benet-Pages A, Casper J, Clawson H, Diekhans M, et al. The UCSC Genome Browser database: 2025 update. Nucleic Acids Res. 2025;53(D1):D1243–d9.

2. Ginno PA, Lott PL, Christensen HC, Korf I, Chédin F. R-loop formation is a distinctive characteristic of unmethylated human CpG island promoters. Mol Cell. 2012;45(6):814–25.

3. Guan L, Wu S, Zhu Q, He X, Li X, Song G, et al. GPC3-targeted CAR-M cells exhibit potent antitumor activity against hepatocellular carcinoma. Biochem Biophys Rep. 2024;39:101741.

4. Jiang P, Gu S, Pan D, Fu J, Sahu A, Hu X, et al. Signatures of T cell dysfunction and exclusion predict cancer immunotherapy response. Nat Med. 2018;24(10):1550–8.

5. Ay F, Kellis M, Kahveci T. SubMAP: aligning metabolic pathways with subnetwork mappings. J Comput Biol. 2011;18(3):219–35.

6. Yang Y, McBride KM, Hensley S, Lu Y, Chedin F, Bedford MT. Arginine methylation facilitates the recruitment of TOP3B to chromatin to prevent R loop accumulation. Mol Cell. 2014;53(3):484–97.

7. Hao Y, Stuart T, Kowalski MH, Choudhary S, Hoffman P, Hartman A, et al. Dictionary learning for integrative, multimodal and scalable single-cell analysis. Nat Biotechnol. 2024;42(2):293–304.

8. Stoeckius M, Zheng S, Houck-Loomis B, Hao S, Yeung BZ, Mauck WM, 3rd, et al. Cell Hashing with barcoded antibodies enables multiplexing and doublet detection for single cell genomics. Genome Biol. 2018;19(1):224.

9. Becht E, McInnes L, Healy J, Dutertre CA, Kwok IWH, Ng LG, et al. Dimensionality reduction for visualizing single-cell data using UMAP. Nat Biotechnol. 2018.

10. Aran D, Looney AP, Liu L, Wu E, Fong V, Hsu A, et al. Reference-based analysis of lung single-cell sequencing reveals a transitional profibrotic macrophage. Nat Immunol. 2019;20(2):163–72.

11. Bravo González-Blas C, De Winter S, Hulselmans G, Hecker N, Matetovici I, Christiaens V, et al. SCENIC+: single-cell multiomic inference of enhancers and gene regulatory networks. Nat Methods. 2023;20(9):1355–67.

12. Moerman T, Aibar Santos S, Bravo González-Blas C, Simm J, Moreau Y, Aerts J, et al. GRNBoost2 and Arboreto: efficient and scalable inference of gene regulatory networks. Bioinformatics. 2019;35(12):2159–61.

13. Nyberg WA, Ark J, To A, Clouden S, Reeder G, Muldoon JJ, et al. An evolved AAV variant enables efficient genetic engineering of murine T cells. Cell. 2023;186(2):446–60.e19.

14. Laskowski RA, Swindells MB. LigPlot+: multiple ligand-protein interaction diagrams for drug discovery. J Chem Inf Model. 2011;51(10):2778–86.

15. Kanehisa M, Goto S. KEGG: kyoto encyclopedia of genes and genomes. Nucleic Acids Res. 2000;28(1):27–30.

16. Love MI, Huber W, Anders S. Moderated estimation of fold change and dispersion for RNA-seq data with DESeq2. Genome Biol. 2014;15(12):550.
