# Supplementary material for: RNASEH2C enhances TRAF3IP1 to degrade RAI14 in lysosomes thus hindering macrophage antigen presentation and advancing liver cancer
Source: Cell Death Dis. 2025 Dec 8;17(1):92. doi: 10.1038/s41419-025-08305-5 (PMC12830818; doi:10.1038/s41419-025-08305-5)
Supplement: Supplementary file 2 — Raw blot [file 41419_2025_8305_MOESM2_ESM.pdf]

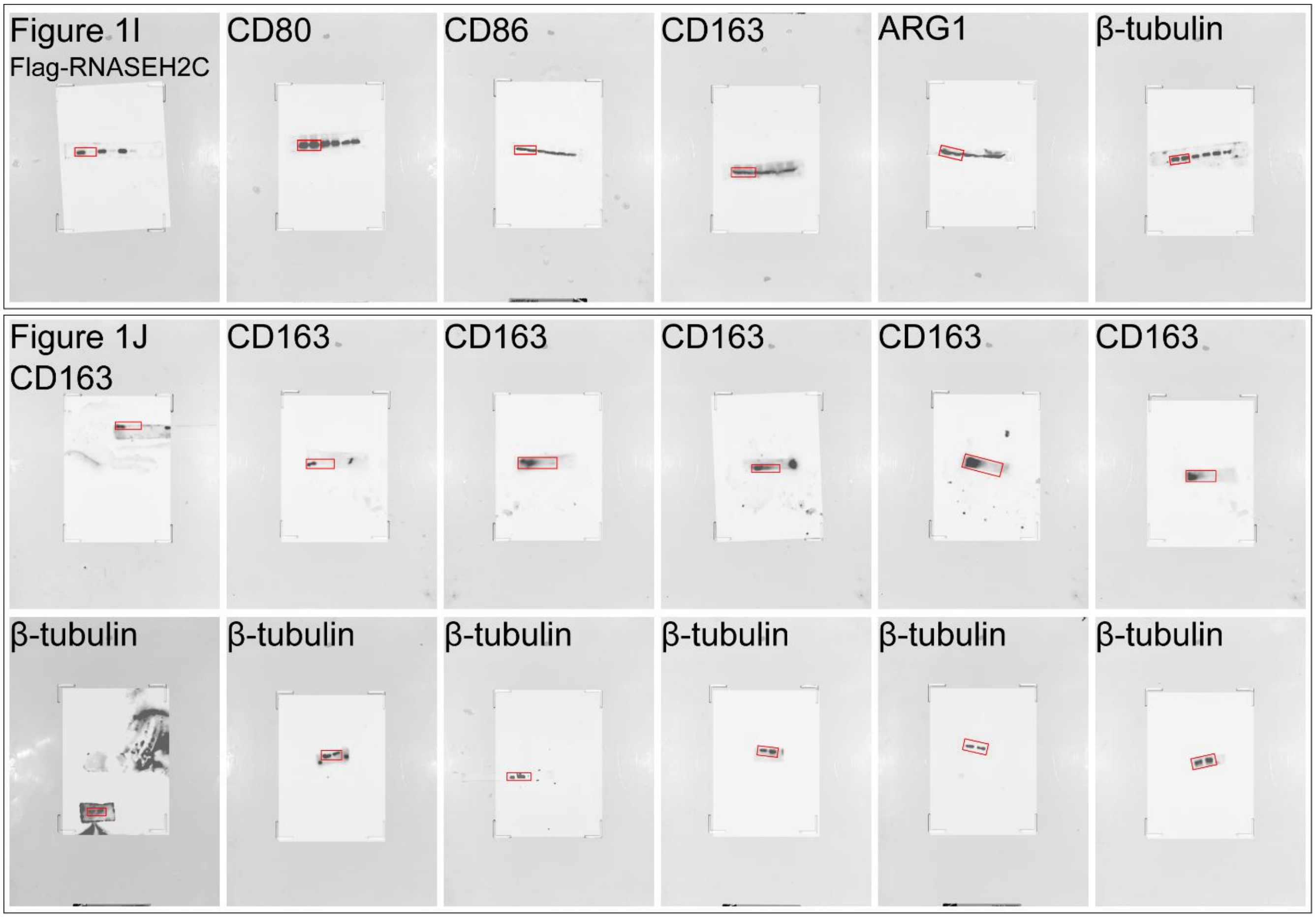
Figure 1I
Flag-RNASEH2C
CD80
CD86
CD163
ARG1
β-tubulin
Figure 1J
CD163
CD163
CD163
CD163
CD163
CD163
β-tubulin
β-tubulin
β-tubulin
β-tubulin
β-tubulin
β-tubulin

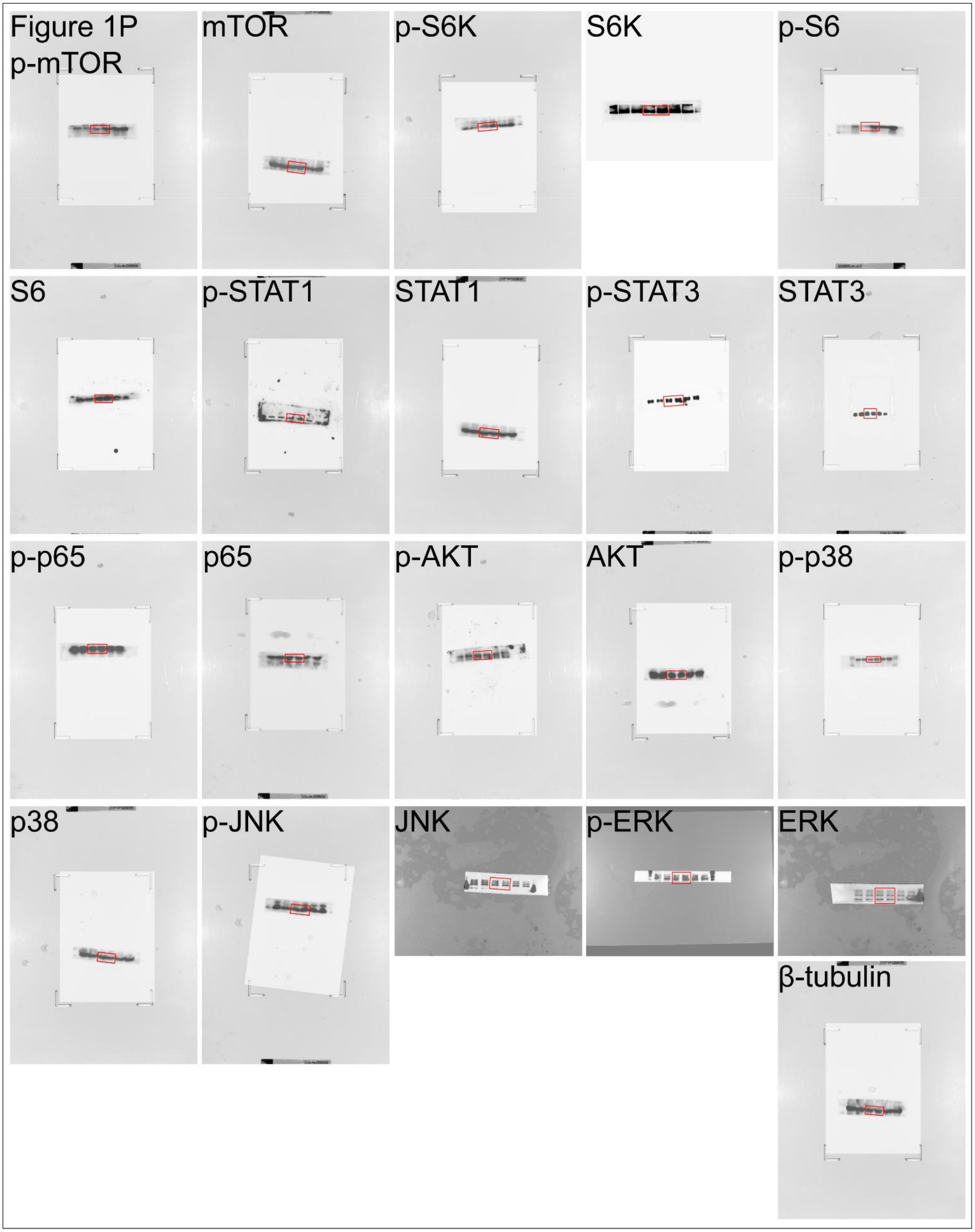
Figure 1P
p-mTOR
mTOR
p-S6K
S6K
p-S6
S6
p-STAT1
STAT1
p-STAT3
STAT3
p-p65
p65
p-AKT
AKT
p-p38
p38
p-JNK
JNK
p-ERK
ERK
β-tubulin

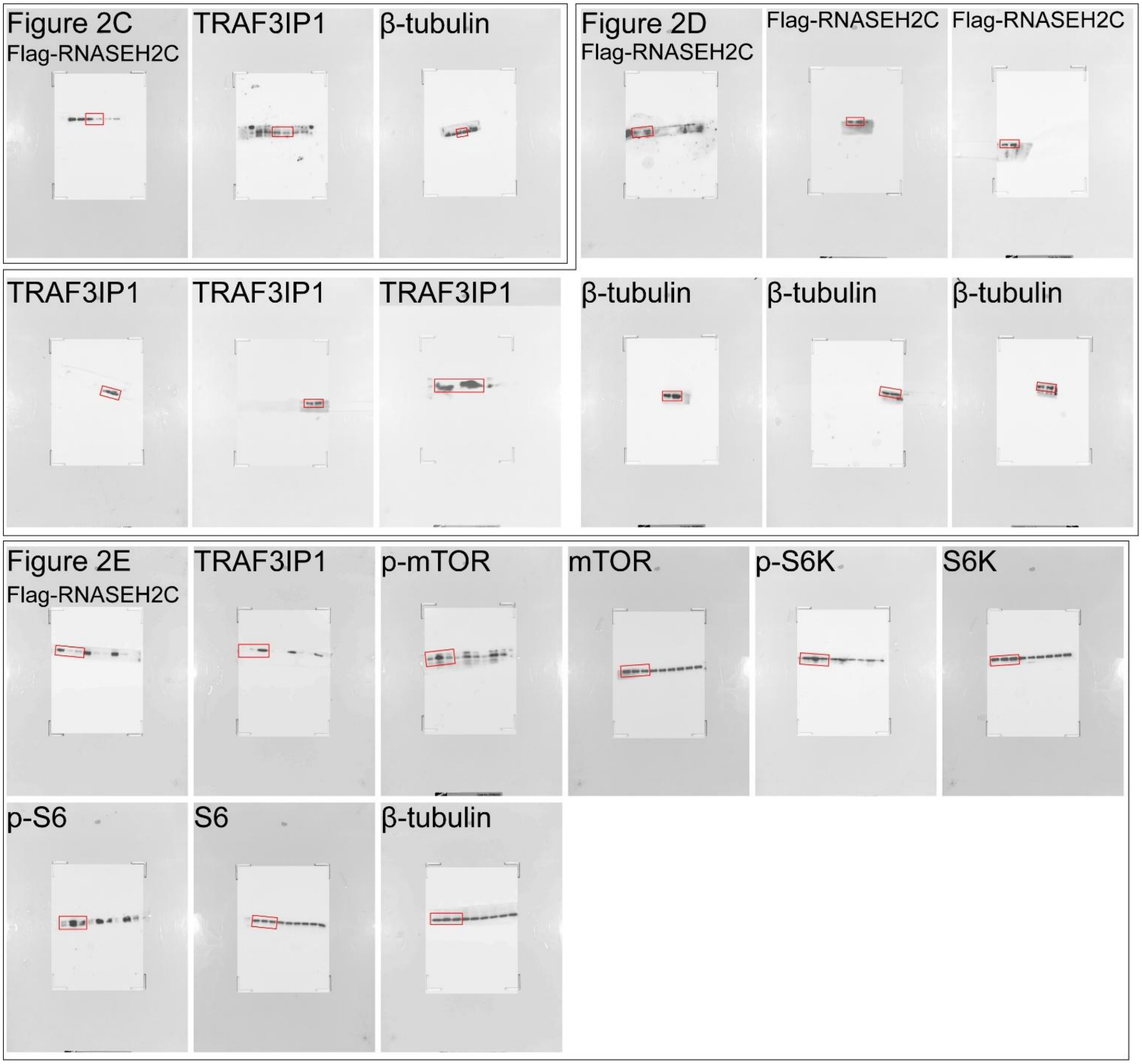
Figure 2C
Flag-RNASEH2C
TRAF3IP1
β-tubulin
Figure 2D
Flag-RNASEH2C
Flag-RNASEH2C
Flag-RNASEH2C
TRAF3IP1
TRAF3IP1
TRAF3IP1
β-tubulin
β-tubulin
β-tubulin
Figure 2E
Flag-RNASEH2C
TRAF3IP1
p-mTOR
mTOR
p-S6K
S6K
p-S6
S6
β-tubulin

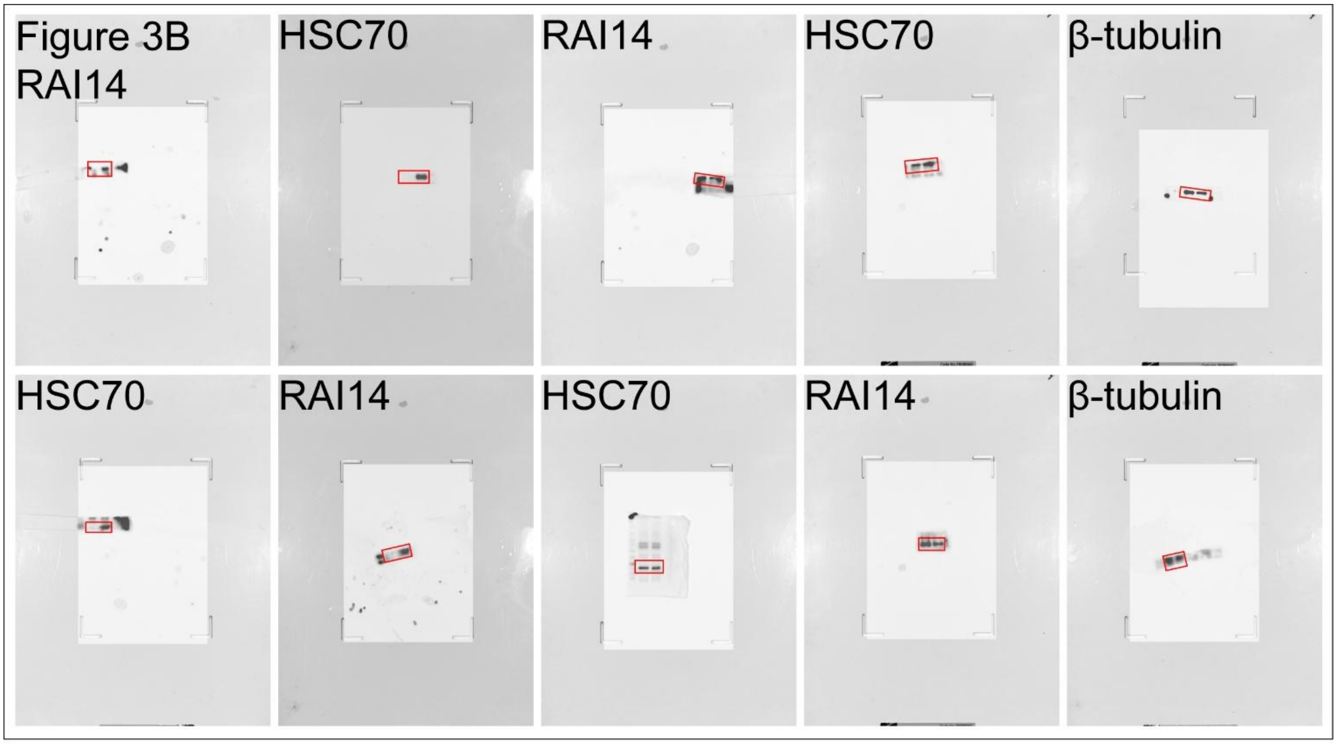
Figure 3B
RAI14
HSC70
RAI14
HSC70
β-tubulin
HSC70
RAI14
HSC70
RAI14
β-tubulin

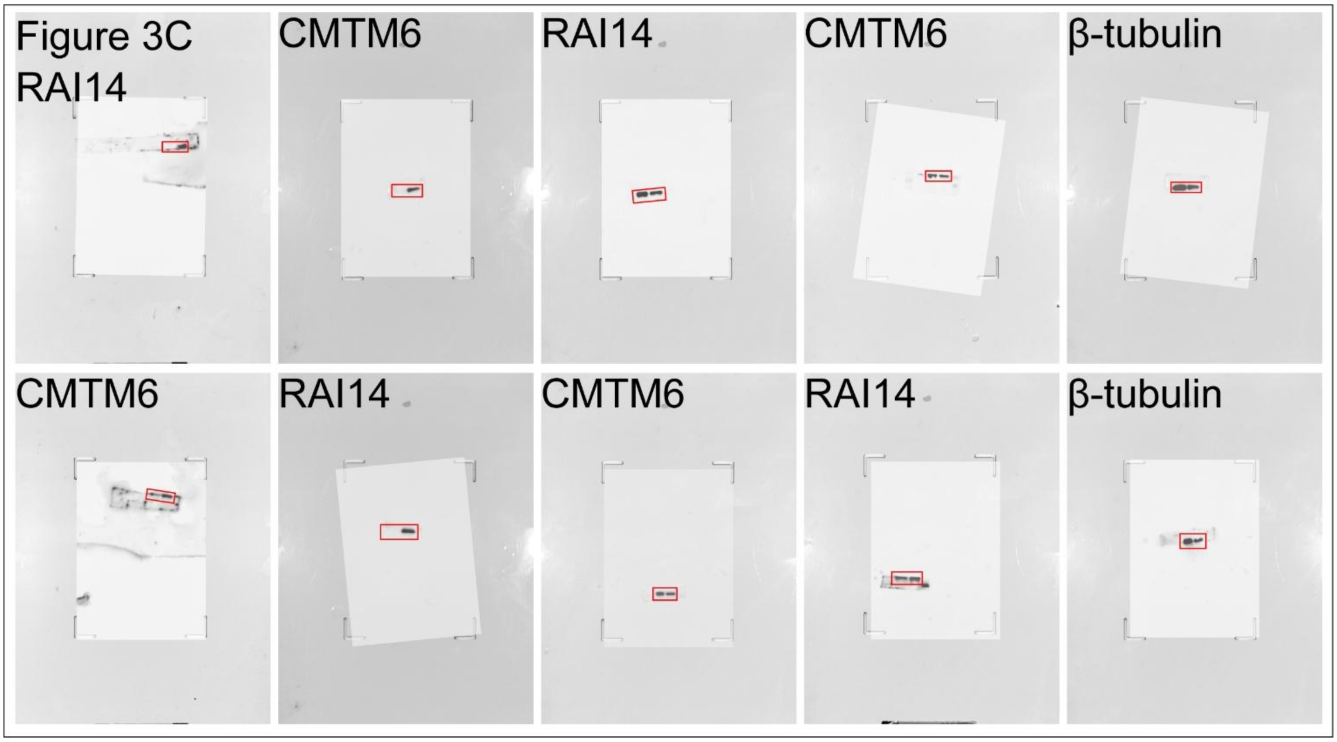
Figure 3C
RAI14
CMTM6
RAI14
CMTM6
β-tubulin
CMTM6
RAI14
CMTM6
RAI14
β-tubulin

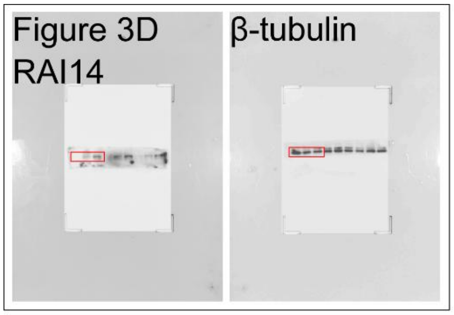
Figure 3D
RAI14
β-tubulin

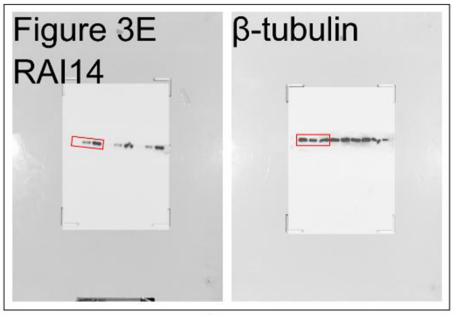
Figure 3E
RAI14
β-tubulin

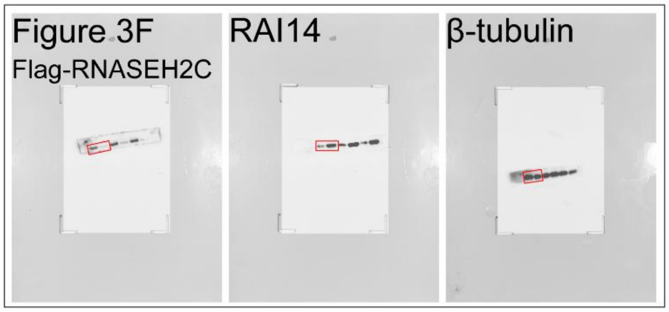
Figure 3F
Flag-RNASEH2C
RAI14
β-tubulin

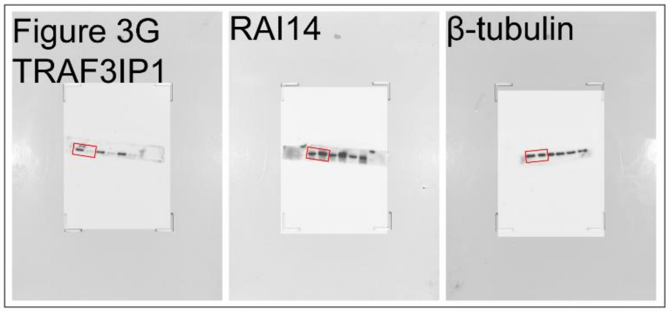
Figure 3G
TRAF3IP1
RAI14
β-tubulin

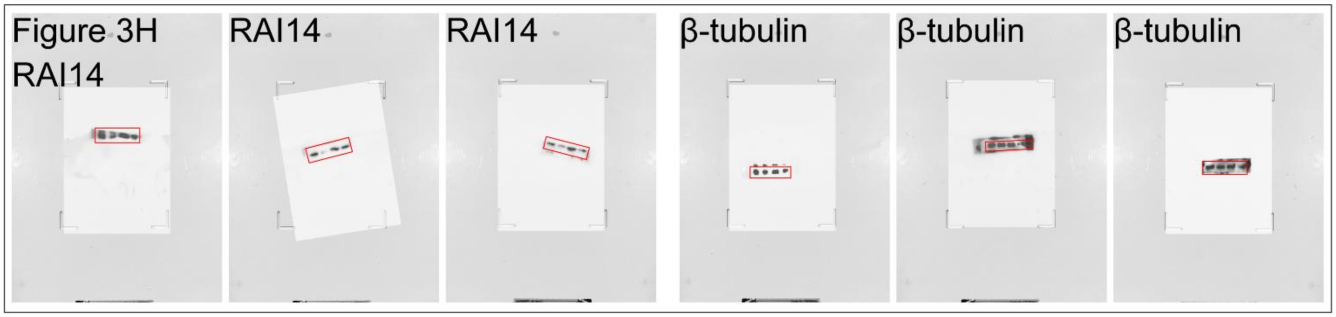
Figure 3H
RAI14
RAI14
RAI14
β-tubulin
β-tubulin
β-tubulin

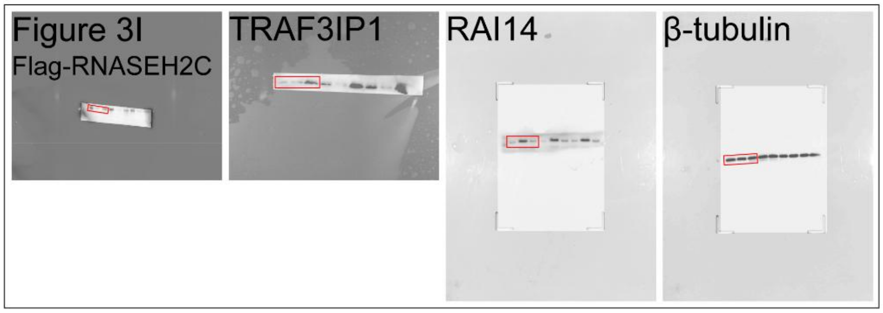
Figure 3I
Flag-RNASEH2C
TRAF3IP1
RAI14
β-tubulin

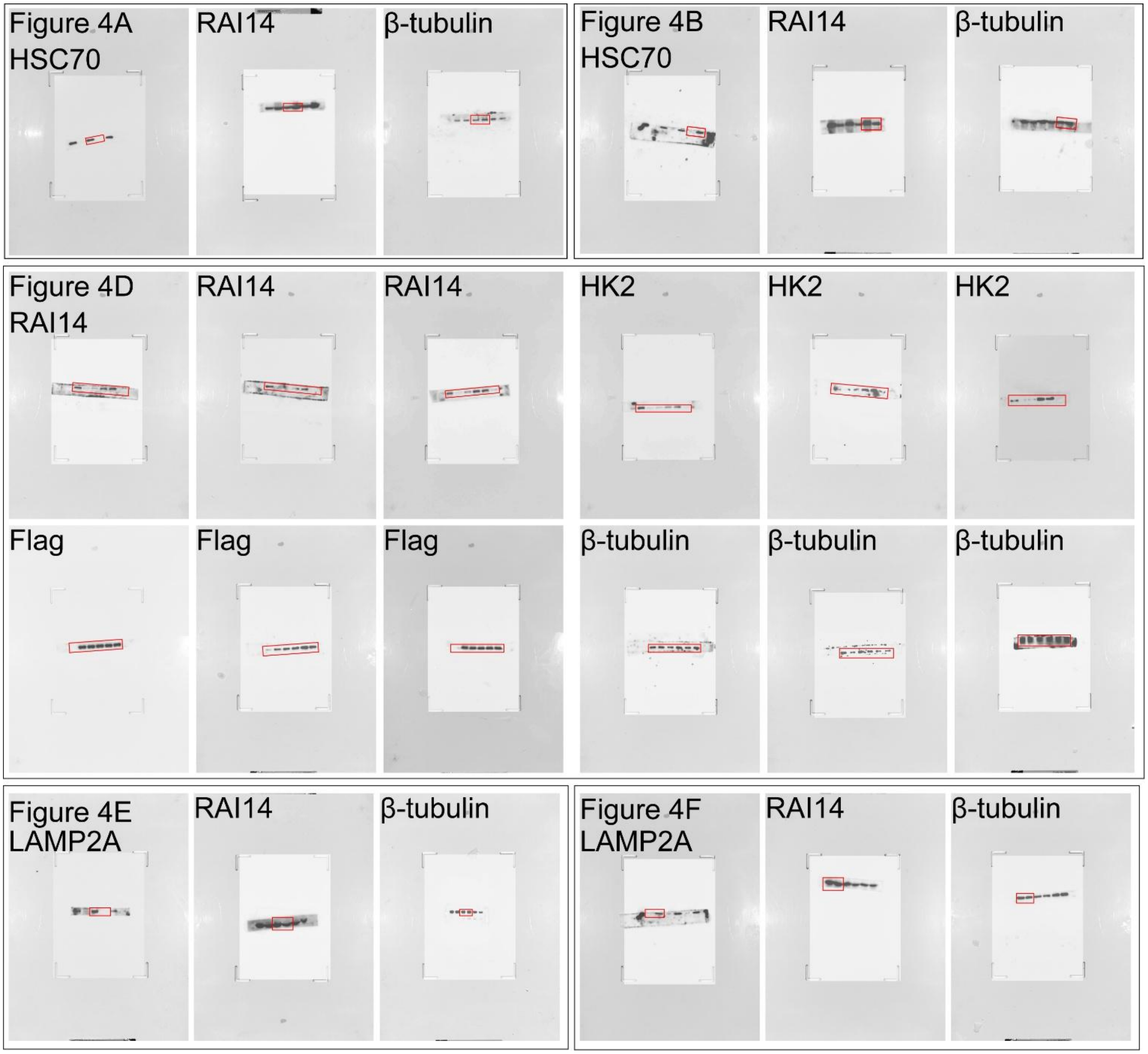
Figure 4A
HSC70
RAI14
β-tubulin
Figure 4B
HSC70
RAI14
β-tubulin
Figure 4D
RAI14
RAI14
RAI14
HK2
HK2
HK2
Flag
Flag
Flag
β-tubulin
β-tubulin
β-tubulin
Figure 4E
LAMP2A
RAI14
β-tubulin
Figure 4F
LAMP2A
RAI14
β-tubulin

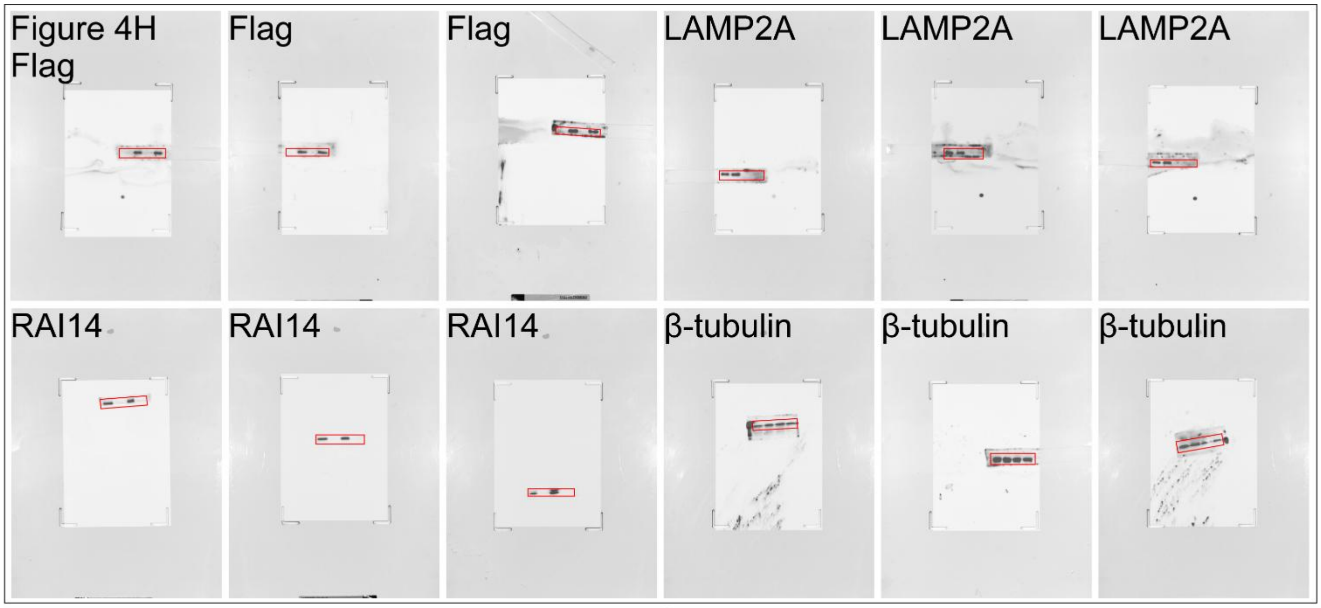
Figure 4H
Flag
Flag
Flag
LAMP2A
LAMP2A
LAMP2A
RAI14
RAI14
RAI14
β-tubulin
β-tubulin
β-tubulin

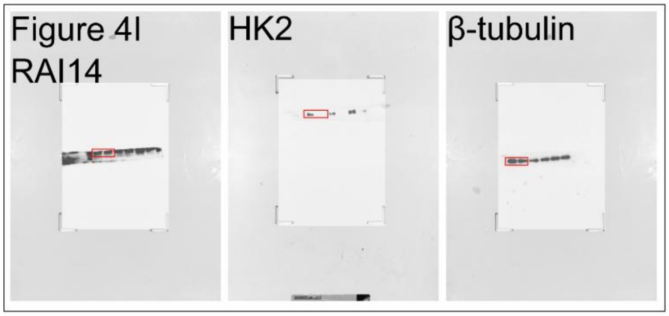
Figure 4I
RAI14
HK2
β-tubulin

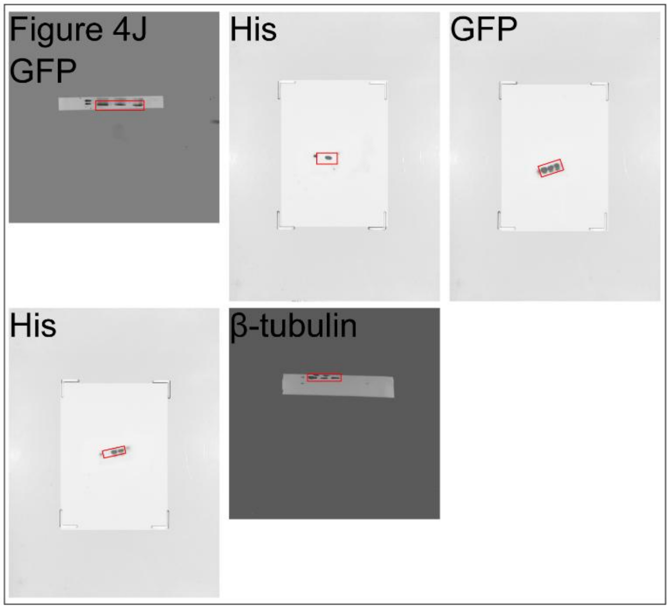
Figure 4J
GFP
His
GFP
His
β-tubulin

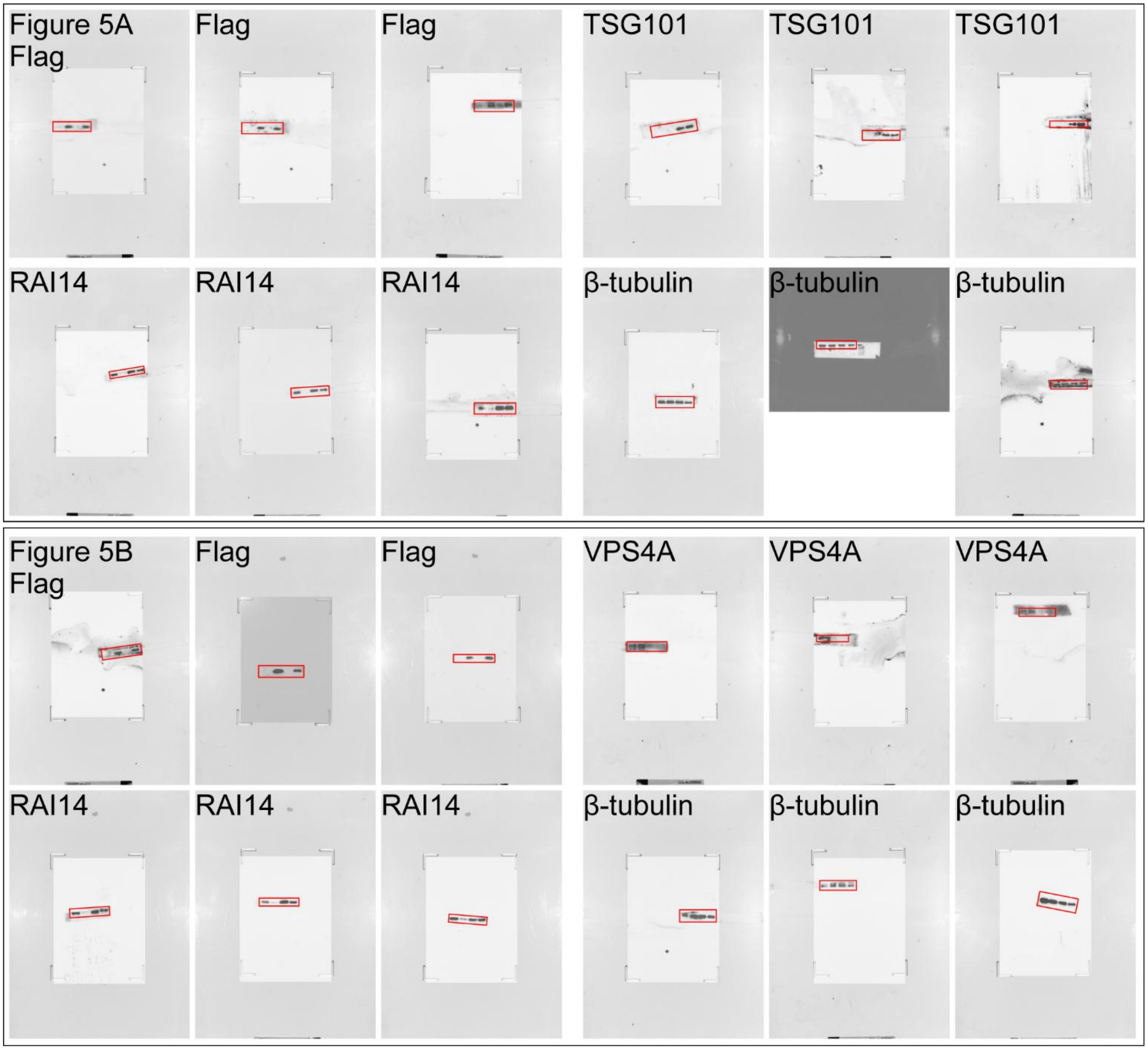
Figure 5A
Flag
Flag
Flag
TSG101
TSG101
TSG101
RAI14
RAI14
RAI14
β-tubulin
β-tubulin
β-tubulin
Figure 5B
Flag
Flag
Flag
VPS4A
VPS4A
VPS4A
RAI14
RAI14
RAI14
β-tubulin
β-tubulin
β-tubulin

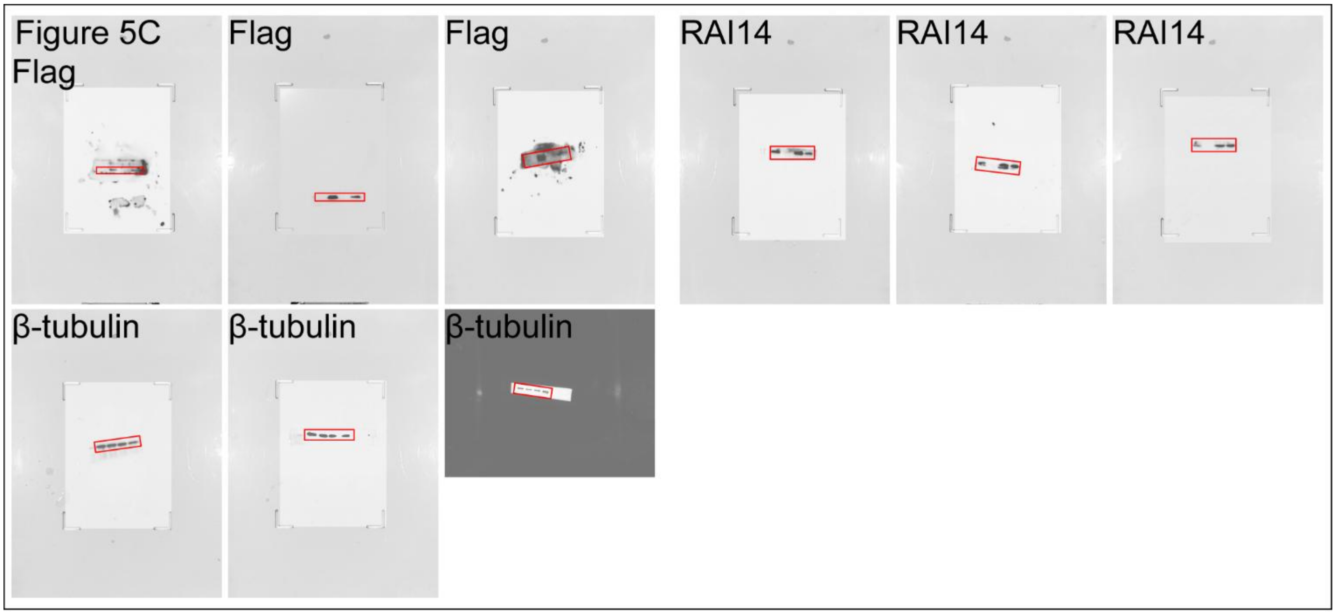
Figure 5C
Flag
Flag
Flag
RAI14
RAI14
RAI14
β-tubulin
β-tubulin
β-tubulin

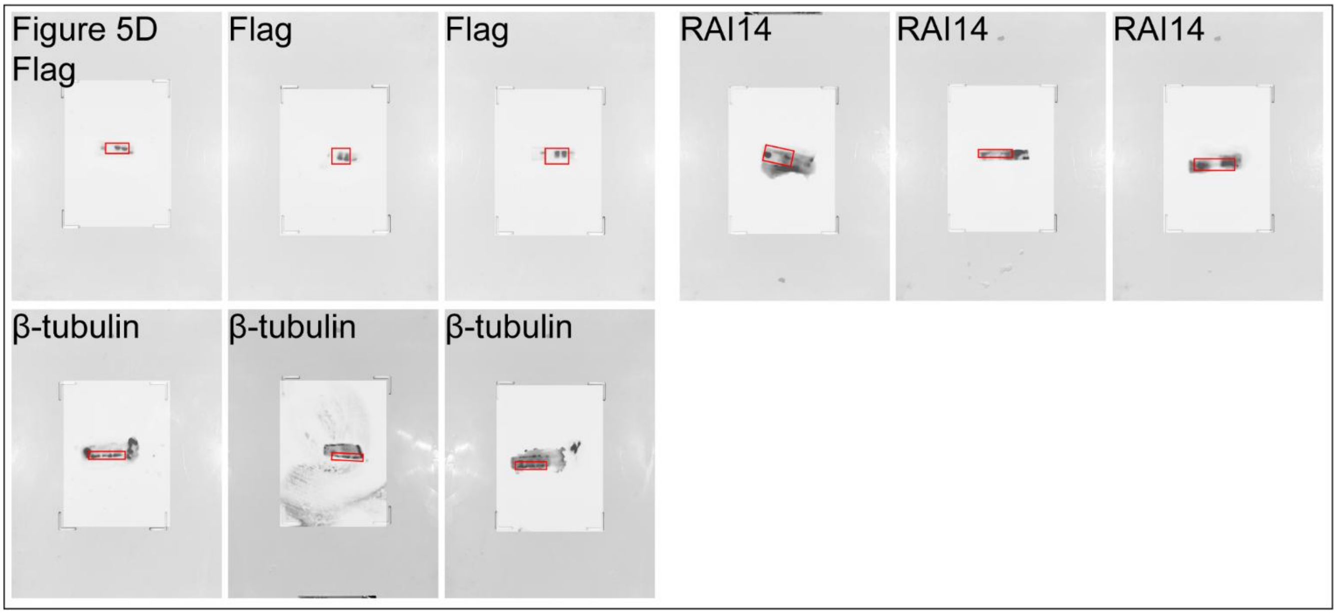
Figure 5D
Flag
Flag
Flag
RAI14
RAI14
RAI14
β-tubulin
β-tubulin
β-tubulin

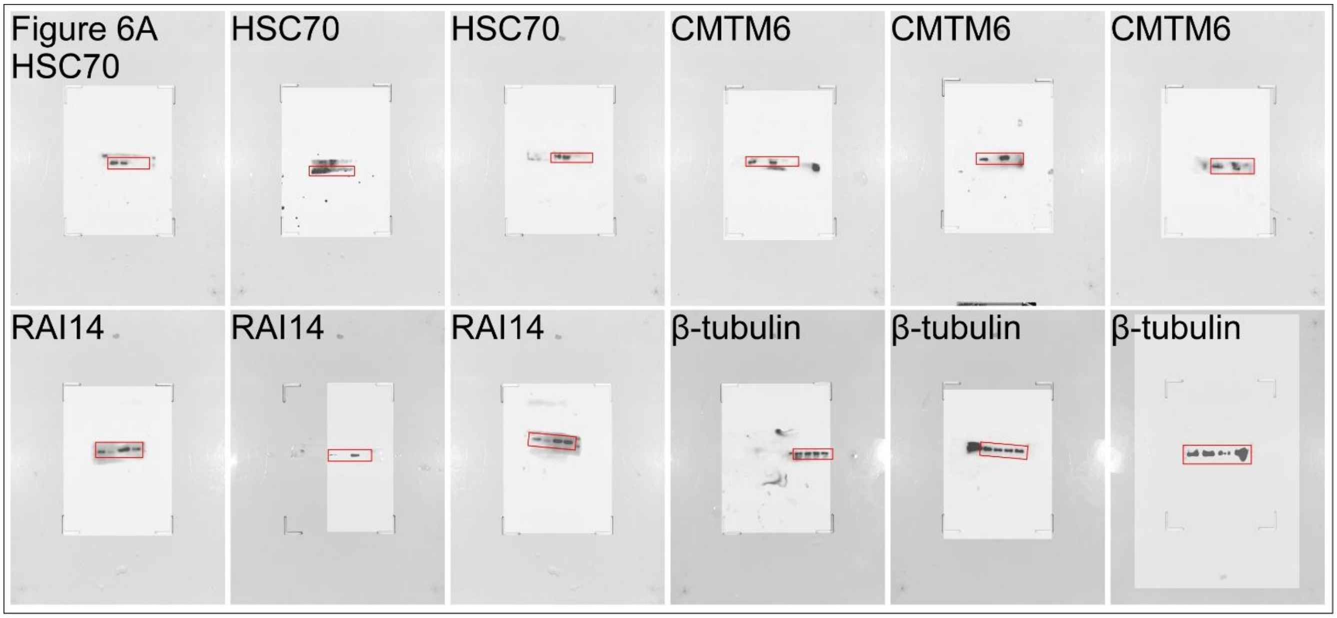
Figure 6A
HSC70
HSC70
HSC70
CMTM6
CMTM6
CMTM6
RAI14
RAI14
RAI14
β-tubulin
β-tubulin
β-tubulin

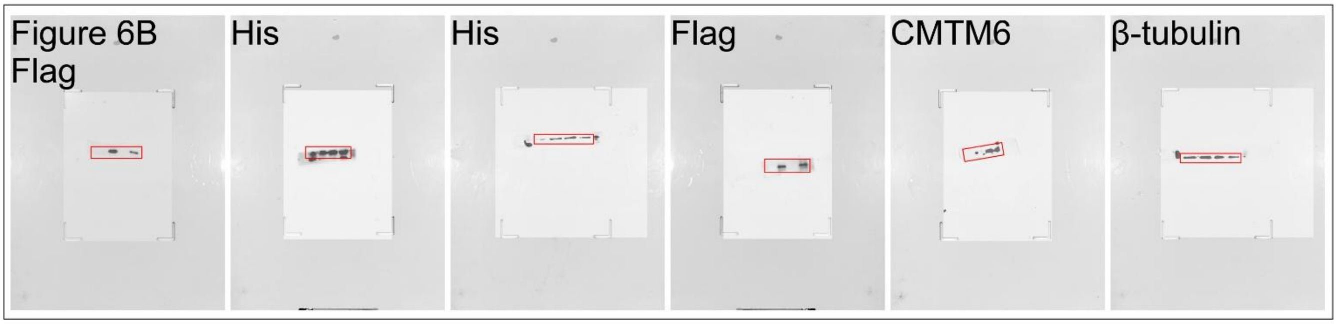
Figure 6B
Flag
His
His
Flag
CMTM6
β-tubulin

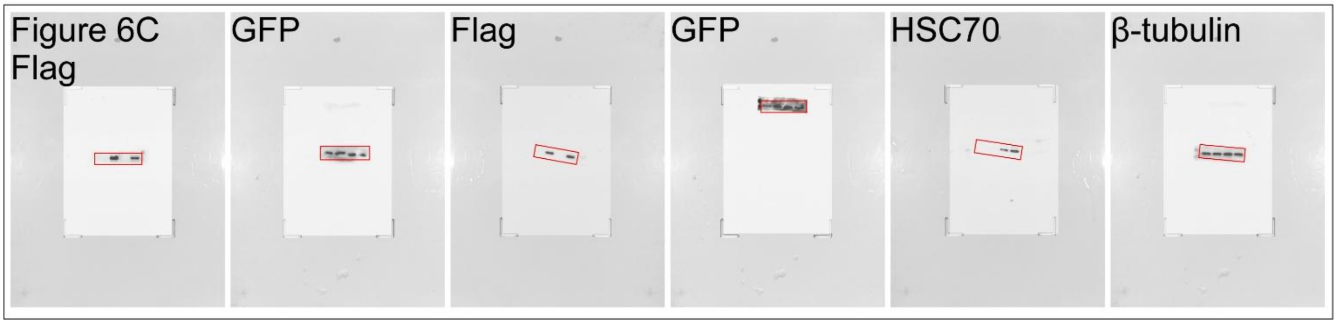
Figure 6C
Flag
GFP
Flag
GFP
HSC70
β-tubulin

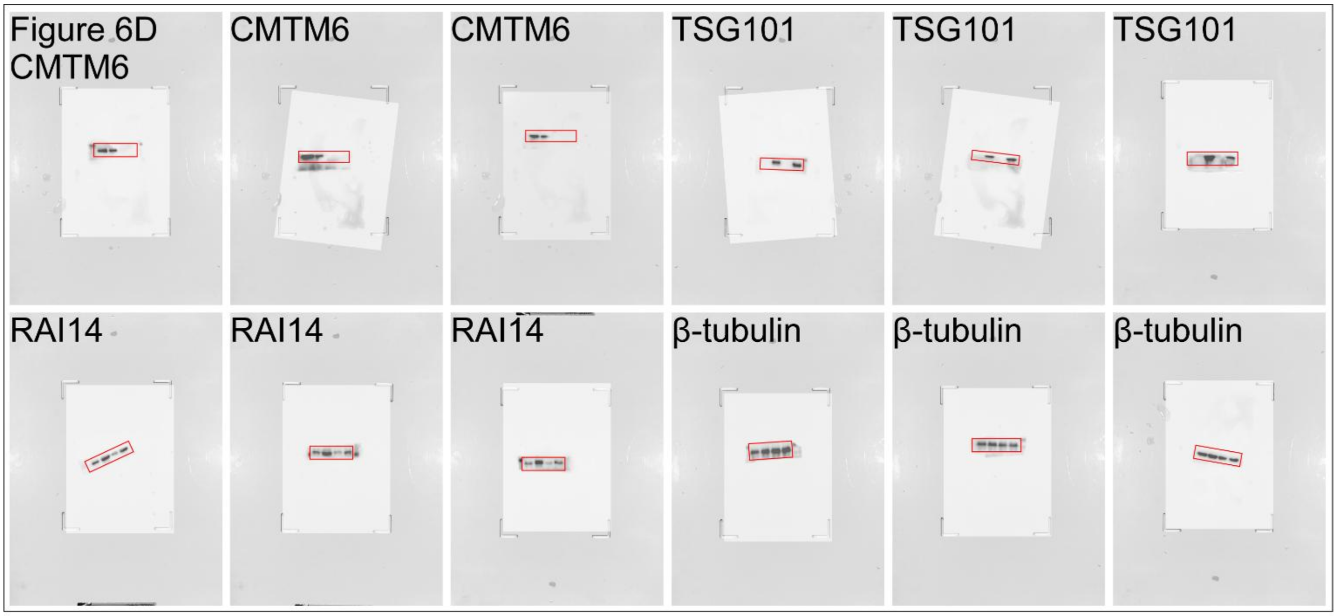
Figure 6D
CMTM6
CMTM6
CMTM6
TSG101
TSG101
TSG101
RAI14
RAI14
RAI14
β-tubulin
β-tubulin
β-tubulin

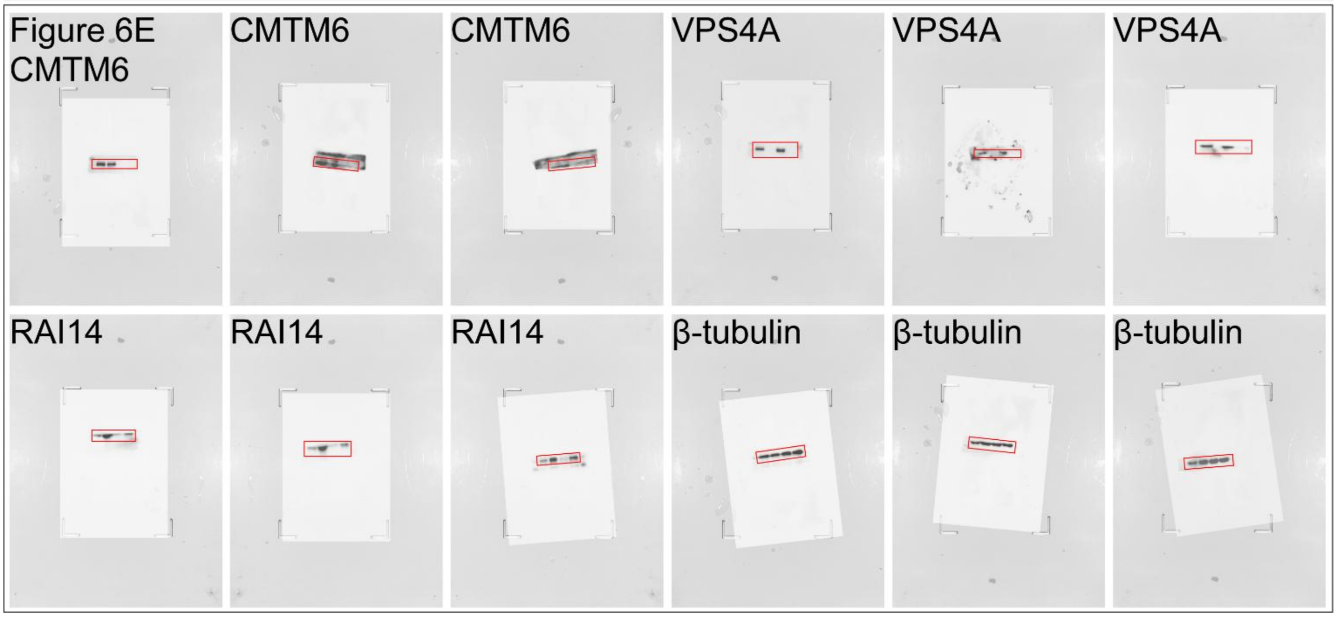
Figure 6E
CMTM6
CMTM6
CMTM6
VPS4A
VPS4A
VPS4A
RAI14
RAI14
RAI14
β-tubulin
β-tubulin
β-tubulin

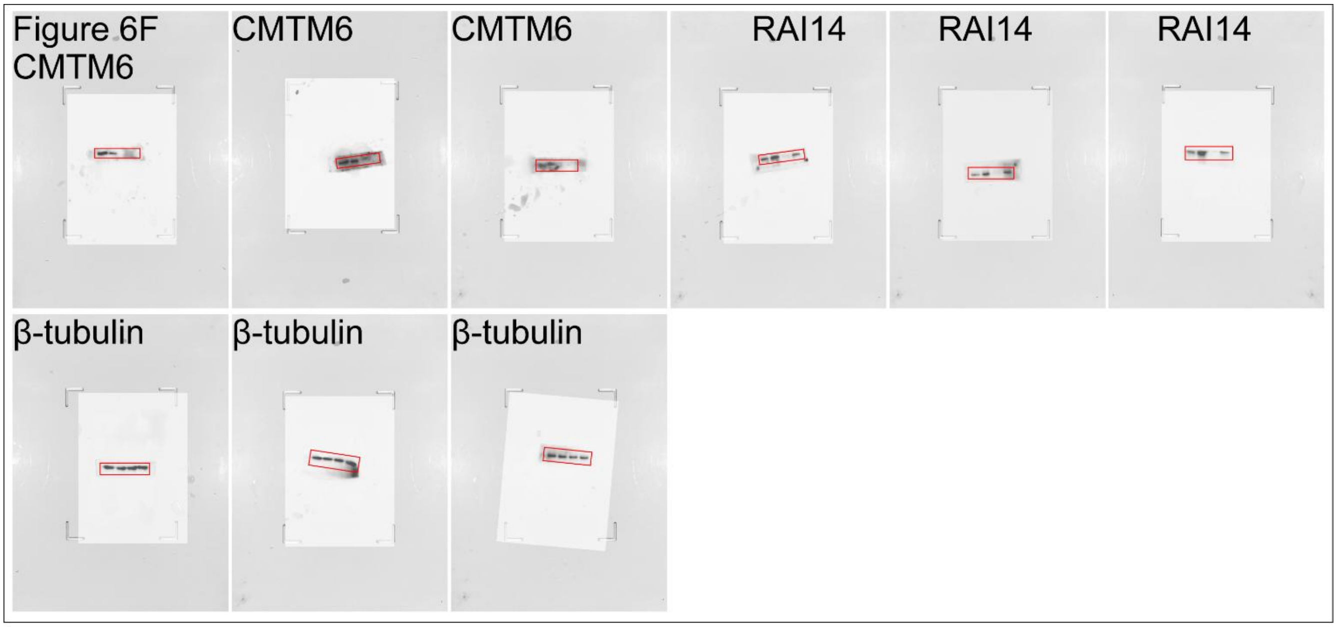
Figure 6F
CMTM6
CMTM6
CMTM6
RAI14
RAI14
RAI14
β-tubulin
β-tubulin
β-tubulin

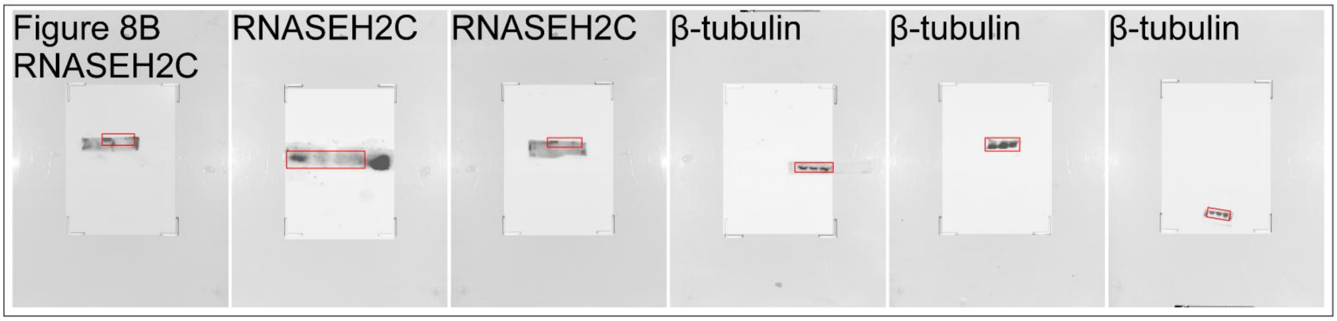
Figure 8B
RNASEH2C
RNASEH2C
RNASEH2C
β-tubulin
β-tubulin
β-tubulin

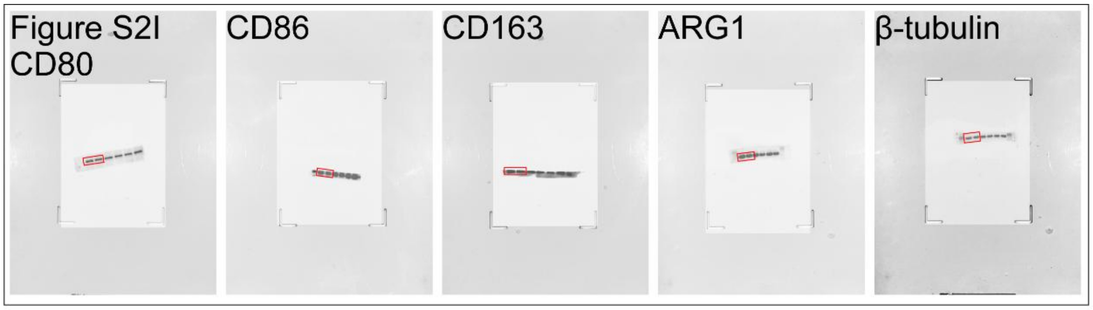
Figure S2I
CD80
CD86
CD163
ARG1
β-tubulin

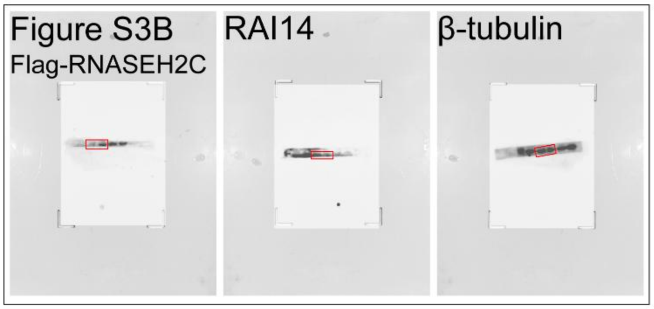
Figure S3B
Flag-RNASEH2C
RAI14
β-tubulin

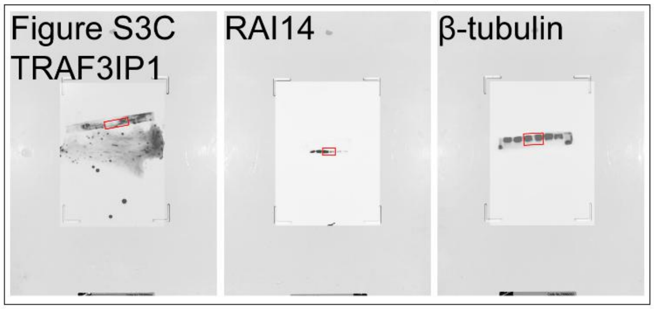
Figure S3C
TRAF3IP1
RAI14
β-tubulin

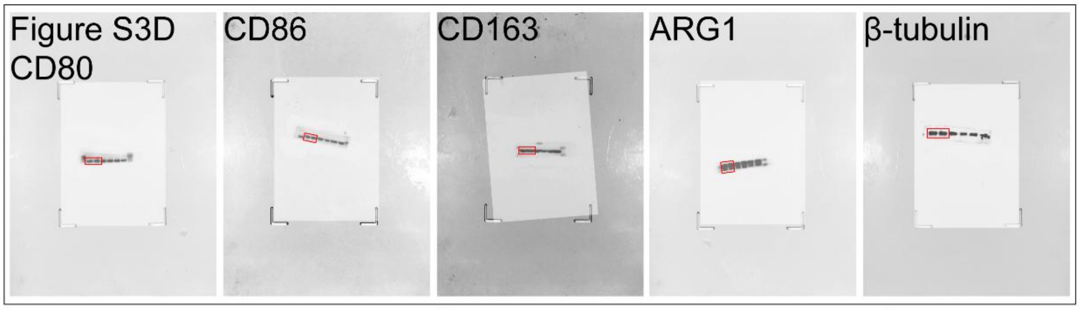
Figure S3D
CD80
CD86
CD163
ARG1
β-tubulin

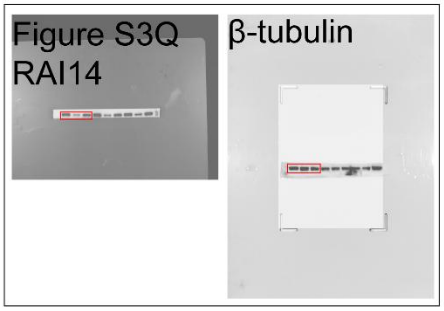
Figure S3Q
RAI14
β-tubulin

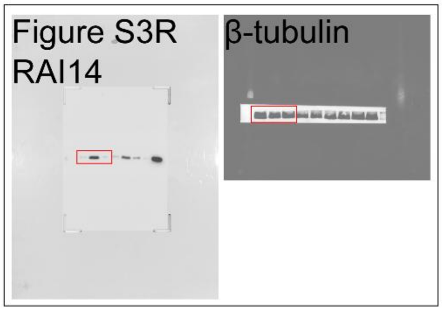
Figure S3R
RAI14
β-tubulin

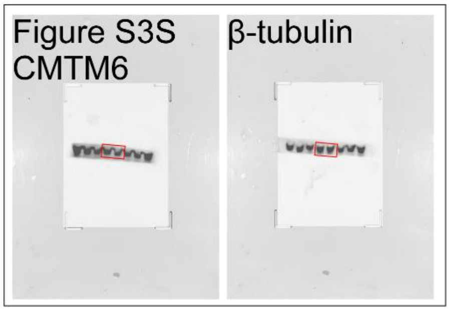
Figure S3S
CMTM6
β-tubulin

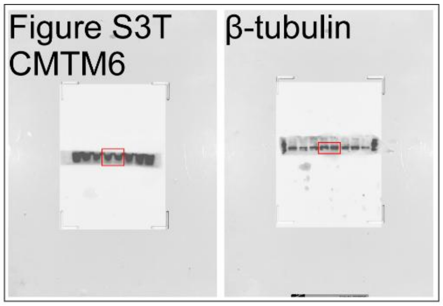
Figure S3T
CMTM6
β-tubulin

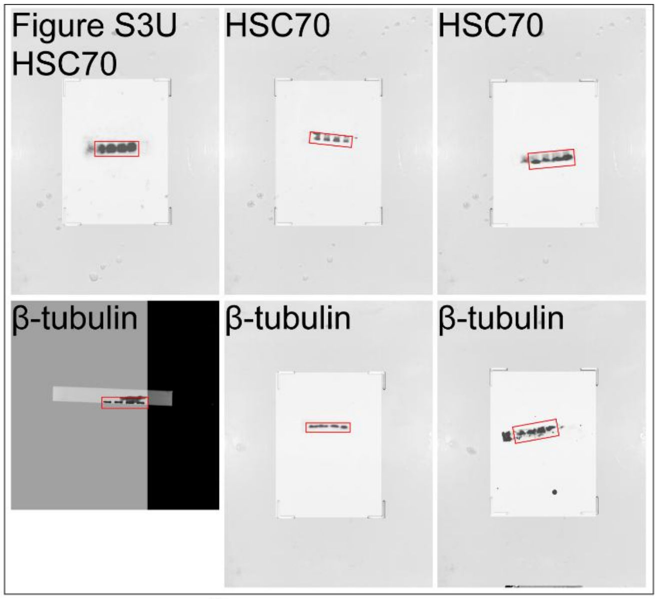
Figure S3U
HSC70
HSC70
HSC70
β-tubulin
β-tubulin
β-tubulin

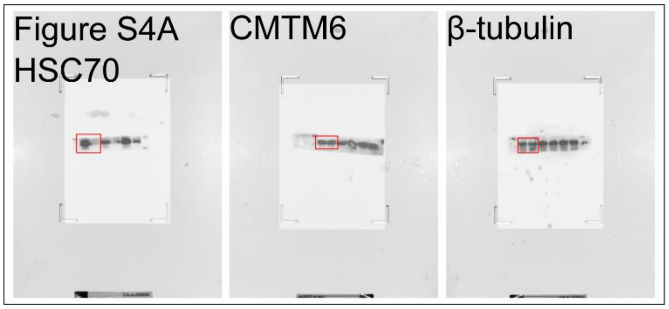
Figure S4A
HSC70
CMTM6
β-tubulin

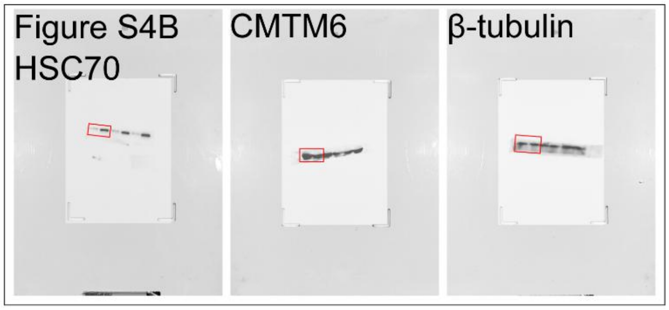
Figure S4B
HSC70
CMTM6
β-tubulin

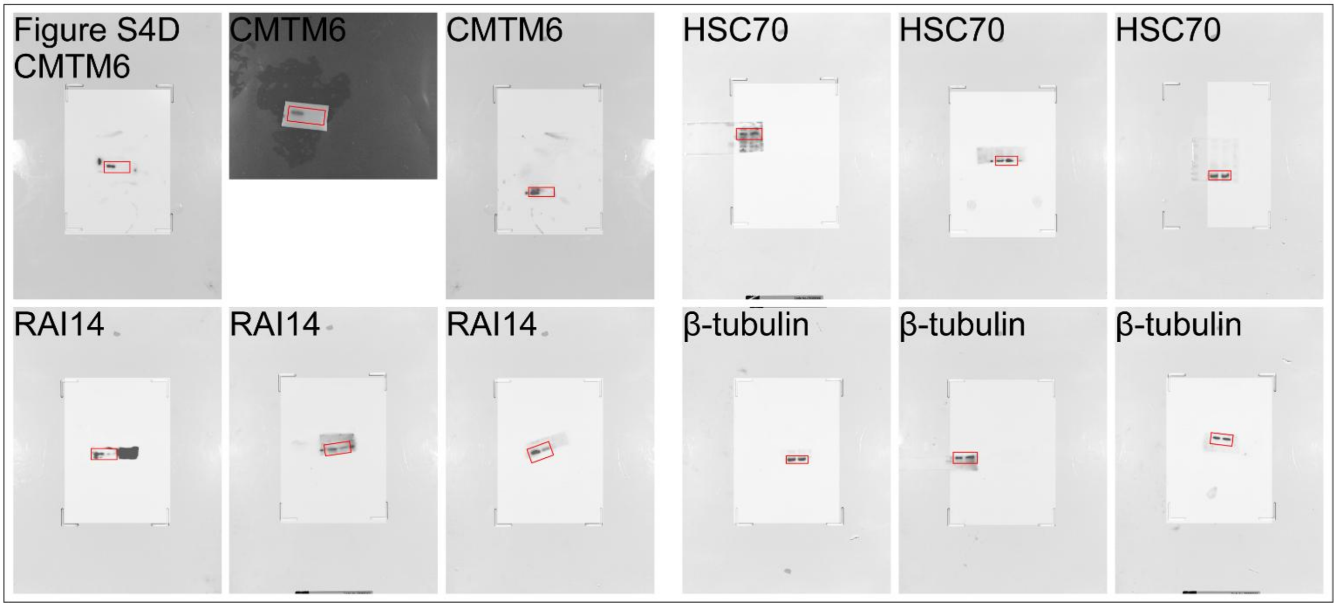
Figure S4D
CMTM6
CMTM6
CMTM6
HSC70
HSC70
HSC70
RAI14
RAI14
RAI14
β-tubulin
β-tubulin
β-tubulin

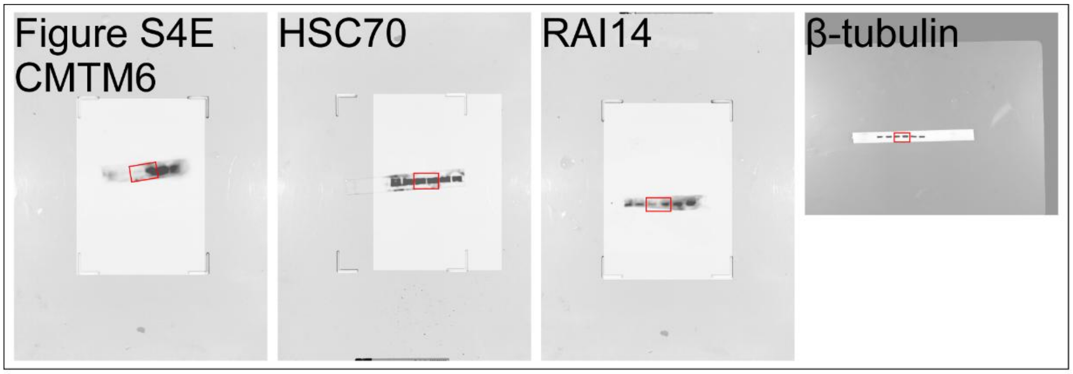
Figure S4E
CMTM6
HSC70
RAI14
β-tubulin

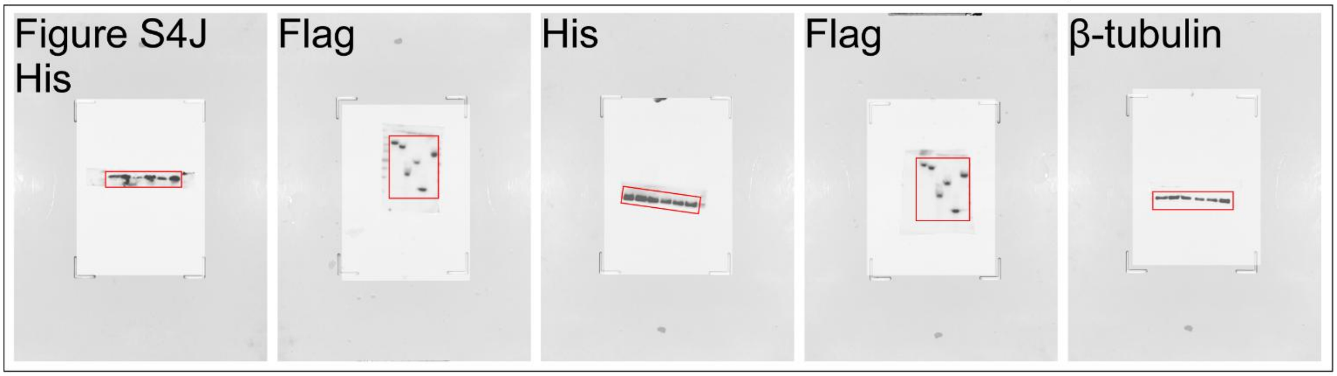
Figure S4J
His
Flag
His
Flag
β-tubulin

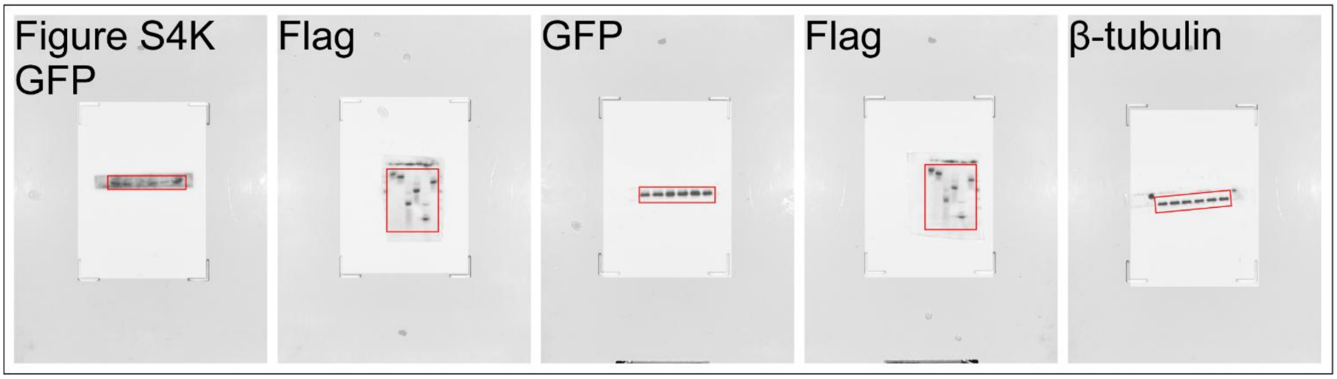
Figure S4K
GFP
Flag
GFP
Flag
β-tubulin

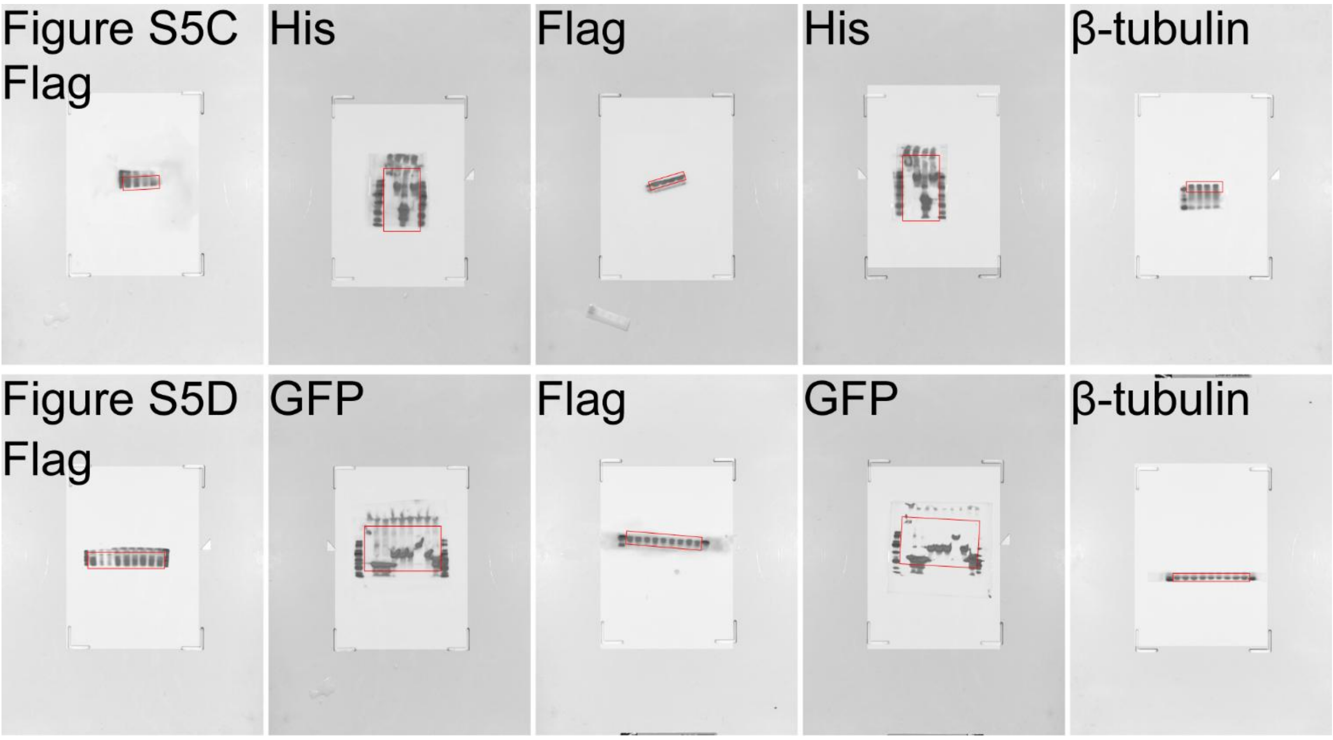
Figure S5C
Flag
His
Flag
His
β-tubulin
Figure S5D
Flag
GFP
Flag
GFP
β-tubulin
